# Rapid Cultivation of *Acanthamoeba* spp. Isolated from Environmental Samples Using Nanocomposite and Leech Saliva on Non-Nutrient Agar

**DOI:** 10.1007/s11686-025-01053-8

**Published:** 2025-05-12

**Authors:** Alican Bilden, Erdal Ertaş, Merve Kahraman, Servet Tural, Bilsen Tural, Muttalip Çiçek

**Affiliations:** 1 Ahi Evran University, Kırşehir, Turkey; 2 Batman University, Batman, Turkey; 3 Dicle University, Diyarbakır, Turkey

**Keywords:** *Acanthamoeba* spp., Nanotechnology, Non-Nutrient Agar, *Hirudo verbana*

## Abstract

**Objective:**

*Acanthamoeba* spp. are microscopic single-celled protozoa commonly found in the environment, particularly in soil, water sources, and dust. These parasites are associated with serious infections such as *Acanthamoeba* keratitis and granulomatous amebic encephalitis. Recent epidemiological studies have highlighted a significant increase in *Acanthamoeba* keratitis cases. Current treatment methods are generally effective only in the early stages and show limited success when applied late, emphasizing the urgent need for more effective therapeutic and cultivation approaches.

The laboratory cultivation of *Acanthamoeba* spp. is traditionally performed using axenic or monoxenic cultures. However, these methods have notable drawbacks, including the loss of virulence, reduced encystment capability, errors in bacterial inoculation, and time-consuming procedures. To address these limitations, nanotechnological approaches have been proposed. Nanotechnology offers innovative solutions for developing new drug formulations and diagnosing, preventing, and treating various diseases.

**Methods:**

In this study, we developed humic acid-coated magnetic nanocomposites capable of adhering to the cyst wall of *Acanthamoeba* spp. without causing damage. Experimental results demonstrated that these nanocomposites bind with high affinity to macromolecules on the cyst wall, facilitating the aggregation of parasites in the pellet. Additionally, biologically enriched leech saliva was incorporated into the culture medium to enhance the growth rate. Leech saliva provides a rich source of organic matter and bioactive molecules that promote cell division. The addition of leech saliva resulted in a significant increase in the growth rate of *Acanthamoeba* spp., with maximum growth density observed at 120 h.

**Results:**

These findings indicate that humic acid-coated magnetic nanocomposites and leech saliva -enriched culture media offer a promising alternative to conventional methods for the rapid and efficient cultivation of *Acanthamoeba* spp. Our study concludes that humic acid-coated magnetic nanocomposites effectively concentrate parasites, increasing their quantitative density, while leech saliva provides a nutrient-rich environment that stimulates trophozoite feeding and division.

**Conclusion:**

This study is noteworthy for presenting an innovative and effective method for the rapid laboratory cultivation and potential treatment of *Acanthamoeba* spp.

**Supplementary Information:**

The online version contains supplementary material available at 10.1007/s11686-025-01053-8.

## Introduction

*Acanthamoeba* spp. are opportunistic parasites widely distributed in natüre [1]. The life cycle of *Acanthamoeba* spp. consists of two stages: the actively dividing trophozoite stage and the cyst stage, which allows survival under adverse conditions. During the trophozoite phase, *Acanthamoeba* spp. divide by mitosis and feed primarily on bacteria, algae, yeast, or small organic particles through phagocytosis/pinocytosis. In unfavorable conditions, trophozoites transform into double-walled cyst forms [2]. In addition, it has been reported that some *Acanthamoeba* species, particularly *Acanthamoeba pyriformis*, can exhibit facultative sporocarp formation during their life cycle. This phenomenon suggests that under conditions of nutrient scarcity, drought, or other environmental stress factors, the organism can form spore-like structures as a survival strategy. Sporocarp structures are microscopic formations that bear spores and contribute to the organism’s ability to adapt to challenging environmental conditions [3]. Originally, *Acanthamoeba* spp. were classified based on cyst size and morphological characteristics. However, with the advancement of molecular techniques, particularly 18S rRNA gene sequencing, the classification system has been significantly revised. Currently, more than 30 *Acanthamoeba* species with clear taxonomic status have been identified through molecular methods. These species are known to cause a range of infections, including *Acanthamoeba* keratitis (AK), granulomatous amoebic encephalitis (GAE), and, less frequently, skin and pulmonary infections [4].

Epidemiological studies indicate that the incidence of acanthamoebiasis has been increasing year by year [5]. In recent years, particularly with the rising number of AK cases, researchers have made extensive efforts to characterize, classify, and study the molecular and cellular differentiation of *Acanthamoeba* species [6]. In this context, various culture media have been developed to facilitate the rapid production, purification, and identification of *Acanthamoeba* spp. However, these media present certain advantages as well as limitations. The cultivation of *Acanthamoeba* spp. can be performed using axenic and monoxenic methods. In axenic cultures, protozoa are most commonly grown in complex media such as PYG (Peptone-Yeast extract-Glucose medium), BSC (Brain–Heart Infusion Serum Culture), BHI (Brain Heart Infusion medium), and PPG (Proteose Peptone-Glucose medium). However, some *Acanthamoeba* spp. fail to grow in these axenic media, and prolonged use can result in virulence loss, encystation, or reduced pseudopodia formation [7, 8]. In monoxenic cultures, *Acanthamoeba* spp. are co-inoculated with an additional microorganism into the culture medium. The most commonly used monoxenic medium is non-nutrient agar (NNA) [9]. However, suboptimal conditions, such as improper preparation of the bacterial layer or the use of capsulated or pigmented bacteria, complicate the cultivation and detection of *Acanthamoeba* spp. [10]. Such cultivation difficulties, combined with the clinical overlap with other infectious keratitis, complicate the diagnostic process. The clinical presentation of AK can resemble fungal or viral keratitis, which may complicate accurate diagnosis and contribute to delays in initiating appropriate treatment [11]. Therefore, there is an urgent need for the development of more effective media for the rapid diagnosis, production, and purification of *Acanthamoeba* spp. [12].

Different methods have been explored to address the limitations of existing diagnostic and culture techniques. One promising approach is nanotechnology, which has recently gained traction in the medical field [13]. Magnetic nanocomposites (MNPs) produced through this technology offer natural advantages, such as ease of surface modification, precise size control, and large surface area [14].

The saliva of medicinal leeches (LS), used therapeutically since ancient times, contains over 100 proteins, peptides, and various microorganisms [15]. Among these microorganisms, *Aeromonas hydrophila* is predominant on the leech's body surface, as well as in its oral and gut flora [16]. The proteins include bioactive components that promote cell division, notably the hyaluronidase enzyme, which degrades the extracellular matrix, enhancing cell motility and division [17].

This study aims to address the limitations of conventional culture media by employing a nanotechnology-based method in combination with protein-rich leech saliva. The proposed approach involves the functionalization of magnetic nanocomposite particles with humic acid molecules, enabling interaction with the cell surface of *Acanthamoeba* spp. to form stable nanocomposite–*Acanthamoeba* spp. complexes. Subsequently, the addition of leech saliva serves to enhance the nutritional environment, thereby promoting the rapid and efficient growth of *Acanthamoeba* spp.

## Materials and Methods

### Sample Collection

Water and soil samples were collected from Lake Hılla, located within the borders of Kırşehir Province (https://maps.app.goo.gl/93AumssoCKeemBiW6*).* These samples were mixed, converted into sludge, and filtered. From the resulting filtrate, 1 g and 1 mL of distilled water were taken and thoroughly mixed in a dilution tube. This mixture was referred to as"resin". Three separate tubes were prepared from this mixture, and all experimental processes were conducted in triplicate to ensure reproducibility.

### Synthesis of Humic Acid-Functionalized Magnetic Nanocomposites (MNPs@HA)

The coating of MNPs with humic acid (HA) was achieved with modifications to a method described in the literature [18]. For the synthesis, 3.0 g of FeCl_3_•6H_2_O was dissolved in 50 mL of deionized water at room temperature under magnetic stirring. To prevent hydrolysis, 3–4 drops of 36% HCl solution were added while stirring continued. Subsequently, 2.1 g of FeSO_4_•7H_2_O was added to the Fe^3+^ containing solution, and the reaction temperature was gradually increased to 80 °C. After 30 min, 5 mL of 26% ammonium hydroxide solution was added, and the mixture was stirred for an additional 60 min. Following this, 0.25 g of humic acid dissolved in 25 mL of distilled water was added dropwise to the mixture. After the addition of the humic acid solution, stirring was ceased after 60 min, and the mixture was allowed to cool to room temperature.

**Fig. 1:**
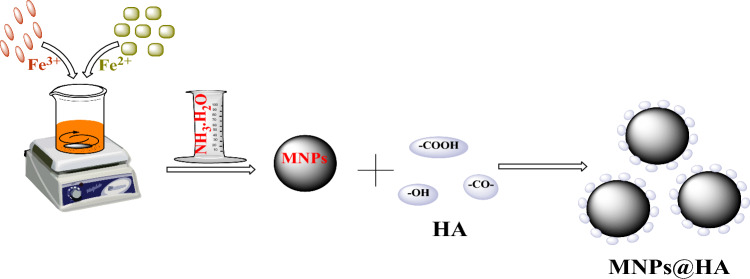
Synthesis of Humic Acid-Coated Magnetic Nanocomposites (MNPs@HA)

Once cooled, the MNPs@HA were separated from the solution using a neodymium magnet. To remove impurities from the MNPs@HA nanocomposites, the material was washed several times with distilled water and subsequently dried using a lyophilizer (Fig. 1).

### Preparation of Non-Nutrient Agar (NNA) Escherichia coli Plates

The preparation of non-nutrient agar began with the weighing and preparation of the required materials. A total of 17 g of agar powder and 1 L of distilled water were measured. The distilled water was heated in an erlenmeyer flask, and the agar powder was gradually added while continuously stirring to ensure complete dissolution. The agar solution was then sterilized in an autoclave at 121 °C for 15–20 min. After sterilization, the solution was allowed to cool slightly, and when the temperature reached approximately 45–50 °C, it was poured into sterile petri dishes. The agar was left to solidify, and the prepared agar plates were stored under sterile conditions until use. Before use, the surface of the plates was inoculated with an *Escherichia coli* suspension equivalent to 4.5 McFarland, using a sterile swab [2]. The plates prepared in this manner were made ready for use in experimental procedures.

### Feeding of Leeches and Collection of Leech Saliva (LS)

Starved medicinal leeches of the species *Hirudo verbana* were fed with a sterile synthetic solution containing 0.15 M sodium chloride and 0.001 M arginine at 37 °C. After the leeches were fully fed with this solution, they were placed into a sealed plastic tube submerged in an ice bath. The leeches were kept on ice for 15–20 min to induce paralysis. This process allowed the leeches to expel all the ingested solution along with their salivary secretions. The expelled solution was collected using a vacuum and transferred into 50 mL falcon tubes, then stored at − 80 °C for experimental use. Finally, the leeches were placed in warm water (37 °C) for 15–30 min to recover their activity [15]. For this study, a total of 30 leeches of standard sizes were used for experimental procedures.

### Isolation and Cultivation of Acanthamoeba spp.

**Fig. 2:**
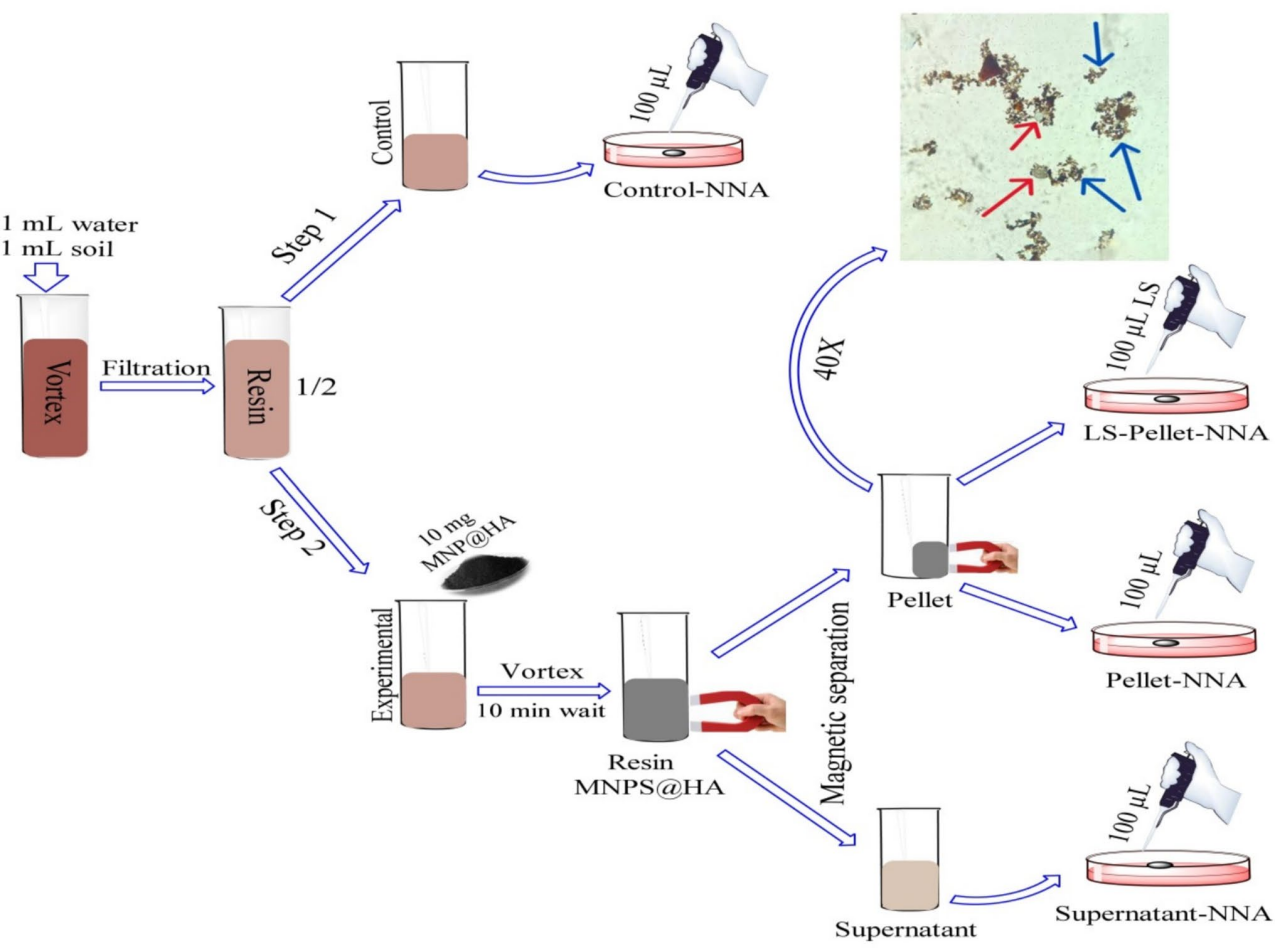
Experimental setup for the isolation and cultivation of Acanthamoeba spp. Resin was prepared by filtering and vortexing a soil–water mixture and then divided into Control and Experimental conditions. In the Experimental condition, 10 mg of MNP@HA was added, followed by vortexing and magnetic separation to isolate the pellet and supernatant. Subsequently, 100 µL from each condition was plated onto specific NNA media types as shown in the figure: Control-NNA, LS-Pellet-NNA, Pellet-NNA, and Supernatant-NNA. Microscopic observations (40X) confirmed the interaction between MNP@HA and Acanthamoeba spp. cysts (red arrows: cysts, blue arrows: MNP@HA)

*Step 1:* The prepared resin was divided into two equal portions and transferred into two identical tubes. The first tube was designated as the"Control Tube", while the second was labeled as the"Experimental Tube"(Fig. 2). From the Control Tube, 100 µL of resin was directly taken without any treatment and inoculated at the center of a NNA plate, referred to as the Control-NNA medium.

**Fig. 3:**
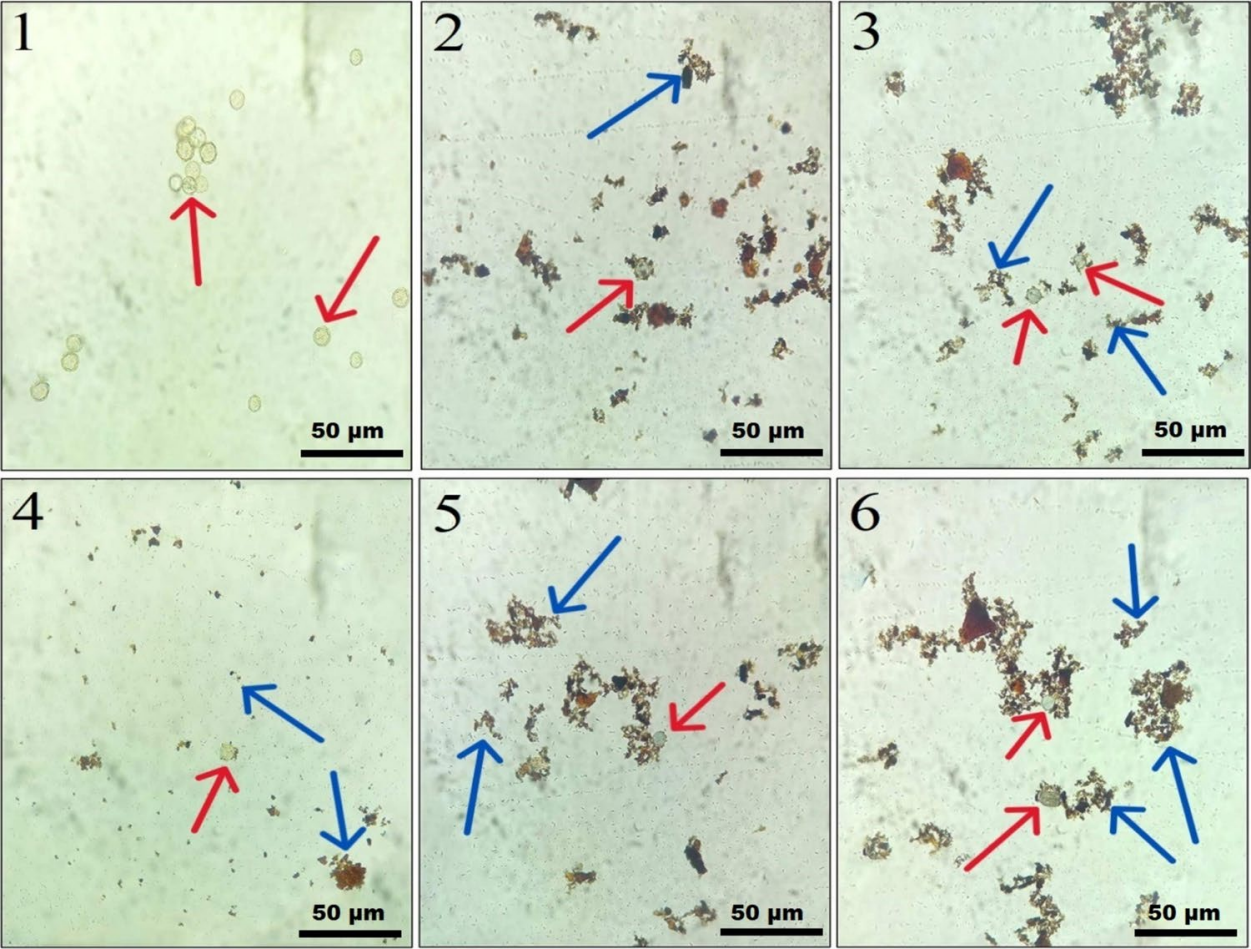
Microscopic images illustrating the interaction of MNPs@HA with *Acanthamoeba* spp. in the pellet (light microscope, 40X). Panel 1 shows only *Acanthamoeba* spp. cysts, whereas Panels 2–6 display the interaction between MNPs@HA and *Acanthamoeba* spp.. The red arrows indicate *Acanthamoeba* spp. cysts, and the blue arrows indicate MNPs@HA

**Fig. 4:**
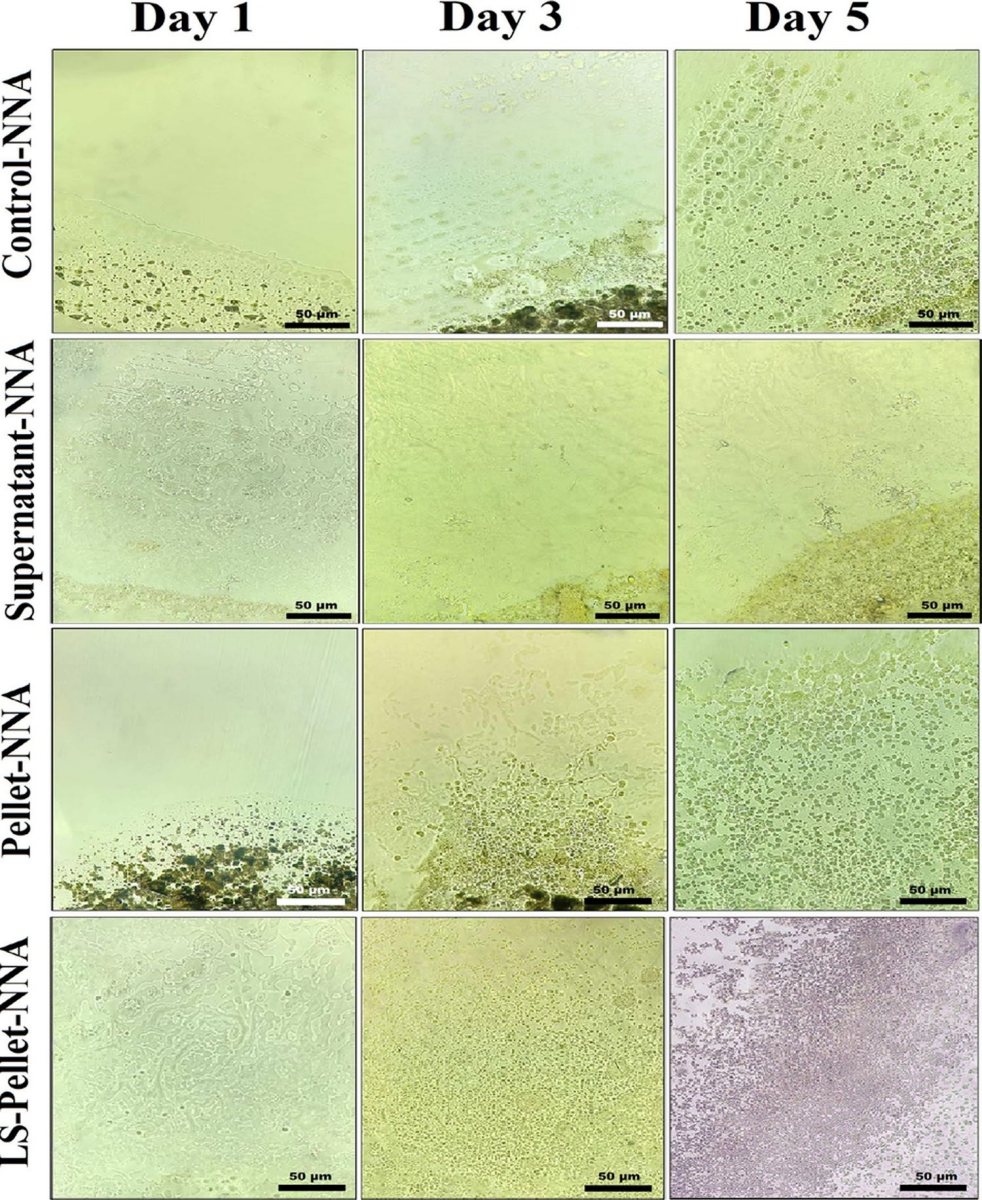
Growth rates of *Acanthamoeba spp.* in Control-NNA, Supernatant-NNA, Pellet-NNA, and LS-Pellet-NNA media across Days 1, 3, and 5. Images captured using an inverted microscope (40X)

**Fig. 5:**
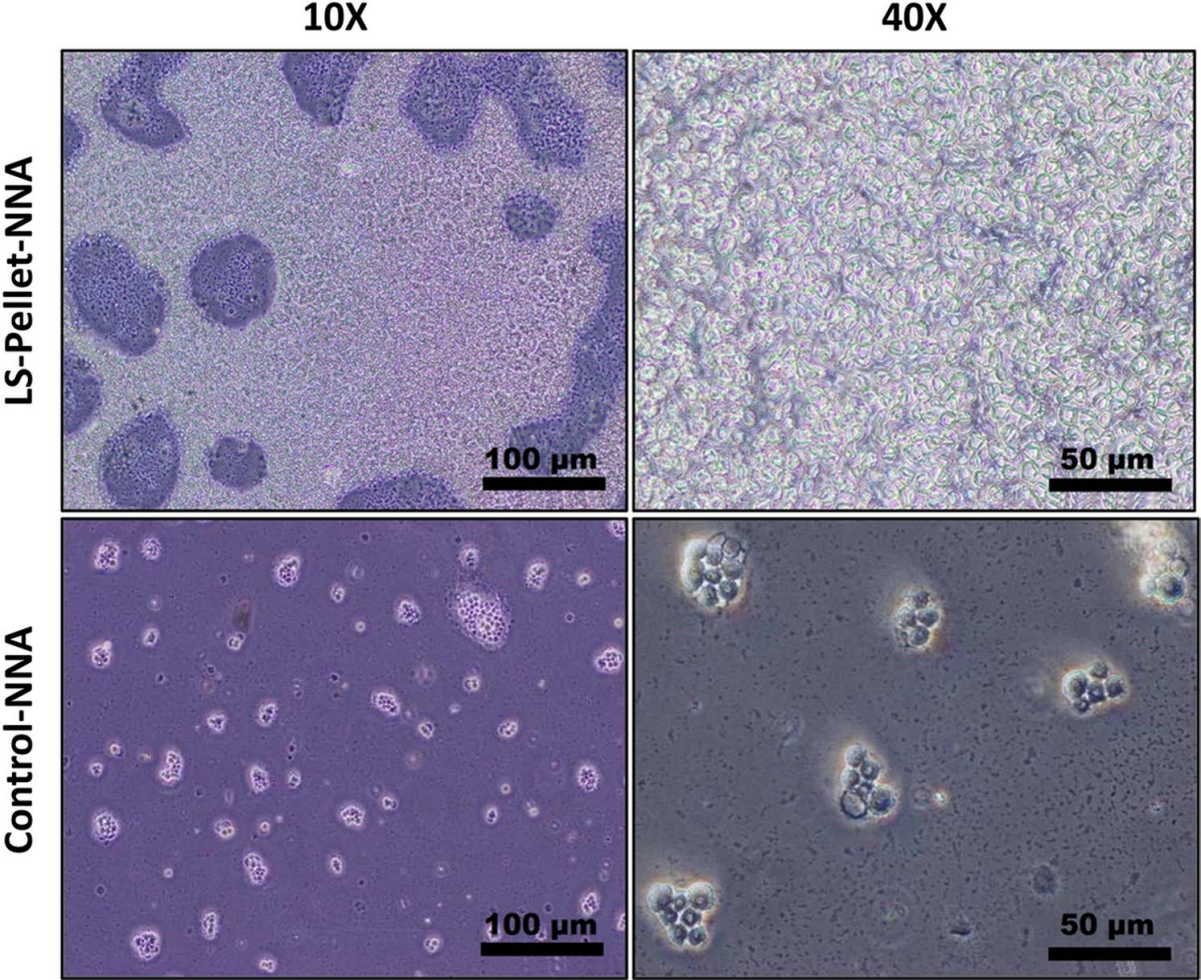
Microscopic images of parasites grown in LS-Pellet-NNA and Control-NNA media at 120 h, captured under an inverted microscope. Images were taken at 10X and 40X magnifications. LS-Pellet-NNA shows higher growth density, whereas Control-NNA displays larger but less dense parasite populations

*Step 2:* In the Experimental Tube, 10 mg of MNPs@HA was added to the resin. The tube was vortexed for 10 min to facilitate the interaction between MNPs@HA and *Acanthamoeba* spp. Afterward, the Experimental Tube was exposed to a magnetic field to verify the presence of the MNPs@HA-*Acanthamoeba* complex by separating the pellet and supernatant. Preparations were made from both the pellet and supernatant, and these were examined under a light microscope at 40X magnification (Fig. 3). Concurrently, inoculations were performed. The supernatant was inoculated onto one plate (Supernatant-NNA medium), and the pellet was inoculated onto two separate plates. One of the pellet-derived plates was left untreated and designated as the Pellet-NNA medium, while 100 µL of LS was added to the second pellet-derived plate, which was designated as the LS-Pellet-NNA medium. All prepared plates were incubated at room temperature, and their growth rates were monitored daily for five consecutive days at the same time using an inverted microscope with 10X and 40X objectives (Figs. 4 and 5). The entire experimental procedure was conducted in triplicate.

## Results

To evaluate the formation of the MNPs@HA-*Acanthamoeba* complex, fresh preparations were made from both the supernatant and the pellet. Observations under a light microscope at 40X magnification showed no interaction of MNPs@HA-*Acanthamoeba* in the supernatant, whereas such interaction was observed and documented in the pellet (Fig. 3). Following this observation, inoculations from both the supernatant and pellet were performed onto NNA plates. After 24 h, growth was observed exclusively in the LS-Pellet-NNA medium under an inverted microscope. At 72 h, growth was also noted in the Pellet-NNA and Control-NNA media. However, the parasites in the LS-Pellet-NNA medium were observed to be more numerous but smaller compared to those in the Pellet-NNA and Control-NNA media. Additionally, the growth density in the Pellet-NNA medium was higher than that in the Control-NNA medium. By the end of 120 h, no growth was observed in the Supernatant-NNA medium, whereas growth persisted in the other media and was documented (Figs. 4 and 5).

## Discussion

The current study presents a novel approach for the improved cultivation of *Acanthamoeba* spp. by combining MNPs@HA with biologically enriched LS. This strategy addresses the long-standing challenges in isolating amoebae from complex environmental samples, where standard culture techniques often fail. Given the growing incidence of AK globally—especially among contact lens users [19]—developing efficient and reproducible culture methods has become increasingly important for both clinical diagnosis and epidemiological surveillance [12, 20]. Delayed diagnosis, inappropriate use of corticosteroids, and progressive symptoms can significantly worsen the ocular prognosis in AK cases. Moreover, current treatment regimens show limited efficacy, particularly if not initiated during the early stages of infection [21]. These clinical challenges have led researchers to intensify efforts in developing novel therapeutic agents and improving diagnostic protocols for acanthamoebiasis [22, 23]. Within this context, rapid and reliable cultivation of *Acanthamoeba* spp. has become a critical prerequisite for both drug discovery and experimental modeling. While axenic and monoxenic methods have long been used in *Acanthamoeba* spp. cultivation, their practical limitations remain a significant barrier in routine diagnostics [4, 8, 24]. The approach employed in this study offers a time-saving, cost-effective, and biologically supportive alternative to overcome these challenges.

It is suggested that nanotechnological methods can be utilized to overcome these disadvantages. This technology has emerged as a novel approach to explore new pathways in the diagnosis, prevention, and treatment of various diseases where traditional methodologies have proven ineffective [25]. In the context of acanthamoebiasis, the development of nanoparticles capable of binding to or effectively disrupting the cyst wall has become a key area of research focus [4, 26]. These therapeutic advancements in nanotechnology also contribute to sustaining the relevance and urgency of approaches aimed at the isolation and rapid diagnosis of the parasite. Inspired by these advancements, our project team designed MNPs@HA that can adhere to the surface of the cyst wall of *Acanthamoeba* spp. without causing damage. Observations under a light microscope revealed that this nanocomposite, used in experimental studies, binds to macromolecules located on the cyst wall with high affinity (Fig. 3). The literature reports that humic acid-coated nanocomposites are effective in removing environmental toxins and interacting with microbial cell surfaces [25]. In another study supporting our work, ciprofloxacin, commonly used in combination therapy for acanthamoebiasis, was utilized for its biocompatibility. It was functionalized with selenium nanoparticles (SeNPs) and successfully attached to the surface of trophozoite cells [22]. In another study, it was reported that silver and gold nanoparticles modified with tannic acid exhibited strong adhesion to the cell surface of trophozoites, thereby effectively blocking the encystation phase [27]. Another study reported that cationic carbosilane dendrimers exhibited strong affinity for the external surfaces of cysts and trophozoites, causing significant morphological alterations [28].

Consistent with the literature, the MNPs@HA designed by our team demonstrated that while no growth was observed in the supernatant culture, very dense growth was observed in the pellet culture (Figs. 4 and 5). This result highlights the selective binding and purification capacity of the nanocomposite. Previous studies have also shown that magnetic nanocomposites are effective in purifying biological samples [29]. To enhance the growth rate in the medium containing the pellet, LS enriched with bacteria and organic matter was added. Leeches, being aquatic organisms, naturally harbor a diverse bacterial microbiota, predominantly comprising gram-negative *Aeromonas* species [16]. Moreover, LS has been reported to contain over 100 biologically active molecules, including components that promote cell proliferation [30]. In particular, the hyaluronidase enzyme found in LS is known to degrade the extracellular matrix, thereby facilitating cell motility and division [31]. In our study, following the formation of the MNPs@HA–*Acanthamoeba* complex and the concentration of the parasites in pellet form, LS—with its biologically enriched content—was added to the Pellet-NNA medium. This fluid, rich in organic matter, microbial content, and mitogenic components, significantly enhanced the proliferation of *Acanthamoeba* spp. trophozoites (Figs. 4 and 5). These findings suggest that LS acts as a biological stimulant that supports the *in vitro* growth of *Acanthamoeba* under culture conditions.

Previous studies have highlighted the variability in *Acanthamoeba* growth across different axenic culture systems. For instance, one study reported that the highest parasite density was achieved in TYM medium after 48 h, whereas the control group maintained in NNA exhibited the lowest density even after 72 h [32]. Another study comparing the growth of various *Acanthamoeba* species across multiple media over a 14-day period found that RPMI-FBS supported the most rapid growth during the early phase, while growth on NNA remained slow [33]. Additional reports have noted that PYG and TYI-S-33 media, in combination with calcofluor white staining and fluorescence microscopy, may aid in the diagnosis of *Acanthamoeba* spp., with PYG medium being cited as a simple, cost-effective, and efficient option for cultivation and detection [34]. In contrast, one study found that PYG was ineffective for cultivating *Acanthamoeba* spp., while TYI-S-33 provided successful results [31]. These comparisons illustrate the variability and inconsistency across axenic systems, which our modified NNA-based method aims to overcome.

In our study, growth was observed in the LS-Pellet-NNA medium at 24 h, while growth in the Pellet-NNA and Control-NNA media was noted at 72 h. By the end of 72 h, the growth density in the Pellet-NNA medium was higher than that in the Control-NNA medium. Supporting this result, at 120 h, the maximum growth was observed in the LS-Pellet-NNA medium, followed by substantial growth in the Pellet-NNA medium, compared to the Control-NNA medium (Figs. 4 and 5). However, parasites grown in the LS-Pellet-NNA medium were found to be smaller than those grown in other media (Figs. 4 and 5). Rapid cell proliferation in protozoa may affect growth and maturation, potentially resulting in morphological alterations such as changes in cell size. This observation likely reflects an underlying mechanism associated with biochemical and biological processes. Although the exact cause remains unclear, some studies in the literature appear to suggest possible explanations for this phenomenon. For example, one study reported that amoebic morphology can vary depending on the culture environment [8]. Additionally, another study demonstrated that overexpression of AcSir2 in *Acanthamoeba castellanii* trophozoites resulted in increased proliferation accompanied by alterations in cellular characteristics [35]. Taken together, these findings support the notion of a potential trade-off between rapid proliferation and complete cellular maturation. Another study, although not explicitly designed to test this, has suggested that increasing cell density in protozoan cultures may lead to restricted nutrient availability, which could, in turn, influence trophozoite morphology, including reductions in cell size. Environmental factors such as substrate composition and cellular crowding have also been shown to affect the behavior and aggregation dynamics of *Acanthamoeba* spp. [36]. Another study examined the effects of metal nanoparticles in biological systems, providing a general framework on how rapid growth processes can influence metabolic balances [14]. The general findings on the effects of nanoparticles on biological growth can be referenced to explain the relationship between rapid growth and metabolic suppression observed in our study. In this study, the ability of LS to achieve maximum growth density at the end of 120 h highlights its potential to provide a faster and more efficient production environment compared to existing culture methods. It has been reported that the bioactive proteins in LS stimulate cell metabolism, thereby accelerating the growth process [17].

In the literature, various culture media have been proposed for the rapid cultivation of *Acanthamoeba* spp., many of which remain subject to ongoing debate due to their limitations and inconsistent performance. In light of these challenges, the present study suggests that the LS-Pellet-NNA medium may enhance the efficiency of cultivation by enabling the concentration of parasites through MNPs@HA, leading to a visibly increased parasite density compared to traditional NNA media. This elevated parasite load, when combined with the nutrient-rich content of leech saliva, appears to positively influence the growth rate and reduce the overall cultivation time. To the best of our knowledge, this study is the first to explore the integrated use of humic acid-coated magnetic nanocomposites and leech saliva in a single cultivation platform for *Acanthamoeba* spp., offering a potentially valuable alternative to conventional methods.

## Conclusions

This study demonstrated that the synergistic use of nanotechnology and biological additives offers a promising approach for the rapid laboratory cultivation of *Acanthamoeba* spp. Based on the findings, the humic acid-coated magnetic nanocomposites and the leech saliva-enriched medium proposed herein may serve as effective alternatives to conventional culture media for the efficient cultivation of *Acanthamoeba* spp. Future studies will aim to optimize this method and evaluate its applicability across various environmental samples. Moreover, the accuracy and reproducibility of the method in clinical applications are currently under investigation. However, several limitations of this study should be acknowledged. First, the findings are restricted to environmental isolates, and the method's performance has not yet been validated using clinical samples. Furthermore, the mechanisms of action of the employed nanocomposites and leech saliva have not been explored at the molecular level, as no biochemical or genetic analyses were conducted. Observed changes in cell morphology were assessed only descriptively, without mechanistic clarification. Additionally, the proposed method was tested exclusively under *in vitro* conditions, with no evaluation of *in vivo* applicability or toxicological safety. Thus, further comprehensive studies are warranted to substantiate and expand upon these initial findings.

## Supplementary Information

Below is the link to the electronic supplementary material.

Supplementary file1 (ZIP 208058 KB)

## Data Availability

All data included in the manuscript are available.
