# Supplementary figures and images for: Rapid Cultivation of Acanthamoeba spp. Isolated from Environmental Samples Using Nanocomposite and Leech Saliva on Non-Nutrient Agar
Source: Acta Parasitol. 2025 May 12;70(3):104. doi: 10.1007/s11686-025-01053-8 (PMC12069485; doi:10.1007/s11686-025-01053-8)

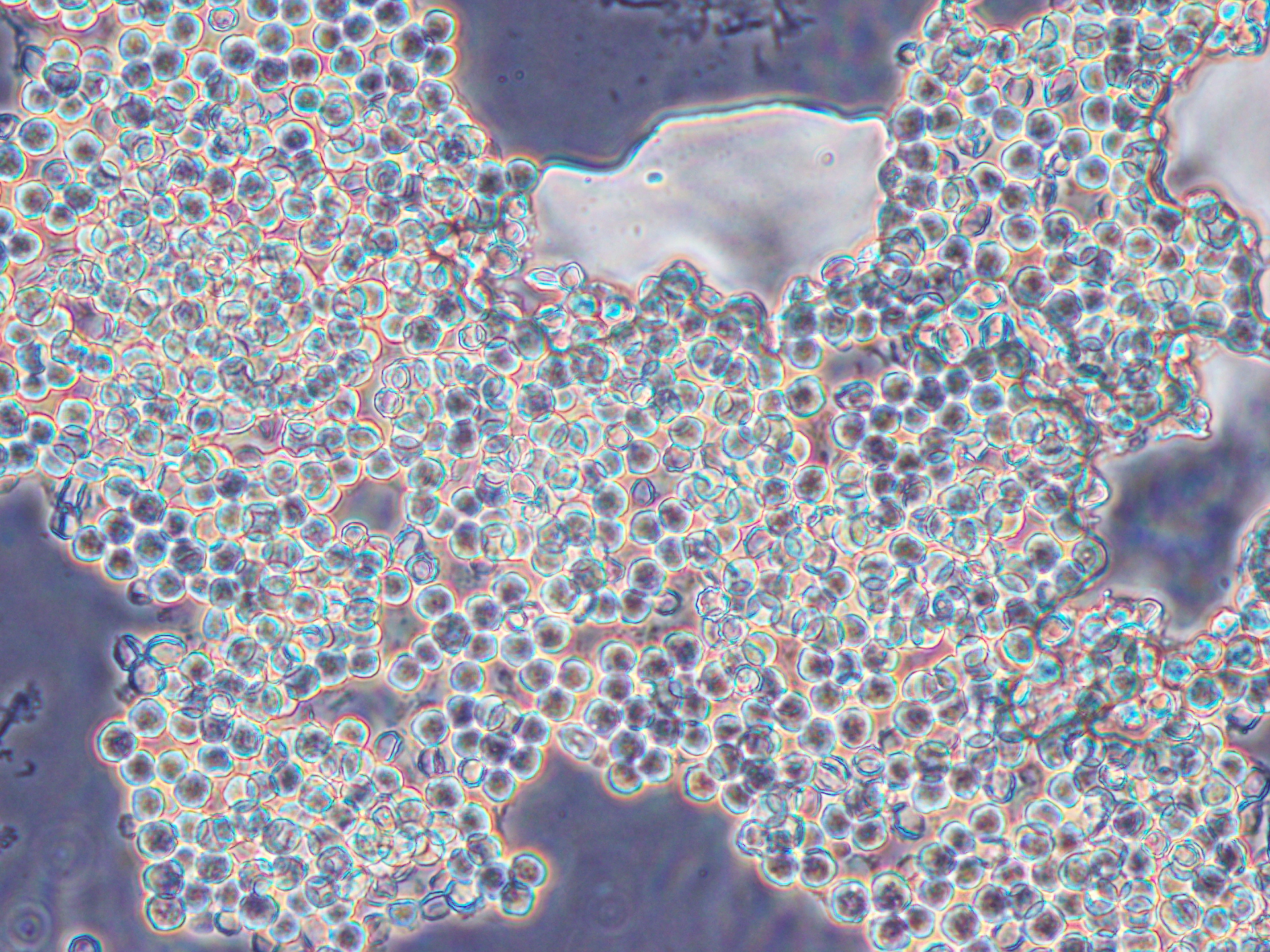

Supplement: Supplementary file 1 — Supplementary file1 (ZIP 208058 KB) [file 11686_2025_1053_MOESM1_ESM.zip › Supplementary_Figure3_4_5_MicroscopyImages/Cyst-1.JPG]

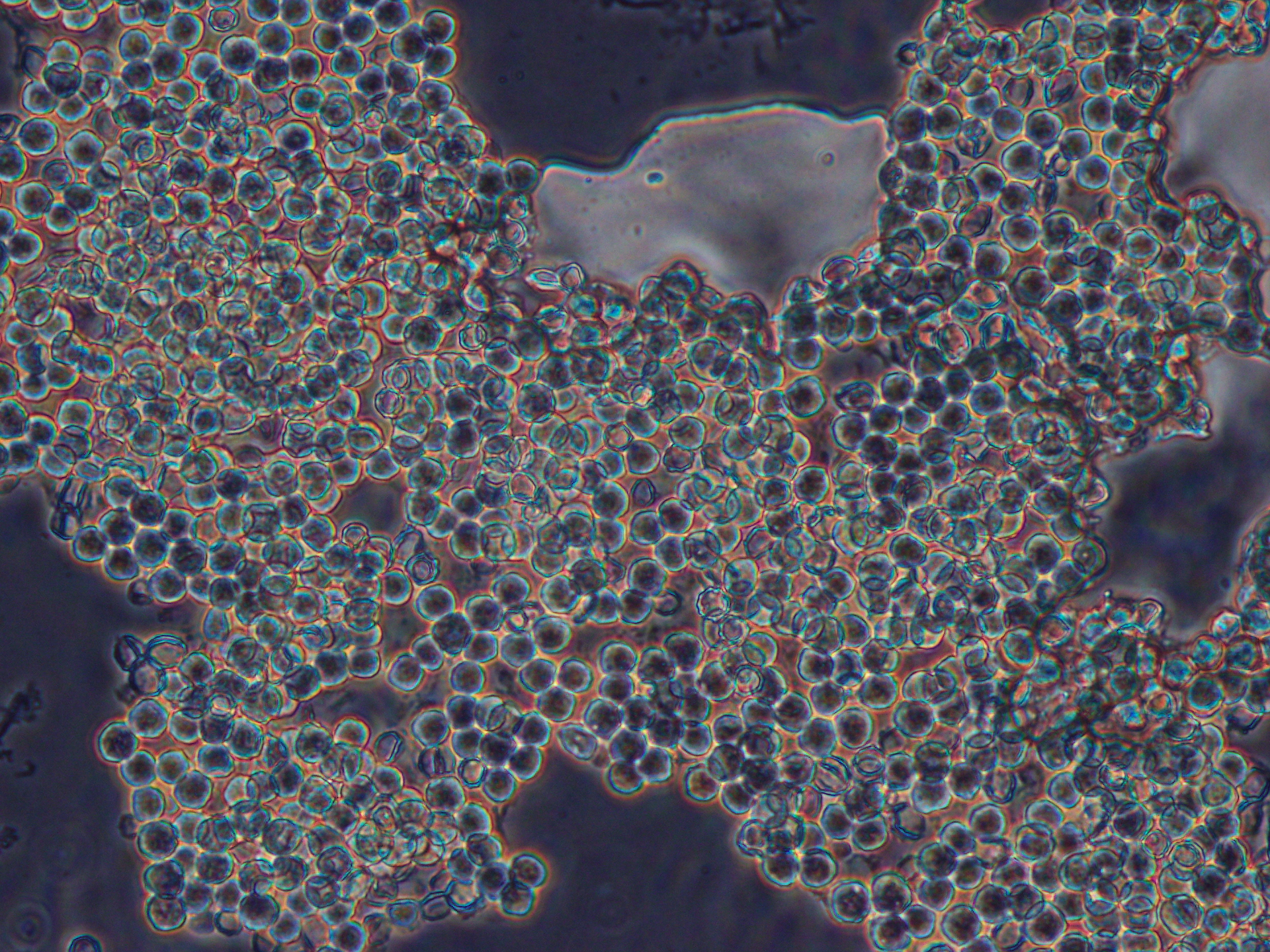

Supplement: Supplementary file 1 — Supplementary file1 (ZIP 208058 KB) [file 11686_2025_1053_MOESM1_ESM.zip › Supplementary_Figure3_4_5_MicroscopyImages/Cyst-10.JPG]

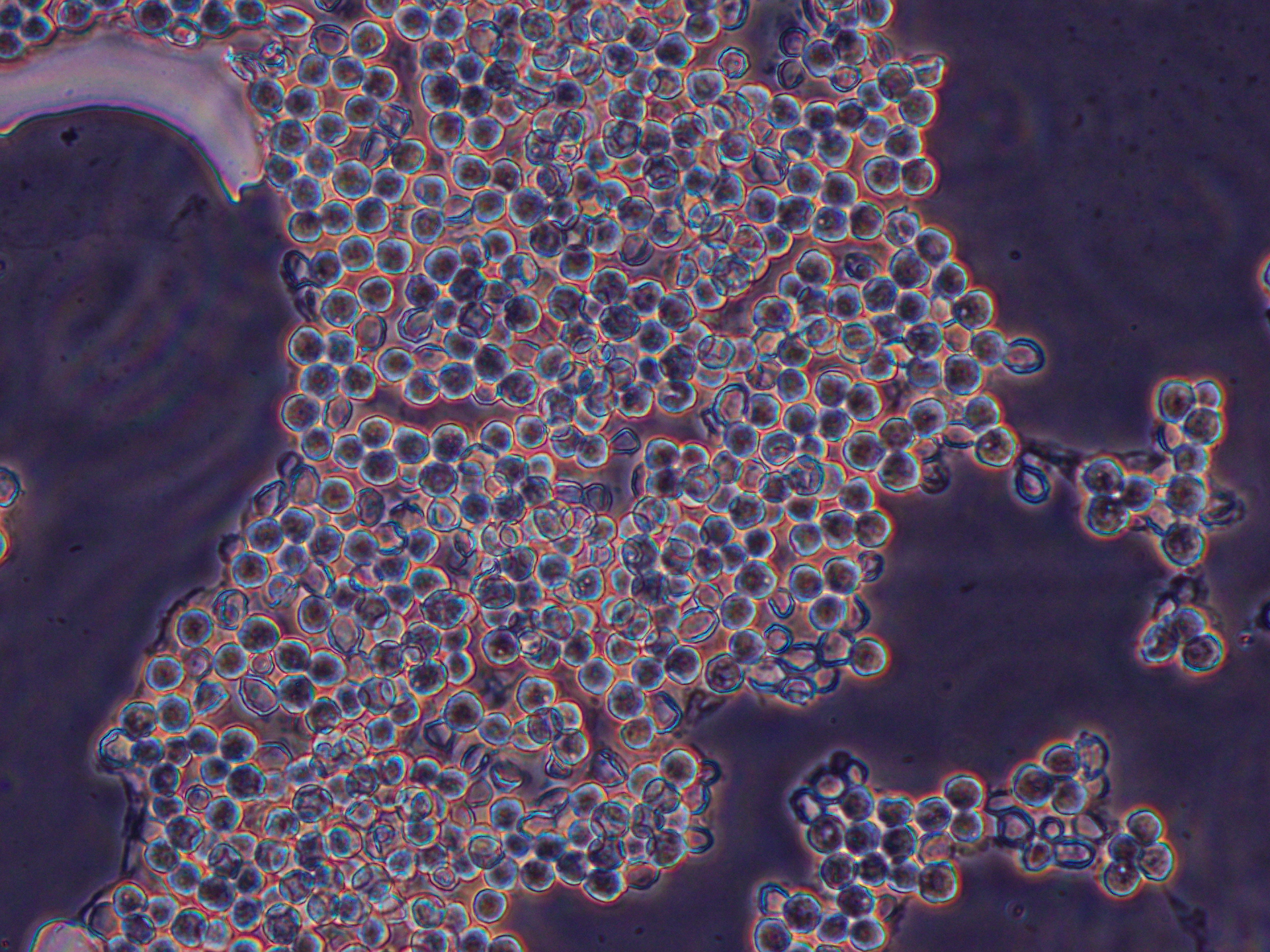

Supplement: Supplementary file 1 — Supplementary file1 (ZIP 208058 KB) [file 11686_2025_1053_MOESM1_ESM.zip › Supplementary_Figure3_4_5_MicroscopyImages/Cyst-11.JPG]

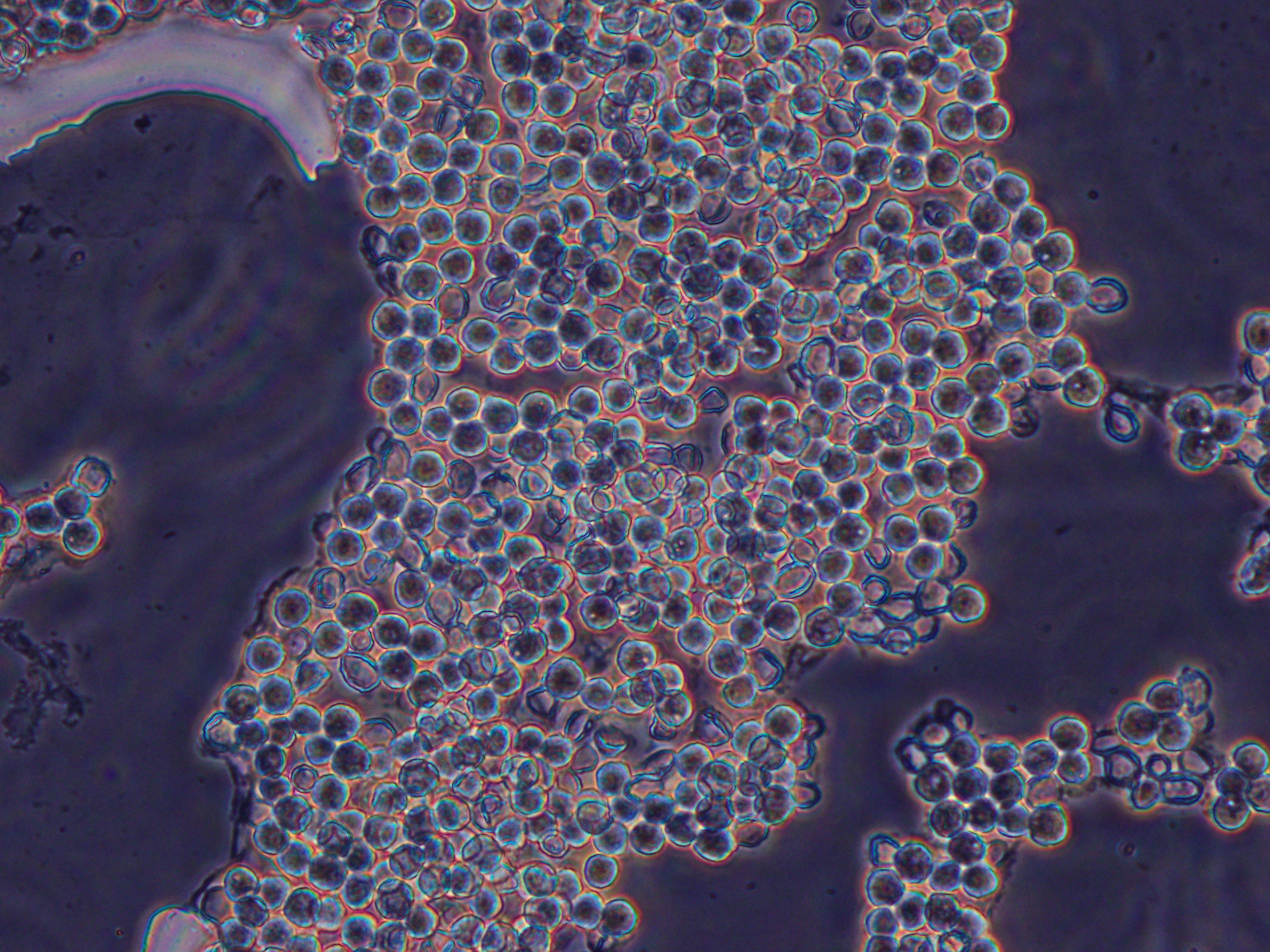

Supplement: Supplementary file 1 — Supplementary file1 (ZIP 208058 KB) [file 11686_2025_1053_MOESM1_ESM.zip › Supplementary_Figure3_4_5_MicroscopyImages/Cyst-12.JPG]

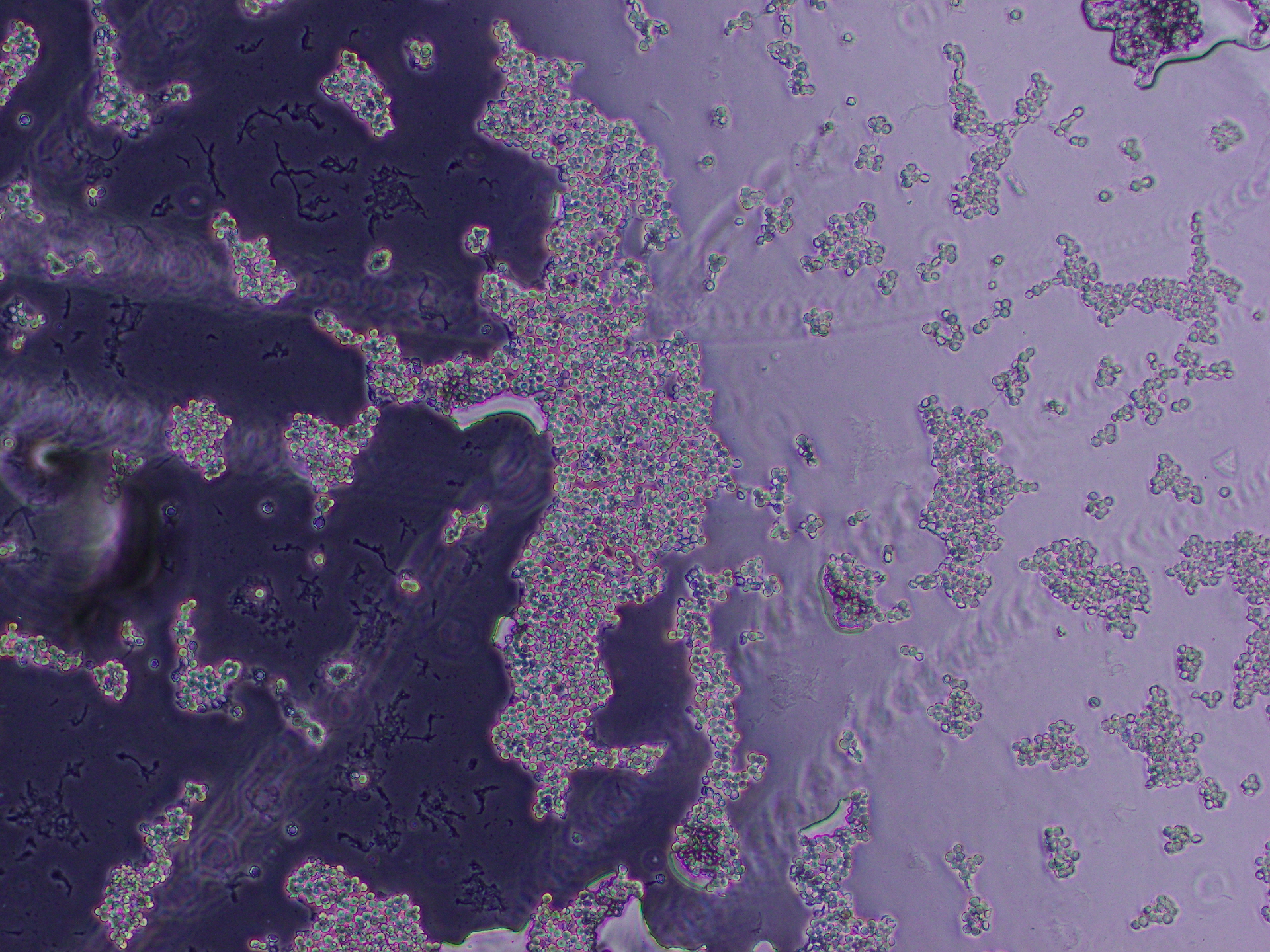

Supplement: Supplementary file 1 — Supplementary file1 (ZIP 208058 KB) [file 11686_2025_1053_MOESM1_ESM.zip › Supplementary_Figure3_4_5_MicroscopyImages/Cyst-13.JPG]

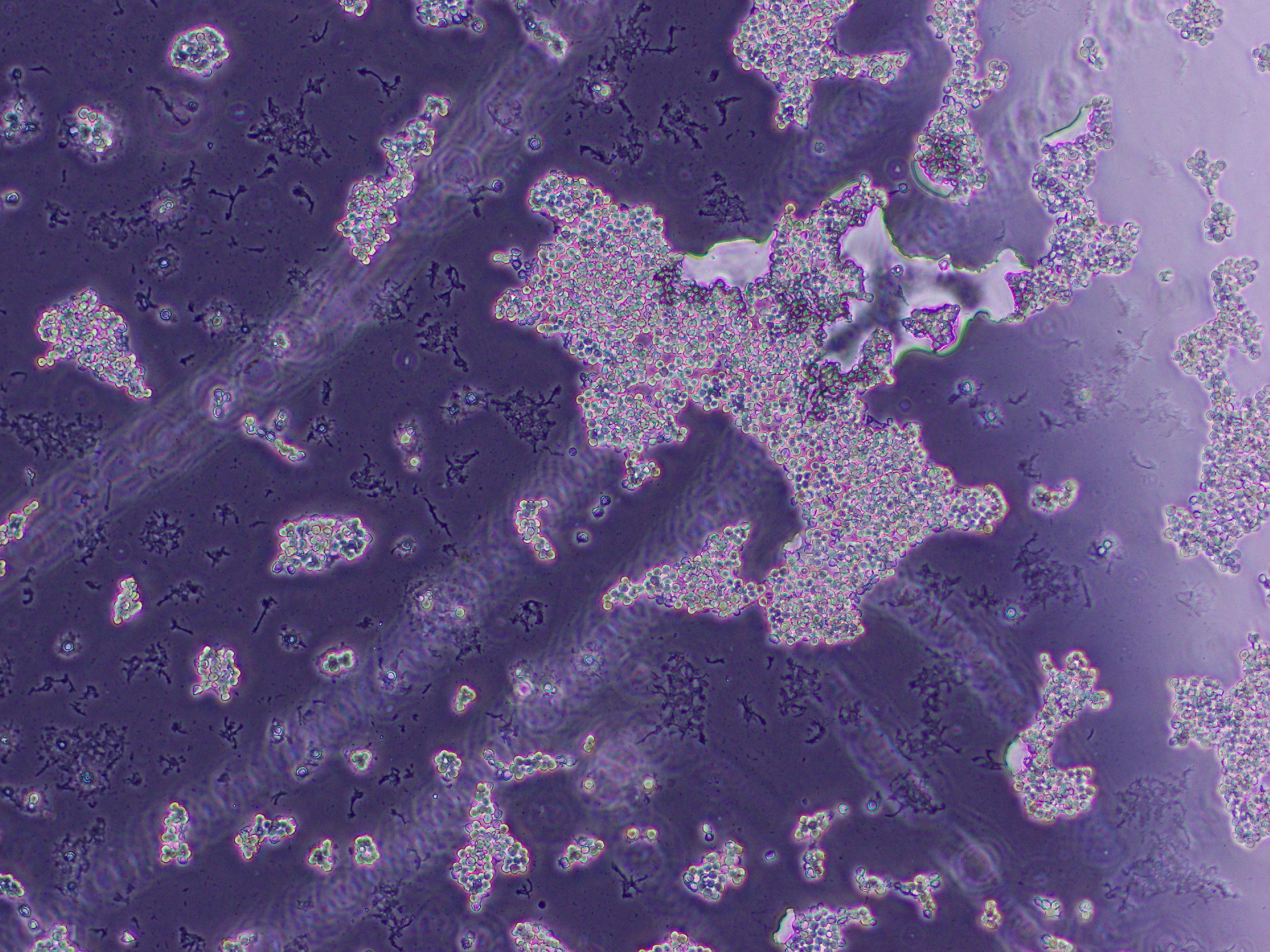

Supplement: Supplementary file 1 — Supplementary file1 (ZIP 208058 KB) [file 11686_2025_1053_MOESM1_ESM.zip › Supplementary_Figure3_4_5_MicroscopyImages/Cyst-14.JPG]

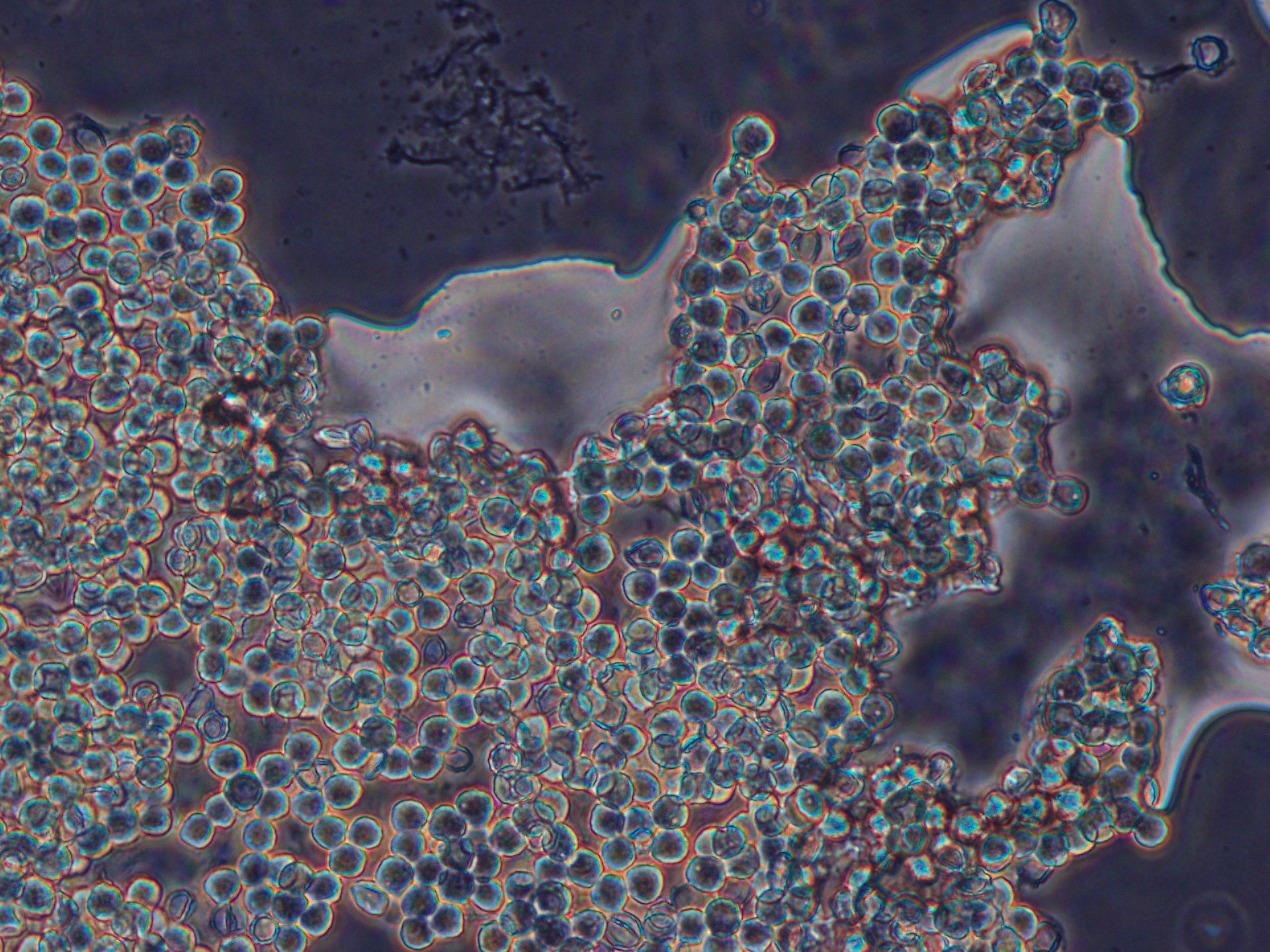

Supplement: Supplementary file 1 — Supplementary file1 (ZIP 208058 KB) [file 11686_2025_1053_MOESM1_ESM.zip › Supplementary_Figure3_4_5_MicroscopyImages/Cyst-15.JPG]

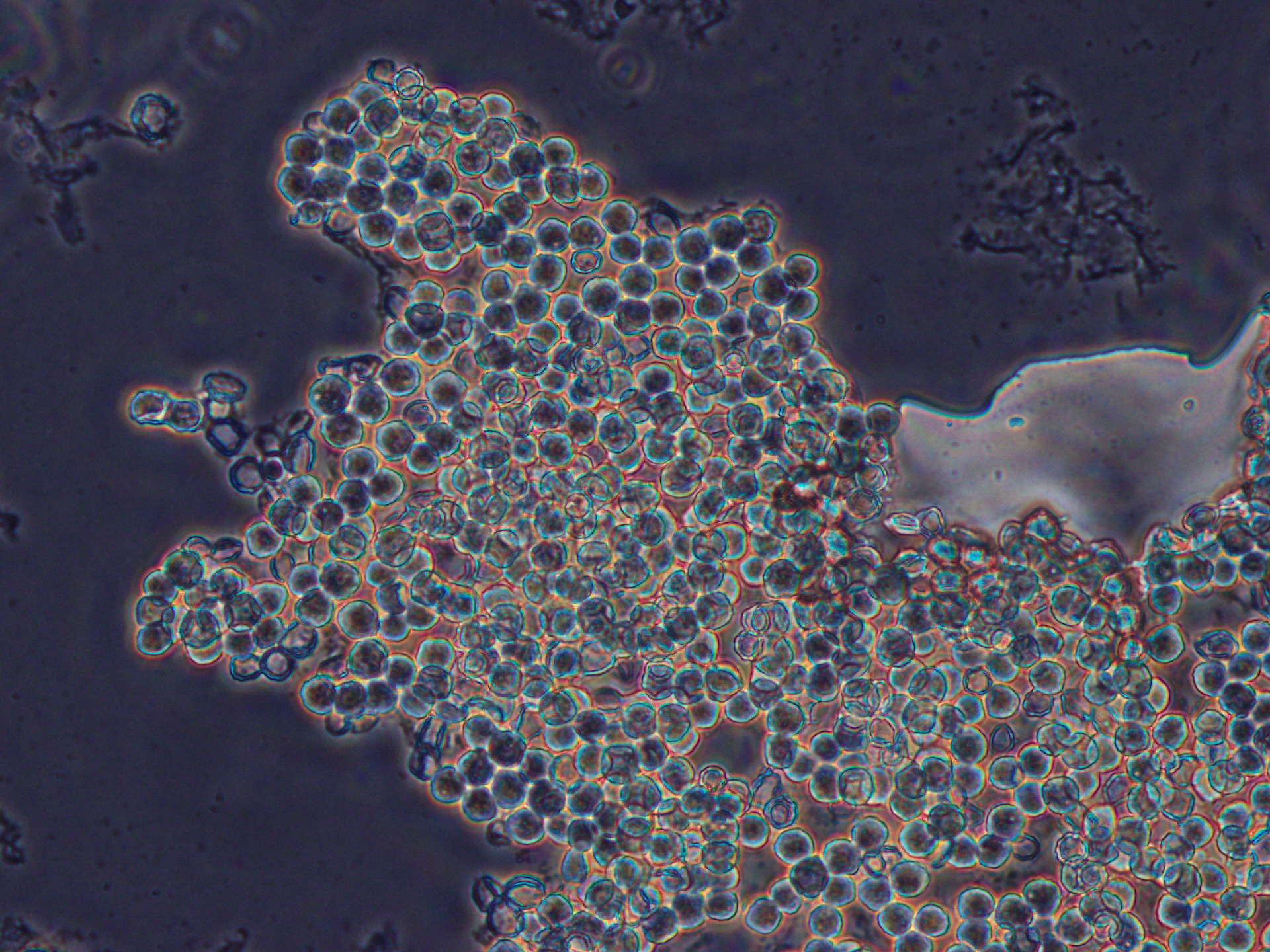

Supplement: Supplementary file 1 — Supplementary file1 (ZIP 208058 KB) [file 11686_2025_1053_MOESM1_ESM.zip › Supplementary_Figure3_4_5_MicroscopyImages/Cyst-16.JPG]

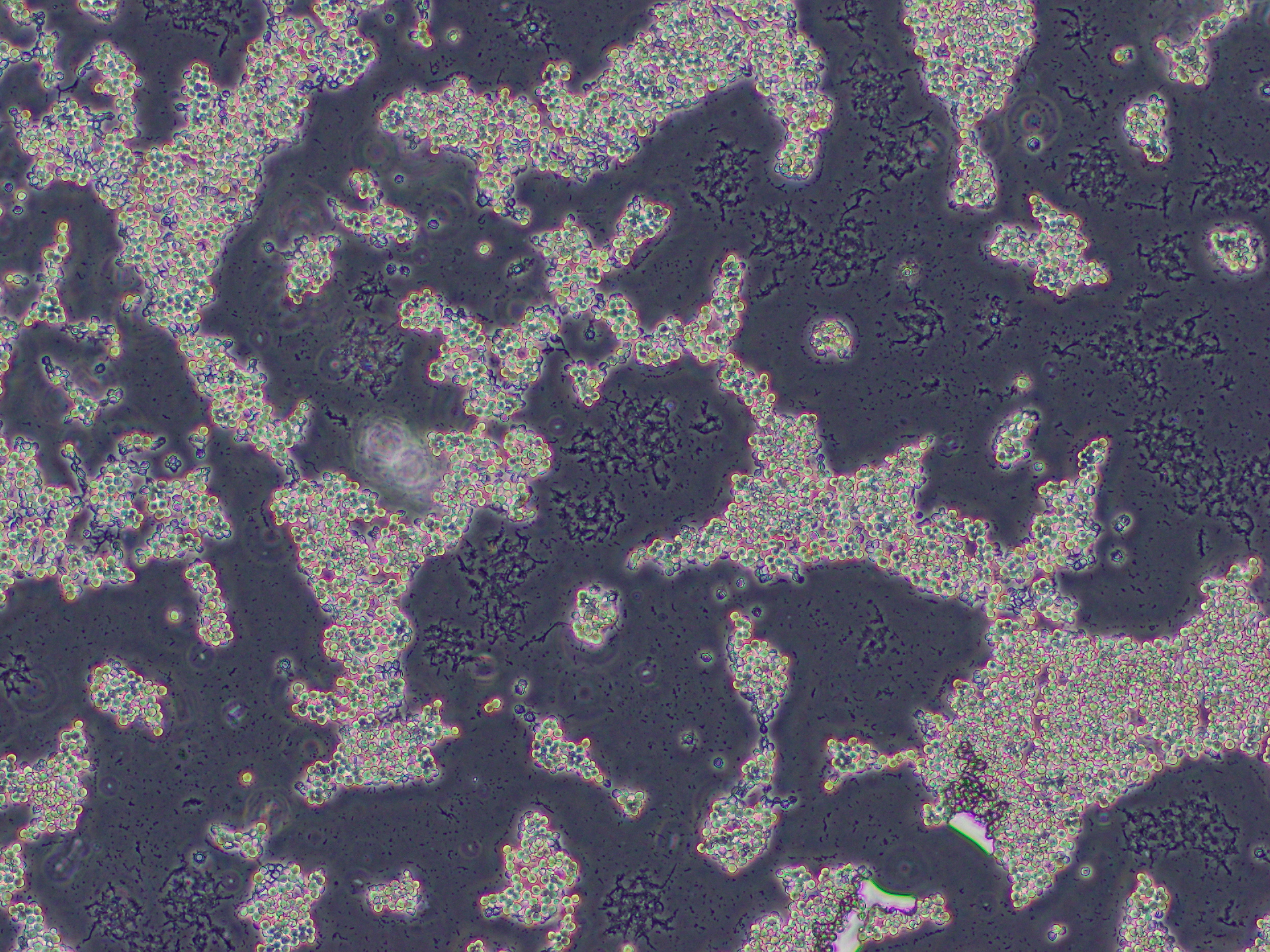

Supplement: Supplementary file 1 — Supplementary file1 (ZIP 208058 KB) [file 11686_2025_1053_MOESM1_ESM.zip › Supplementary_Figure3_4_5_MicroscopyImages/Cyst-17.JPG]

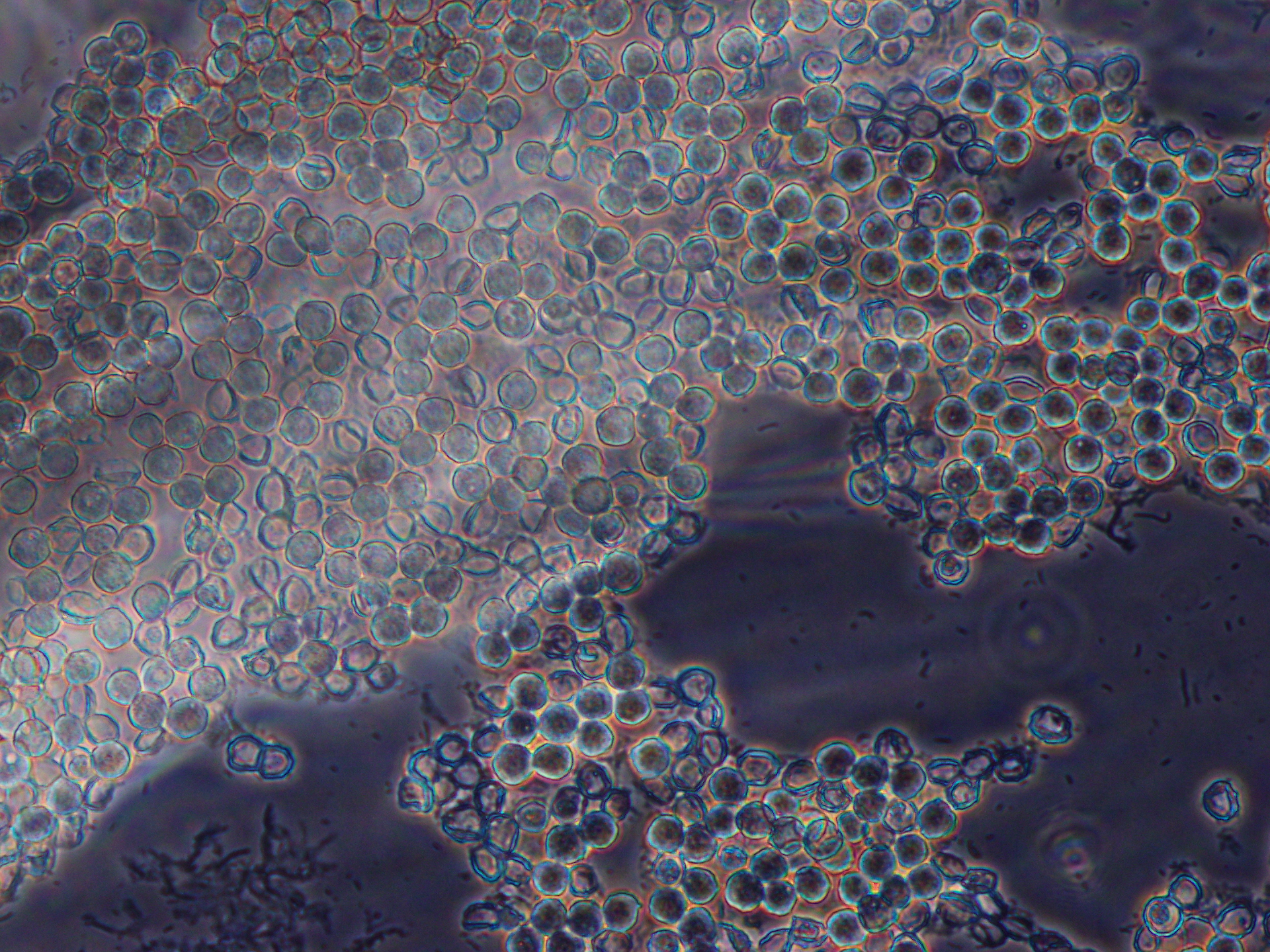

Supplement: Supplementary file 1 — Supplementary file1 (ZIP 208058 KB) [file 11686_2025_1053_MOESM1_ESM.zip › Supplementary_Figure3_4_5_MicroscopyImages/Cyst-18.JPG]

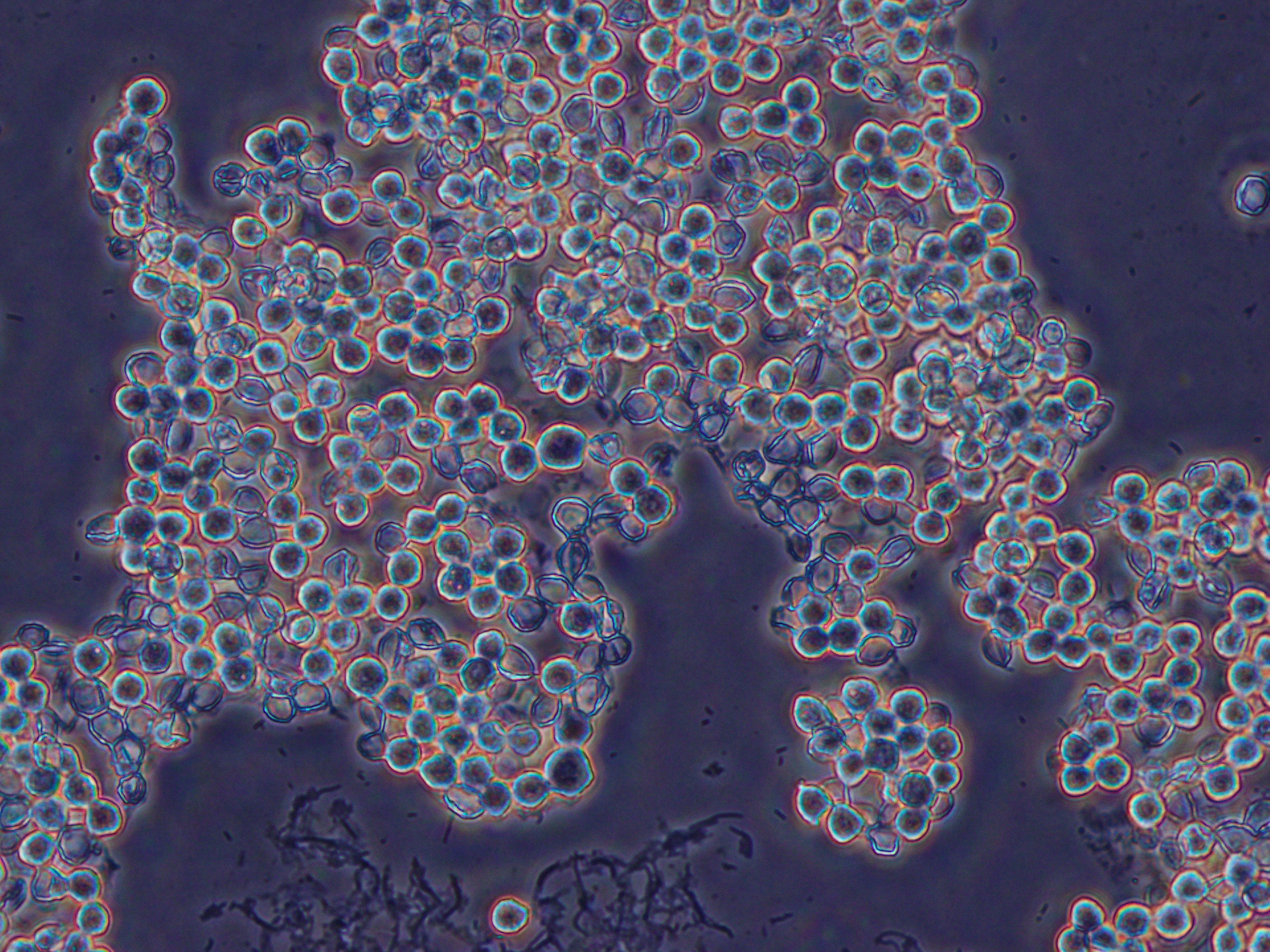

Supplement: Supplementary file 1 — Supplementary file1 (ZIP 208058 KB) [file 11686_2025_1053_MOESM1_ESM.zip › Supplementary_Figure3_4_5_MicroscopyImages/Cyst-19.JPG]

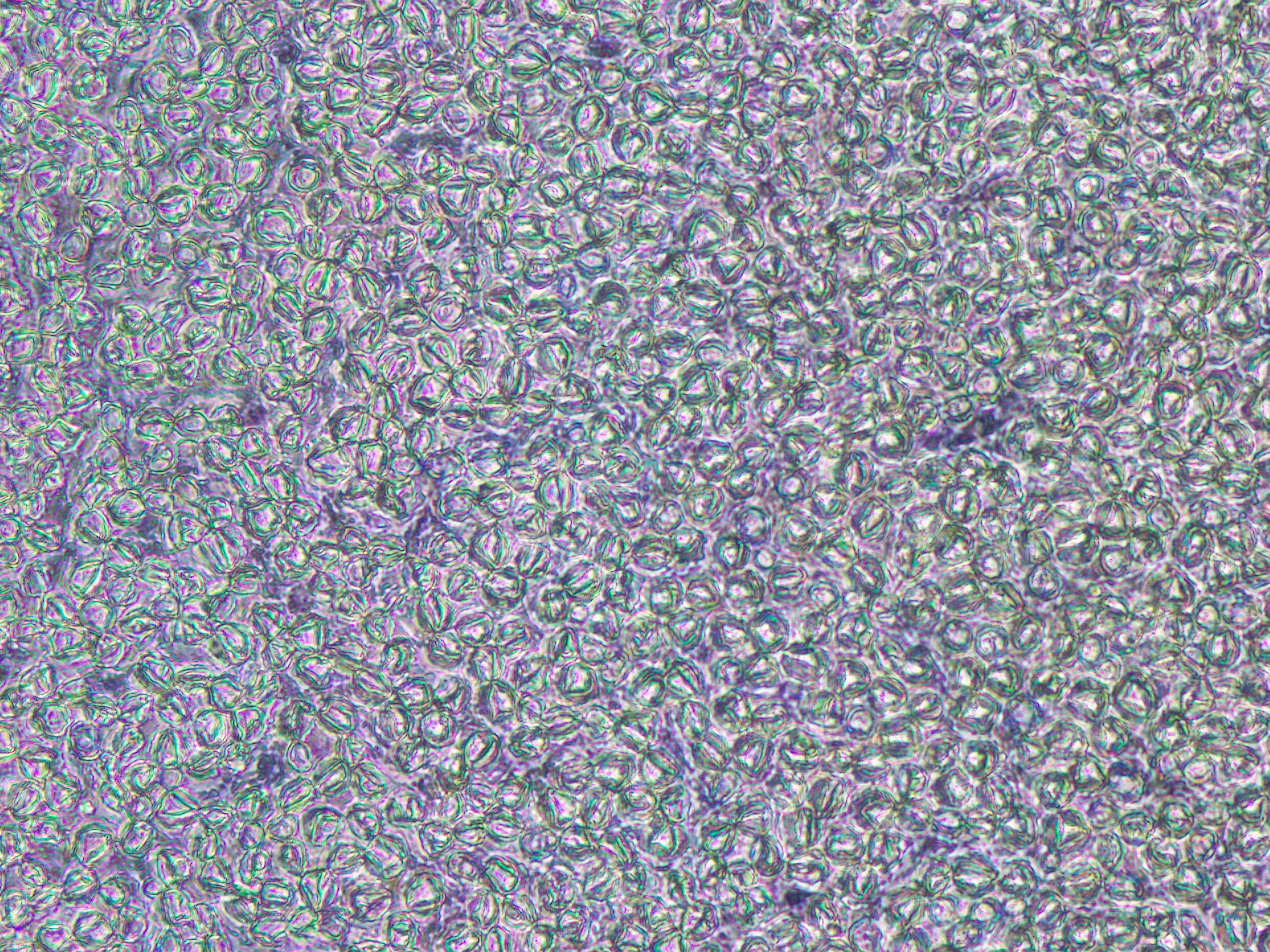

Supplement: Supplementary file 1 — Supplementary file1 (ZIP 208058 KB) [file 11686_2025_1053_MOESM1_ESM.zip › Supplementary_Figure3_4_5_MicroscopyImages/Cyst-2.JPG]

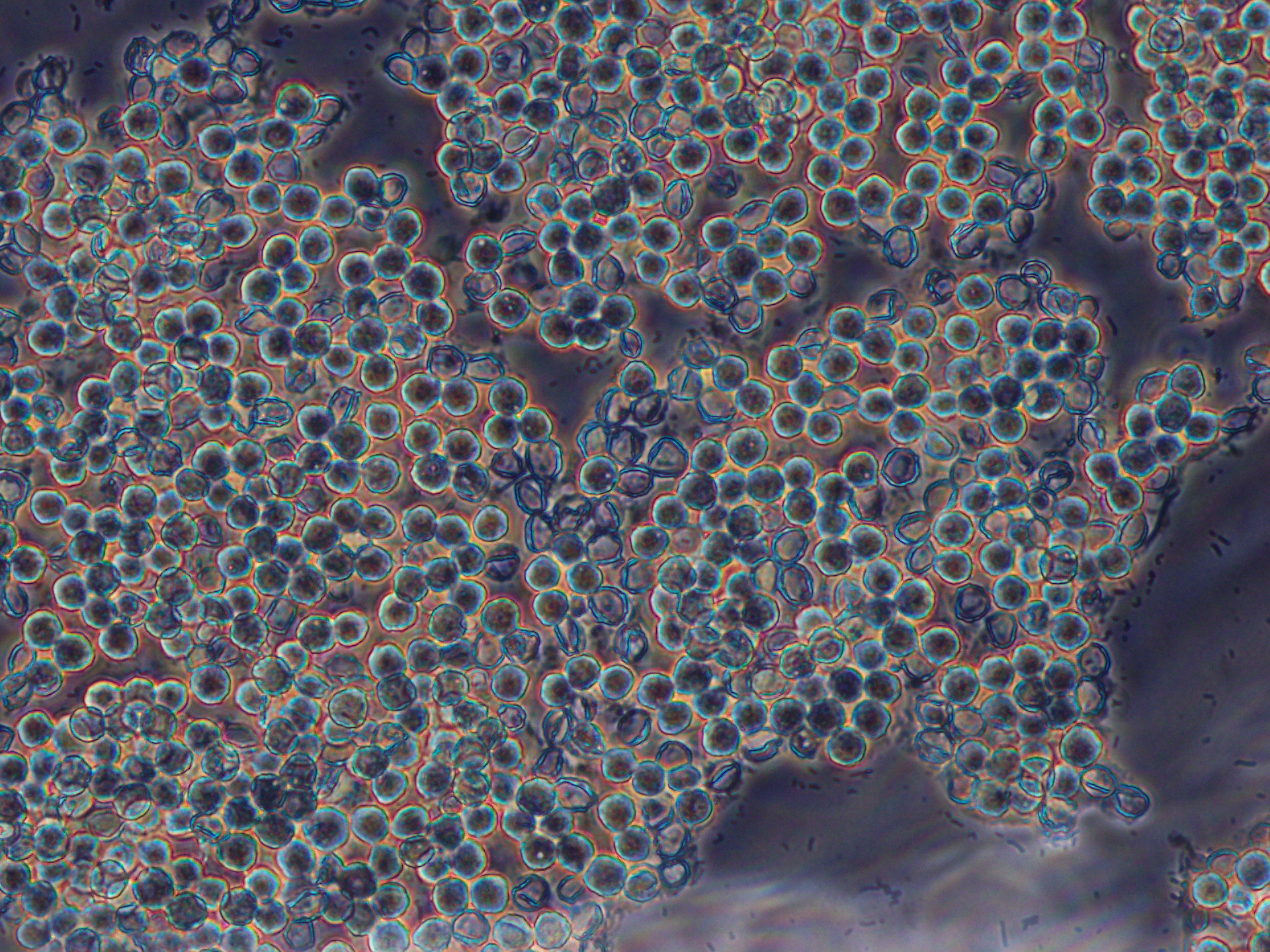

Supplement: Supplementary file 1 — Supplementary file1 (ZIP 208058 KB) [file 11686_2025_1053_MOESM1_ESM.zip › Supplementary_Figure3_4_5_MicroscopyImages/Cyst-20.JPG]

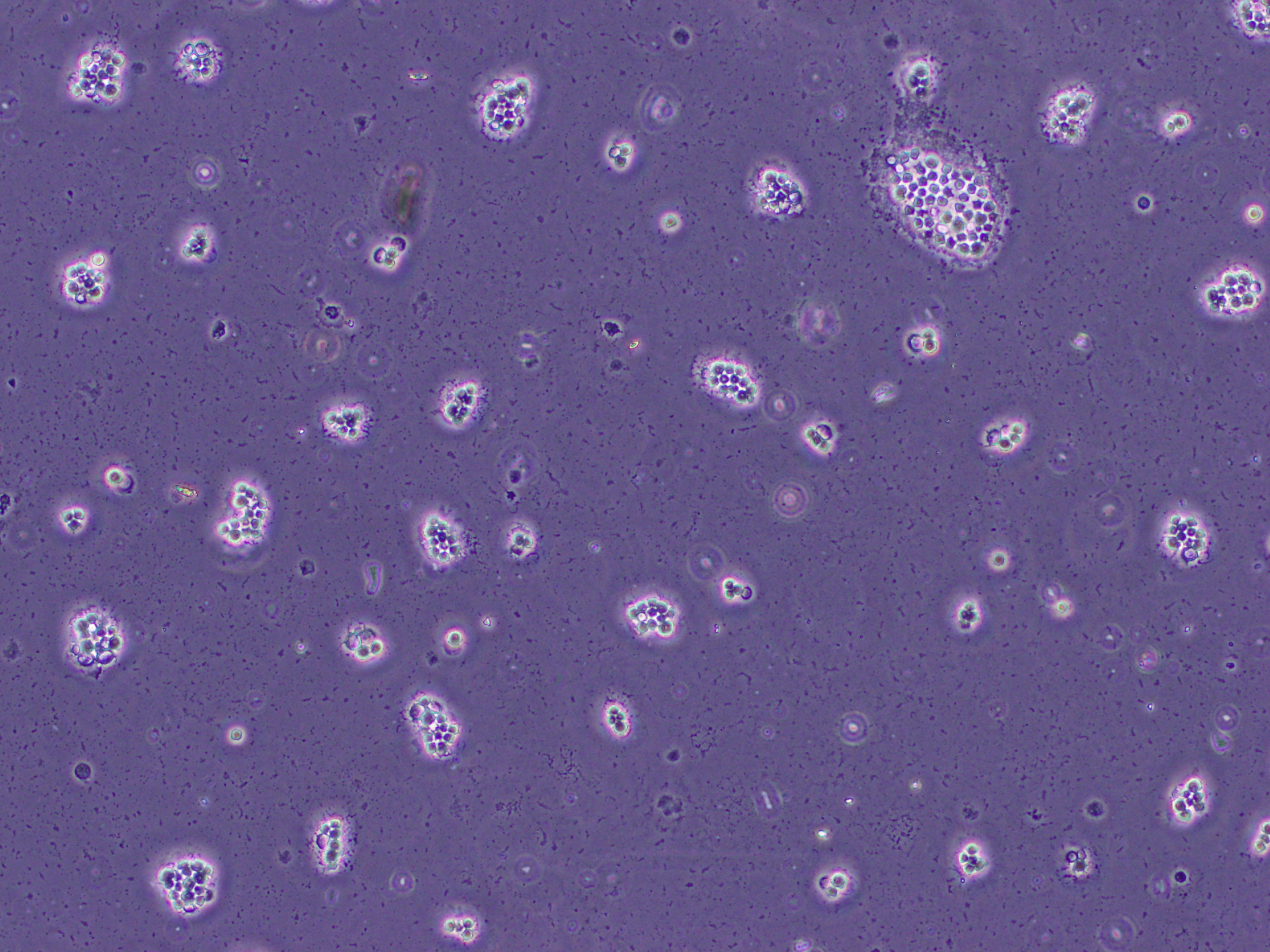

Supplement: Supplementary file 1 — Supplementary file1 (ZIP 208058 KB) [file 11686_2025_1053_MOESM1_ESM.zip › Supplementary_Figure3_4_5_MicroscopyImages/Cyst-21.JPG]

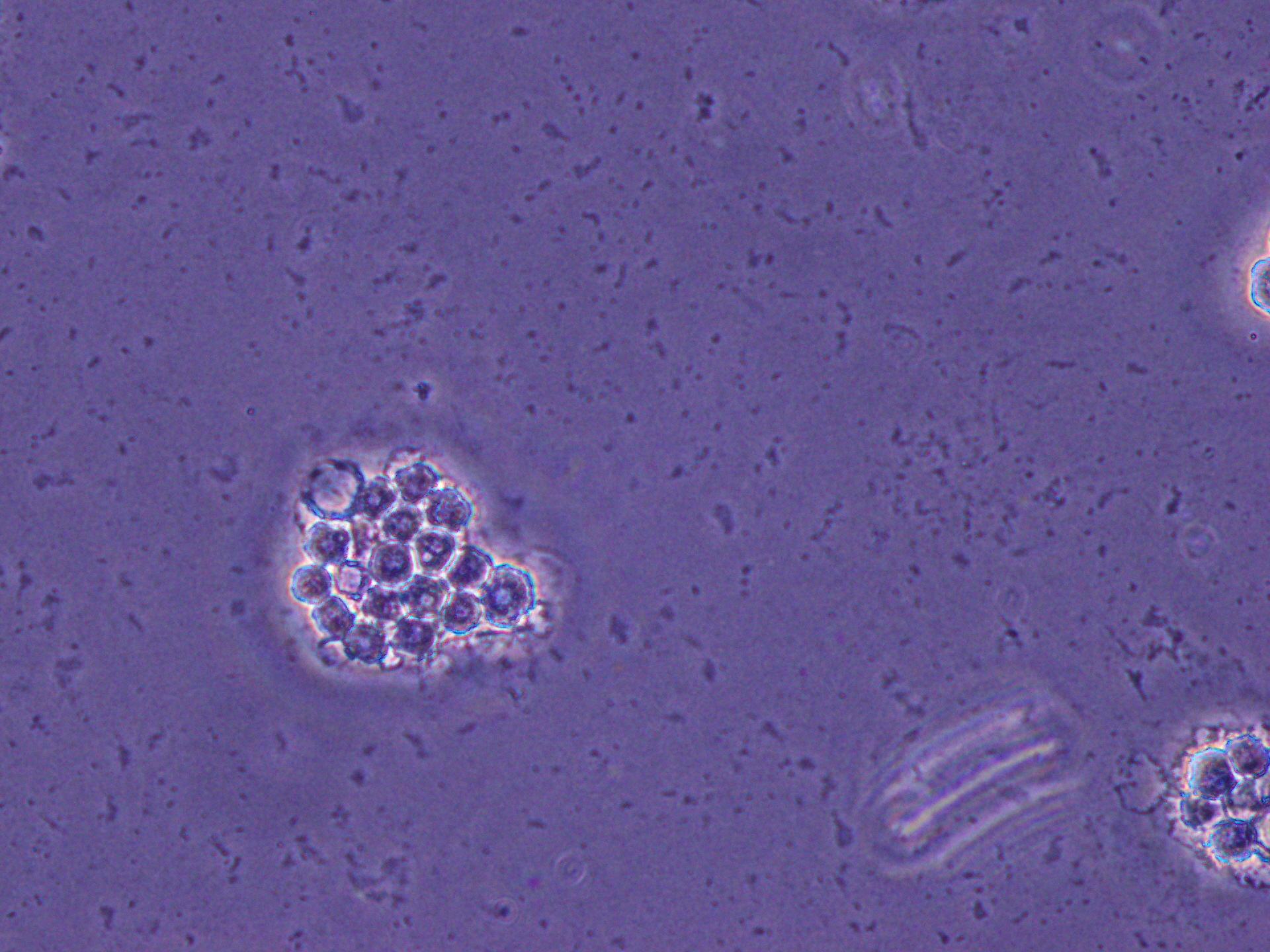

Supplement: Supplementary file 1 — Supplementary file1 (ZIP 208058 KB) [file 11686_2025_1053_MOESM1_ESM.zip › Supplementary_Figure3_4_5_MicroscopyImages/Cyst-22.JPG]

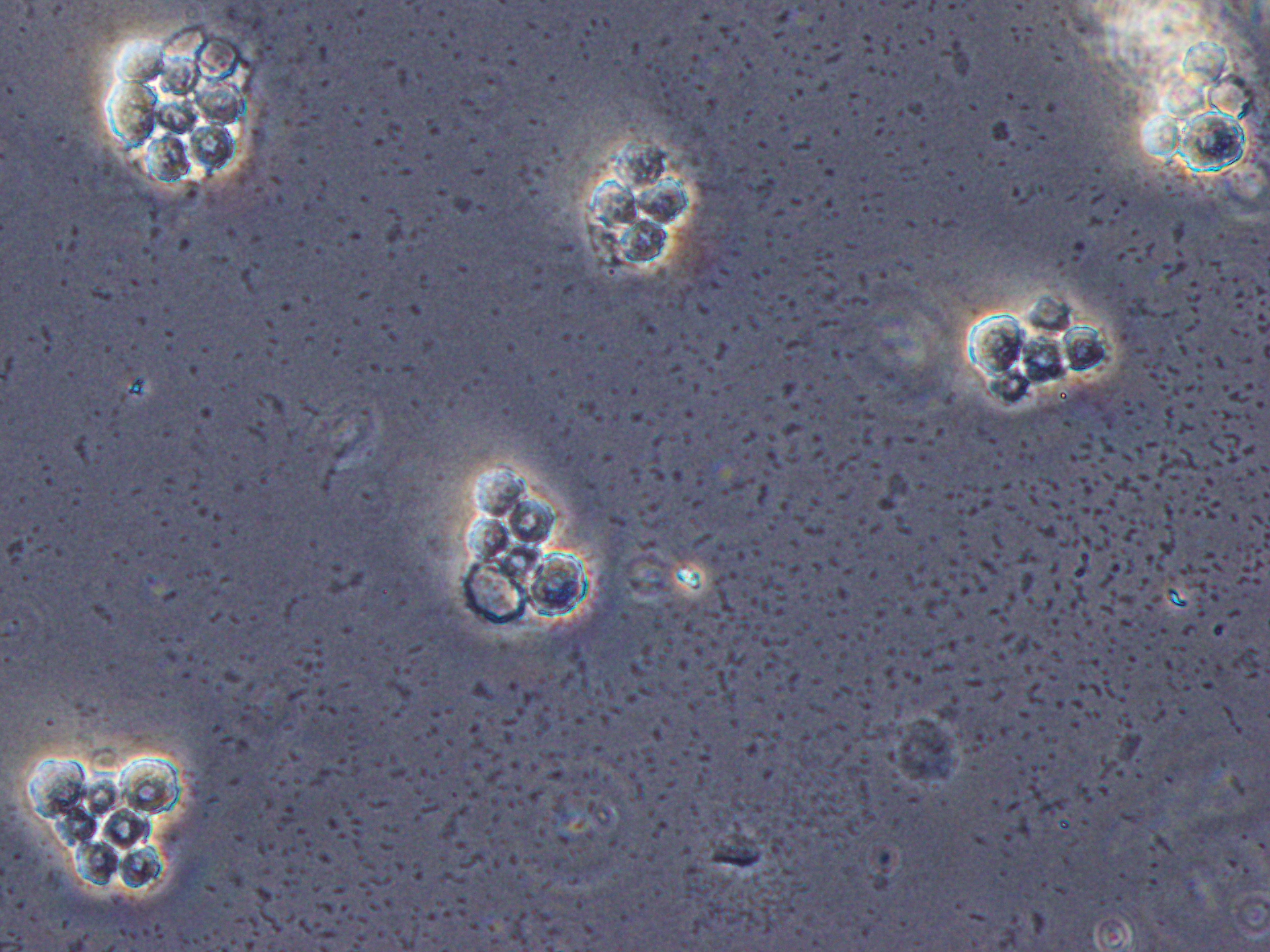

Supplement: Supplementary file 1 — Supplementary file1 (ZIP 208058 KB) [file 11686_2025_1053_MOESM1_ESM.zip › Supplementary_Figure3_4_5_MicroscopyImages/Cyst-23.JPG]

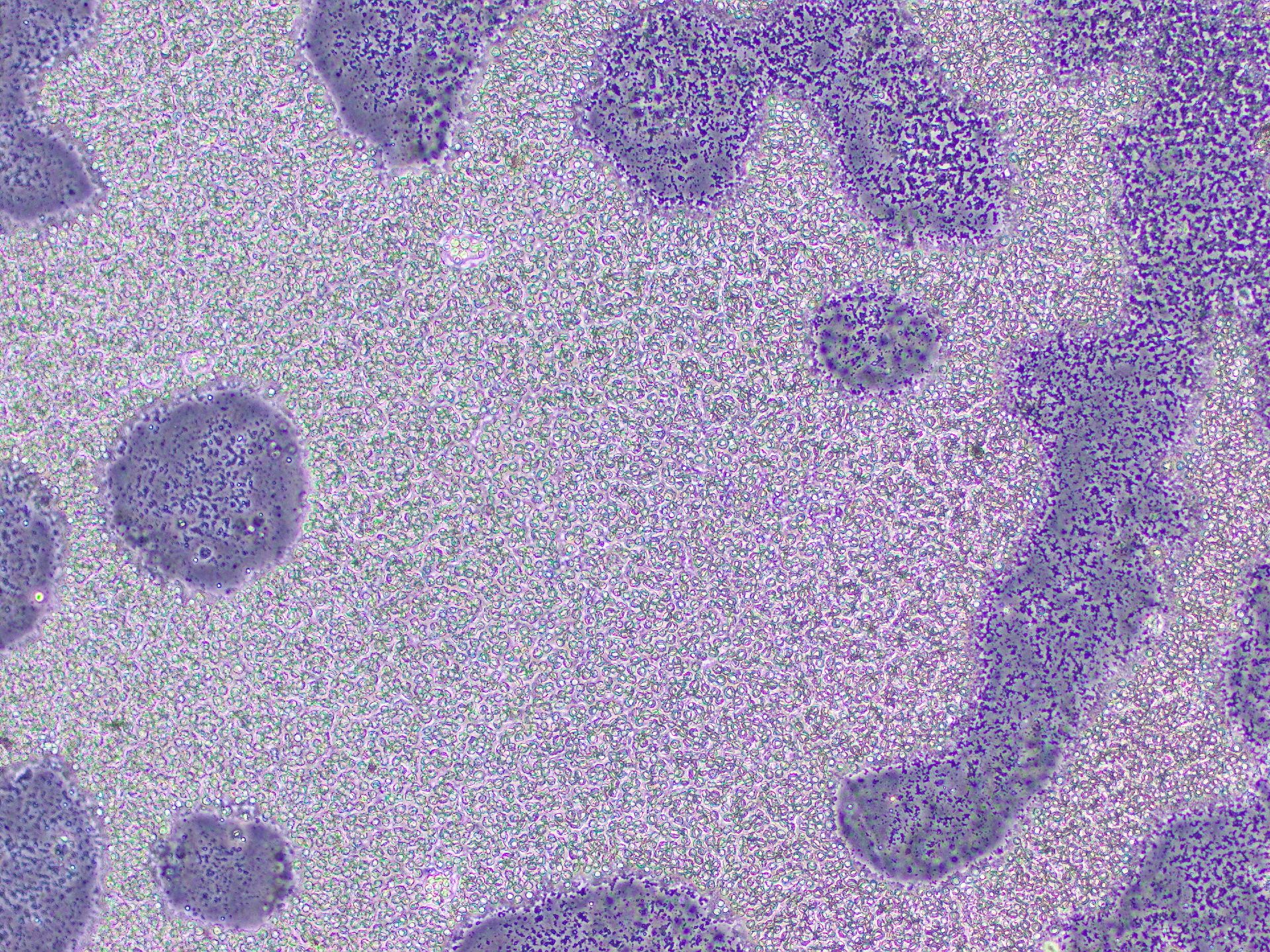

Supplement: Supplementary file 1 — Supplementary file1 (ZIP 208058 KB) [file 11686_2025_1053_MOESM1_ESM.zip › Supplementary_Figure3_4_5_MicroscopyImages/Cyst-24.JPG]

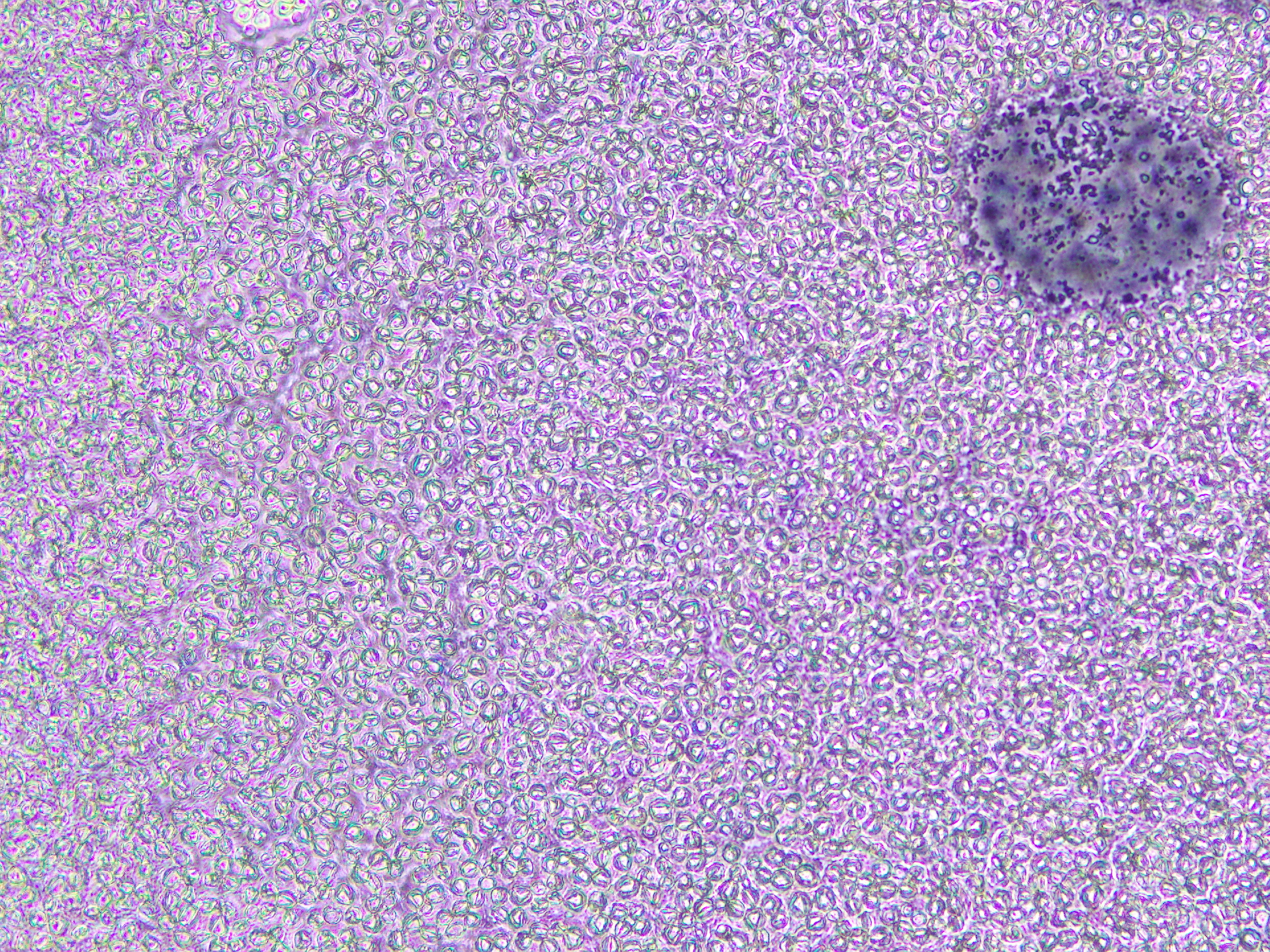

Supplement: Supplementary file 1 — Supplementary file1 (ZIP 208058 KB) [file 11686_2025_1053_MOESM1_ESM.zip › Supplementary_Figure3_4_5_MicroscopyImages/Cyst-25.JPG]

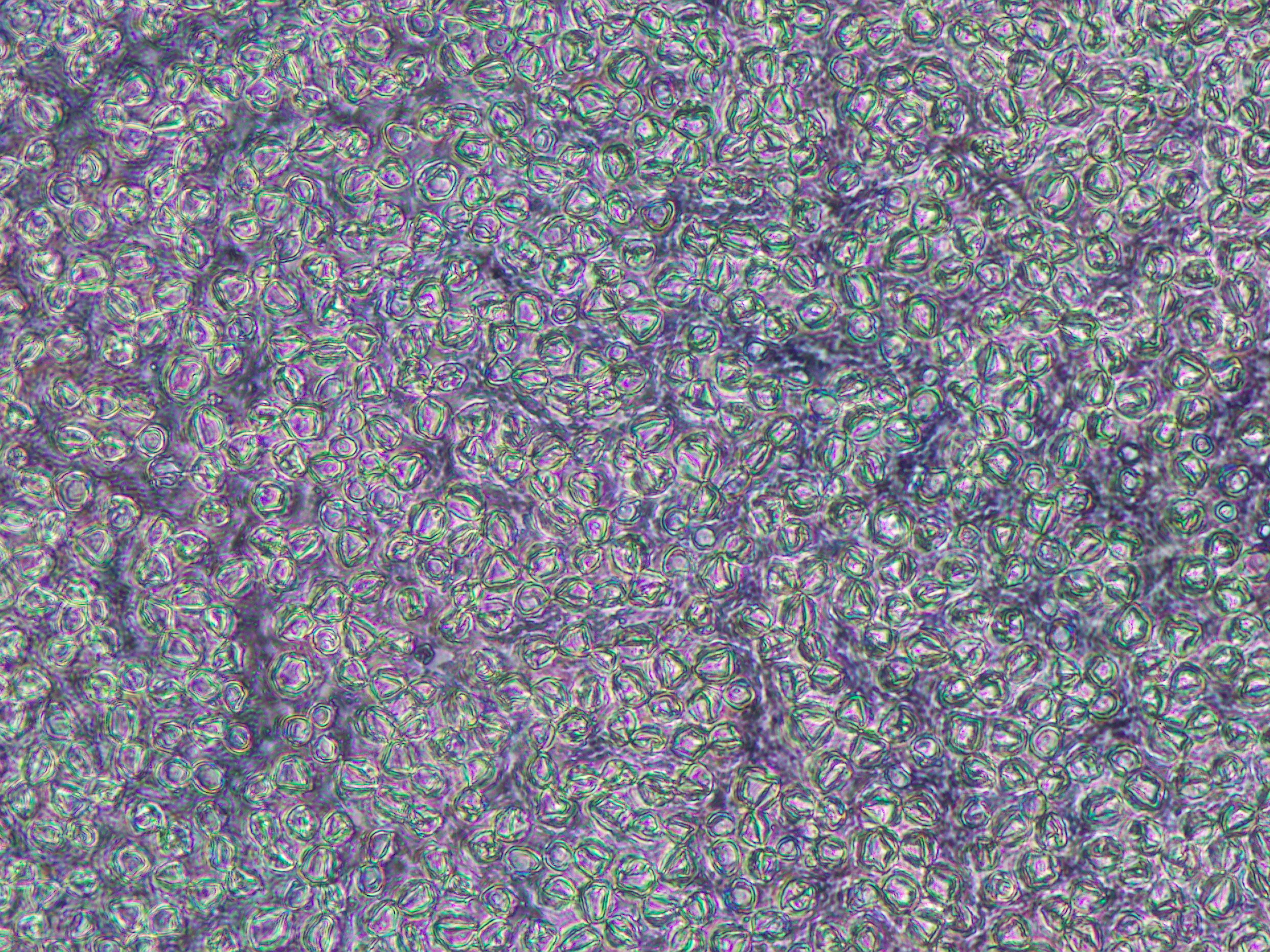

Supplement: Supplementary file 1 — Supplementary file1 (ZIP 208058 KB) [file 11686_2025_1053_MOESM1_ESM.zip › Supplementary_Figure3_4_5_MicroscopyImages/Cyst-26.JPG]

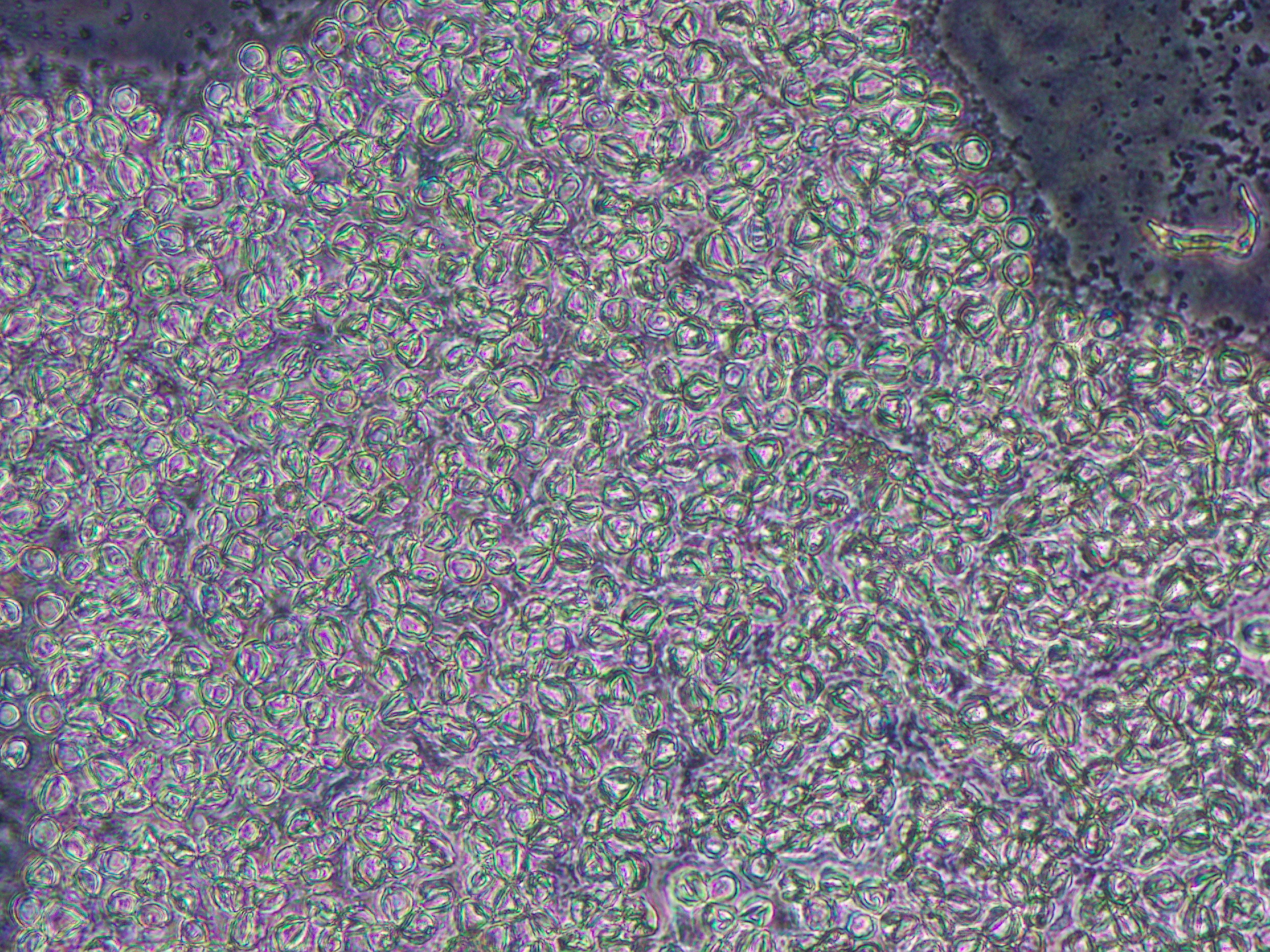

Supplement: Supplementary file 1 — Supplementary file1 (ZIP 208058 KB) [file 11686_2025_1053_MOESM1_ESM.zip › Supplementary_Figure3_4_5_MicroscopyImages/Cyst-27.JPG]

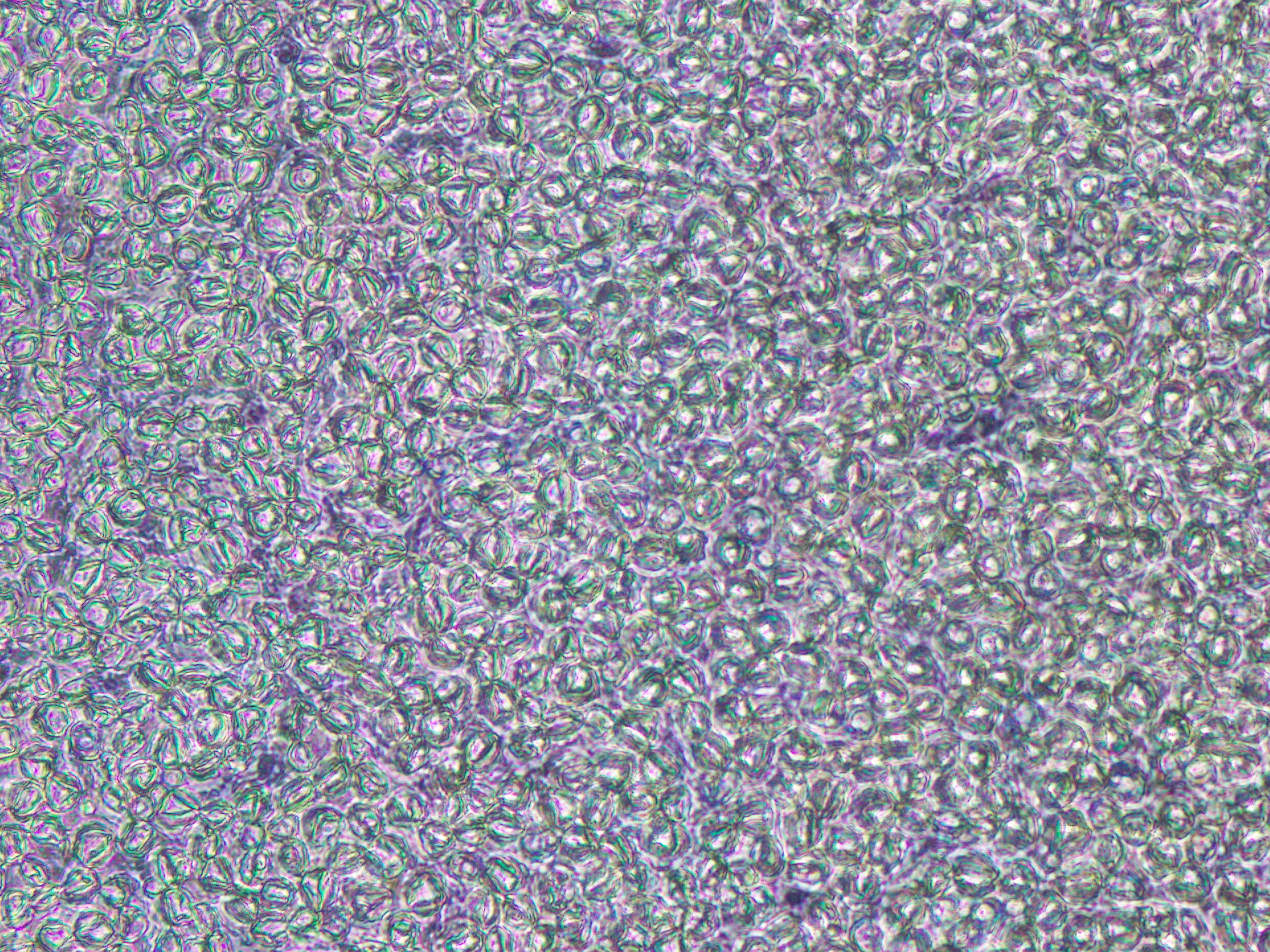

Supplement: Supplementary file 1 — Supplementary file1 (ZIP 208058 KB) [file 11686_2025_1053_MOESM1_ESM.zip › Supplementary_Figure3_4_5_MicroscopyImages/Cyst-28.JPG]

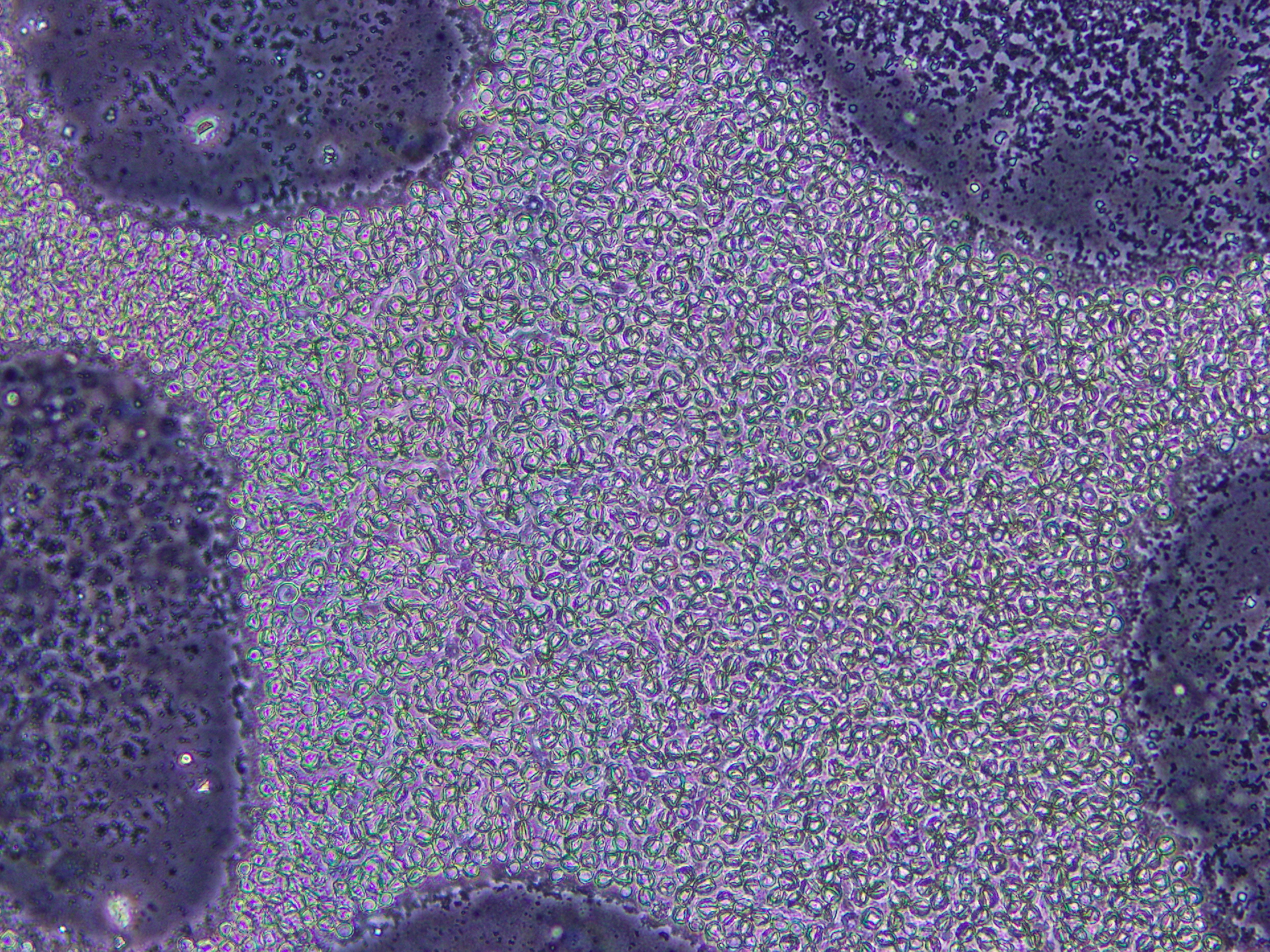

Supplement: Supplementary file 1 — Supplementary file1 (ZIP 208058 KB) [file 11686_2025_1053_MOESM1_ESM.zip › Supplementary_Figure3_4_5_MicroscopyImages/Cyst-29.JPG]

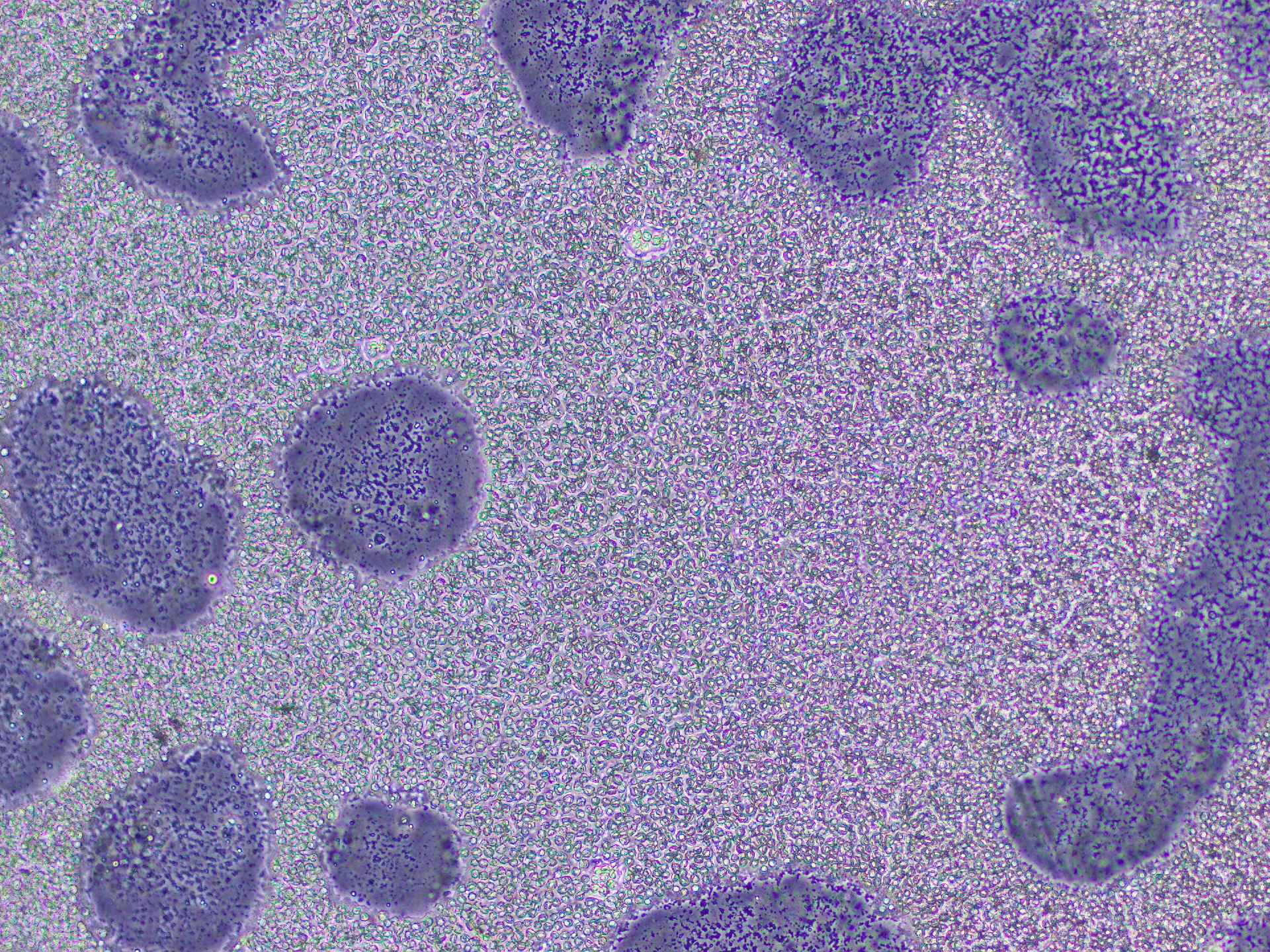

Supplement: Supplementary file 1 — Supplementary file1 (ZIP 208058 KB) [file 11686_2025_1053_MOESM1_ESM.zip › Supplementary_Figure3_4_5_MicroscopyImages/Cyst-3.JPG]

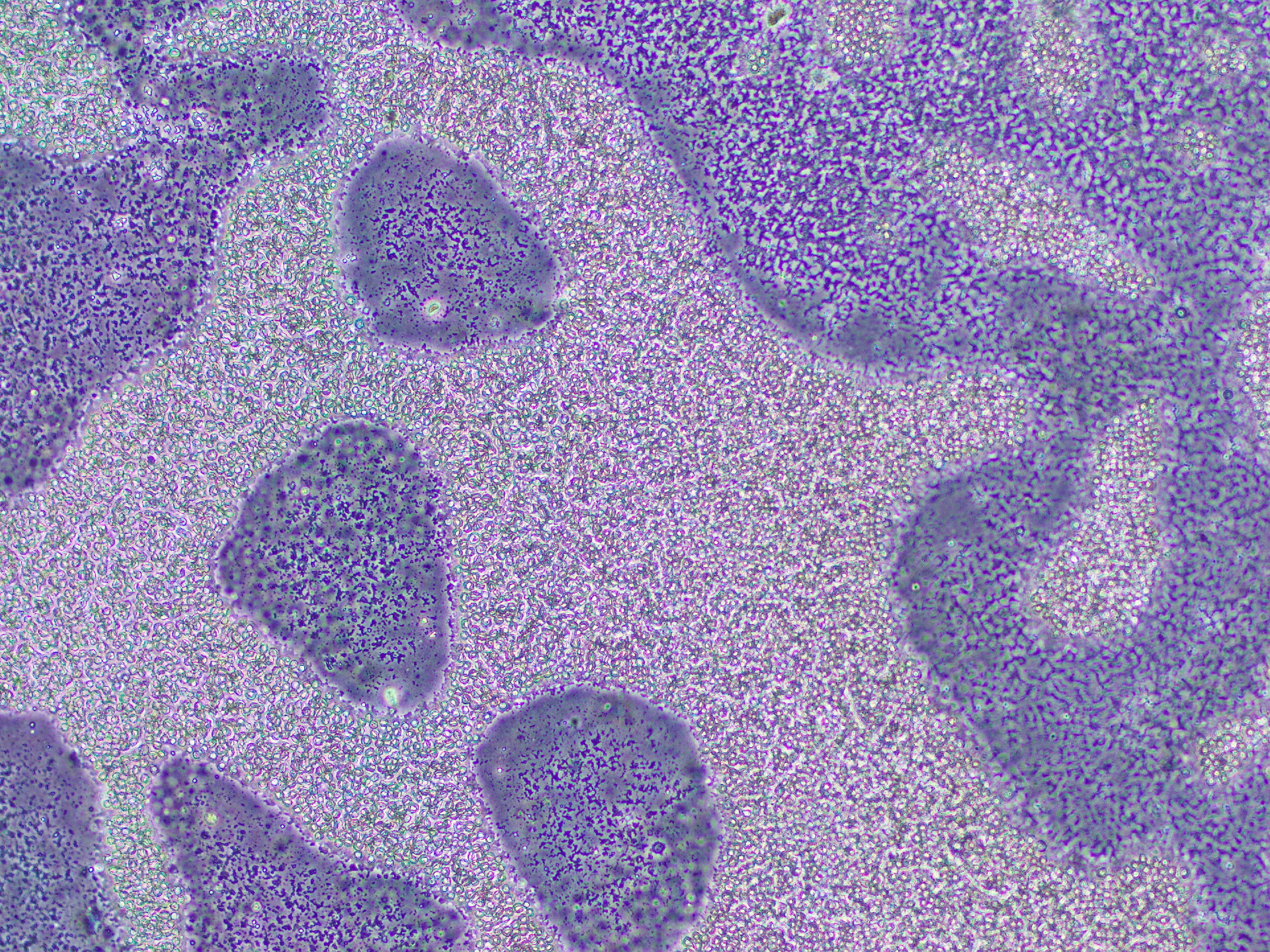

Supplement: Supplementary file 1 — Supplementary file1 (ZIP 208058 KB) [file 11686_2025_1053_MOESM1_ESM.zip › Supplementary_Figure3_4_5_MicroscopyImages/Cyst-30.JPG]

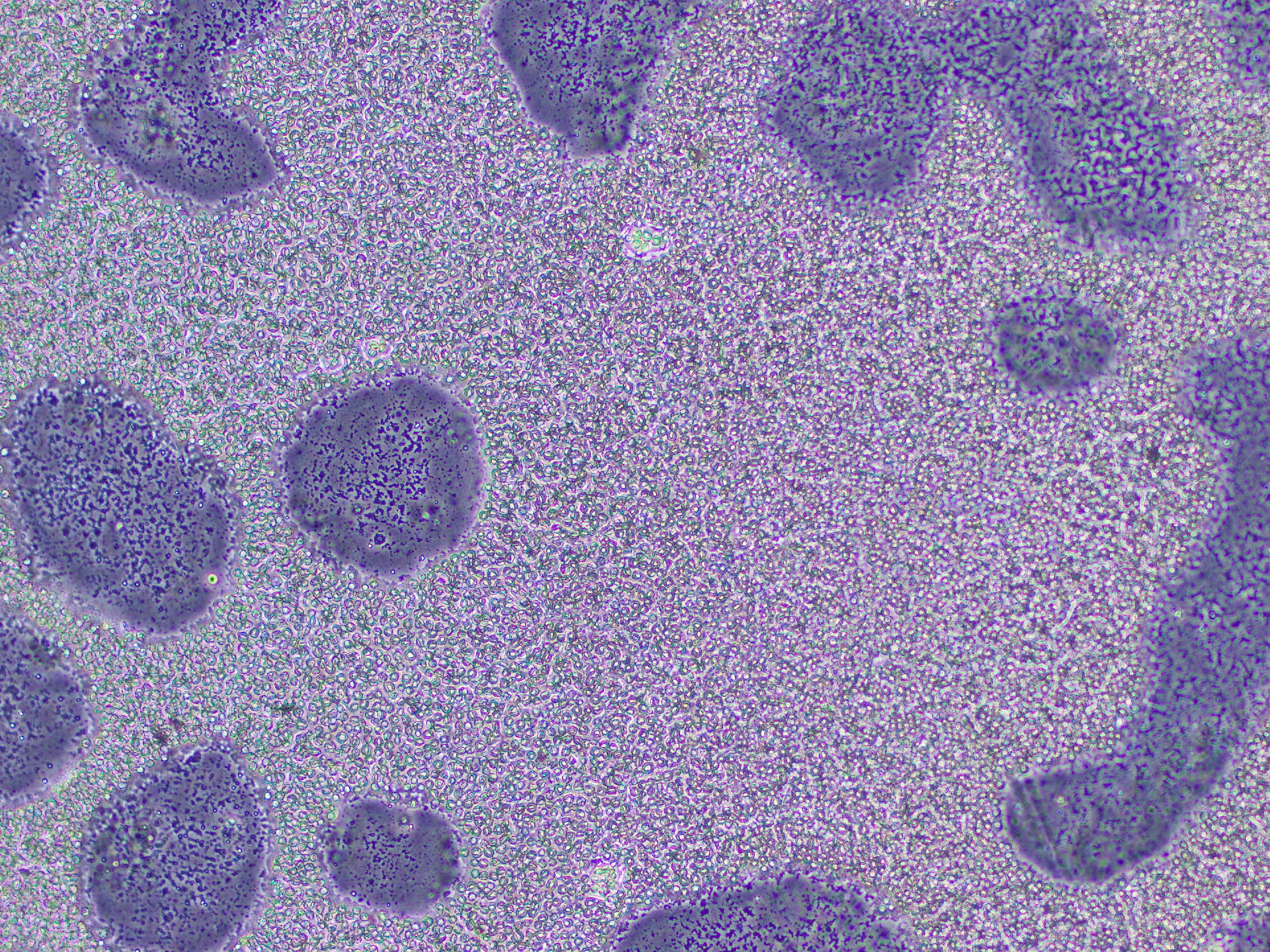

Supplement: Supplementary file 1 — Supplementary file1 (ZIP 208058 KB) [file 11686_2025_1053_MOESM1_ESM.zip › Supplementary_Figure3_4_5_MicroscopyImages/Cyst-31.JPG]

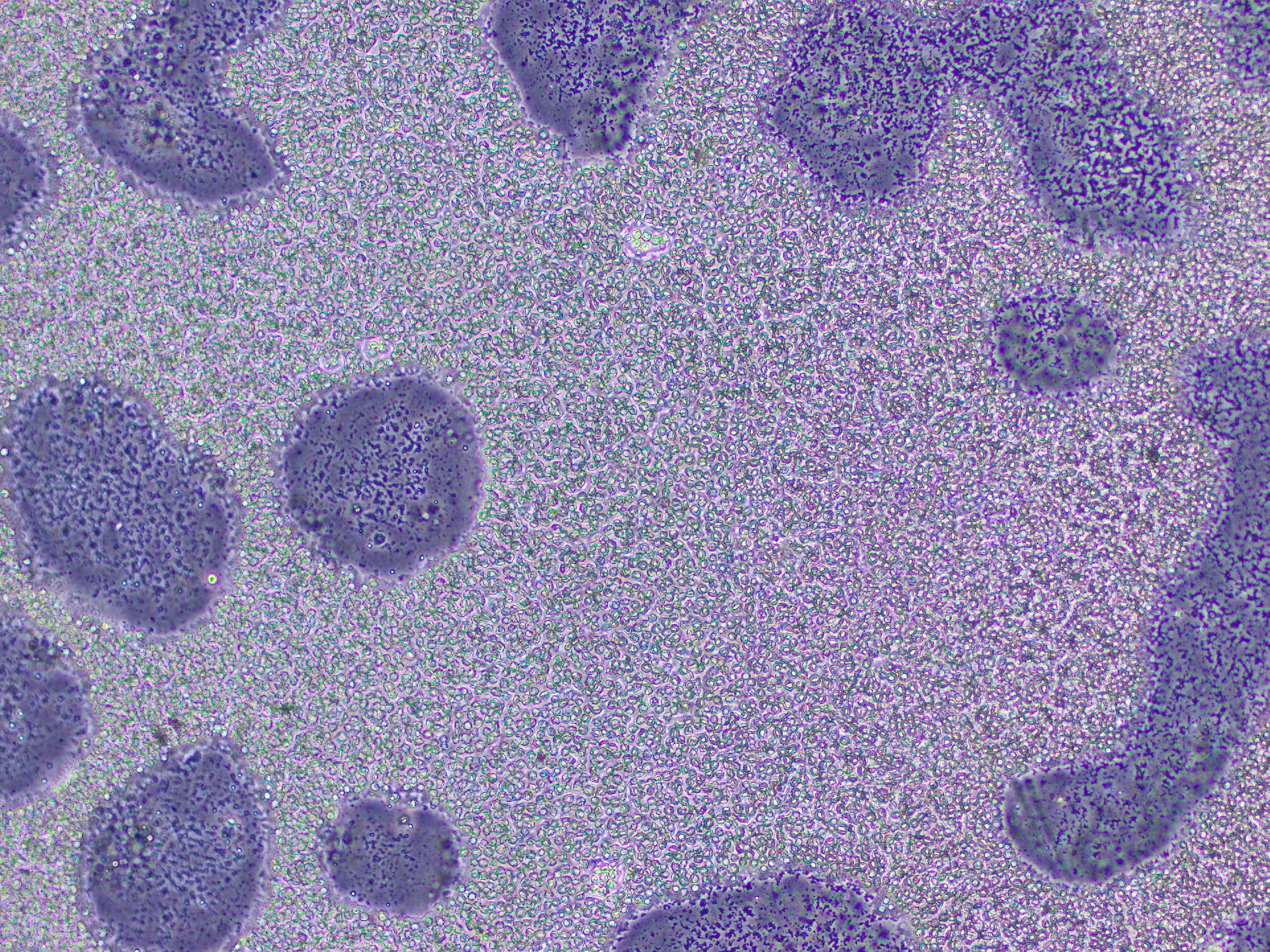

Supplement: Supplementary file 1 — Supplementary file1 (ZIP 208058 KB) [file 11686_2025_1053_MOESM1_ESM.zip › Supplementary_Figure3_4_5_MicroscopyImages/Cyst-32.JPG]

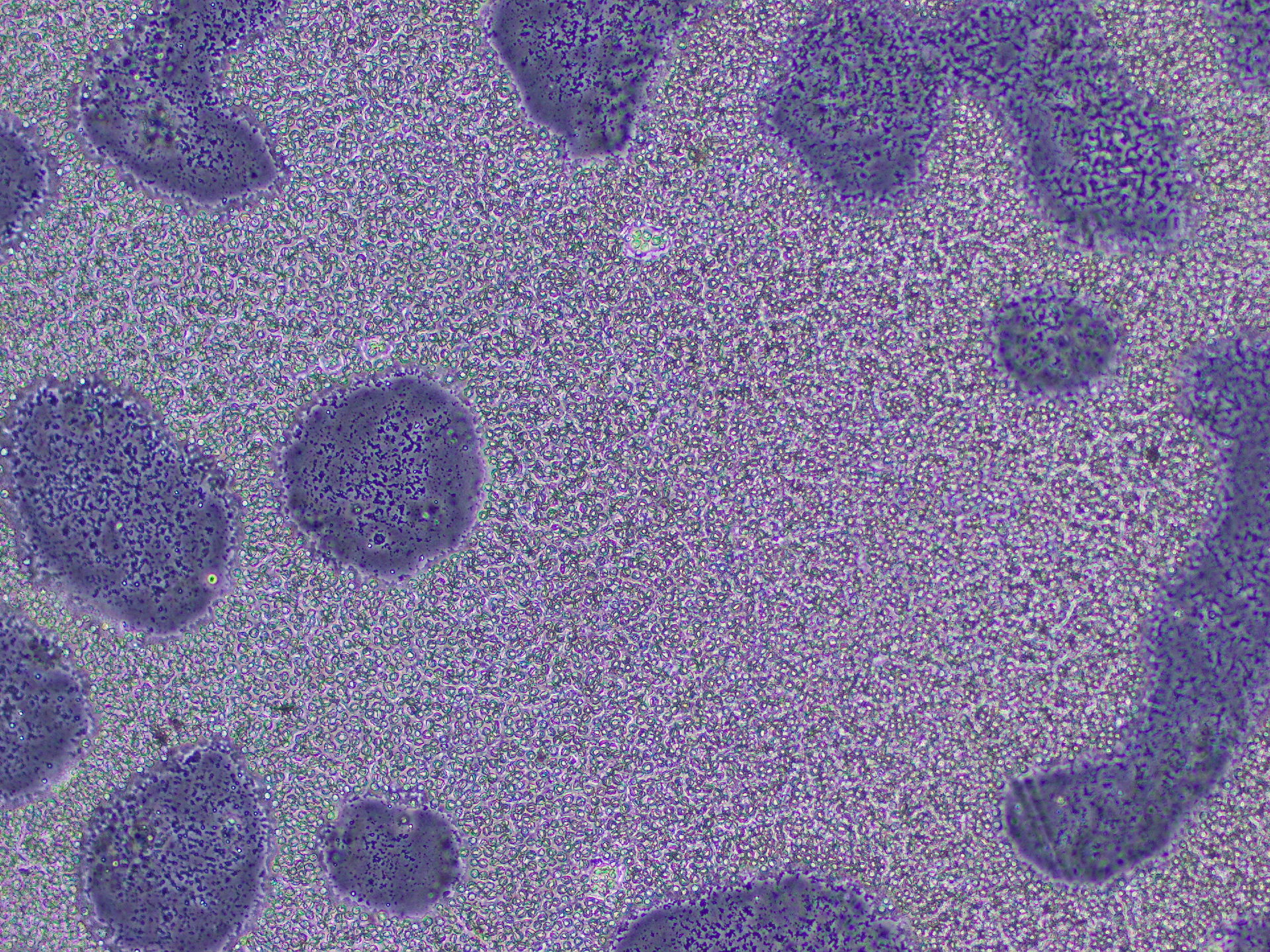

Supplement: Supplementary file 1 — Supplementary file1 (ZIP 208058 KB) [file 11686_2025_1053_MOESM1_ESM.zip › Supplementary_Figure3_4_5_MicroscopyImages/Cyst-33.JPG]

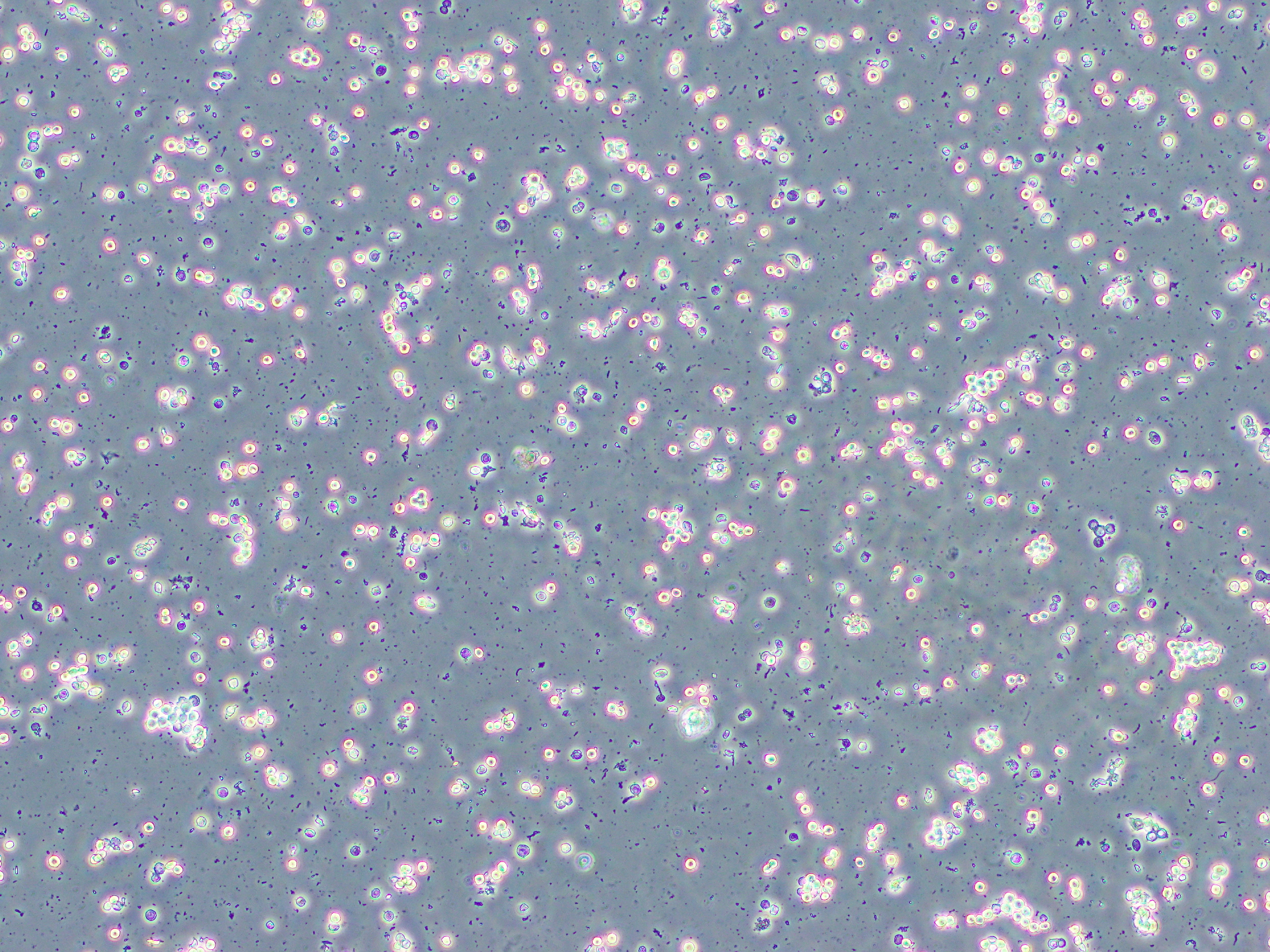

Supplement: Supplementary file 1 — Supplementary file1 (ZIP 208058 KB) [file 11686_2025_1053_MOESM1_ESM.zip › Supplementary_Figure3_4_5_MicroscopyImages/Cyst-34.JPG]

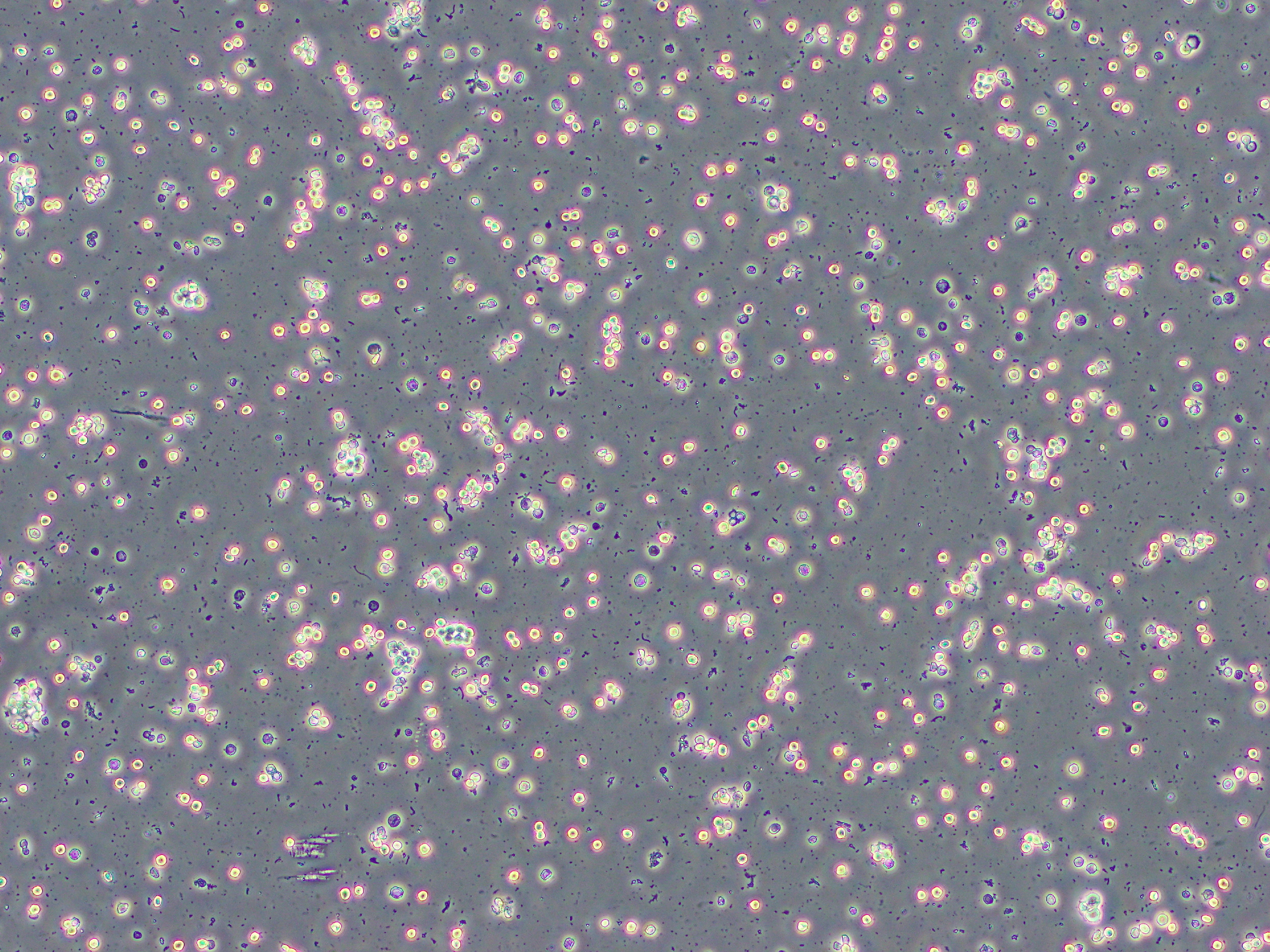

Supplement: Supplementary file 1 — Supplementary file1 (ZIP 208058 KB) [file 11686_2025_1053_MOESM1_ESM.zip › Supplementary_Figure3_4_5_MicroscopyImages/Cyst-35.JPG]

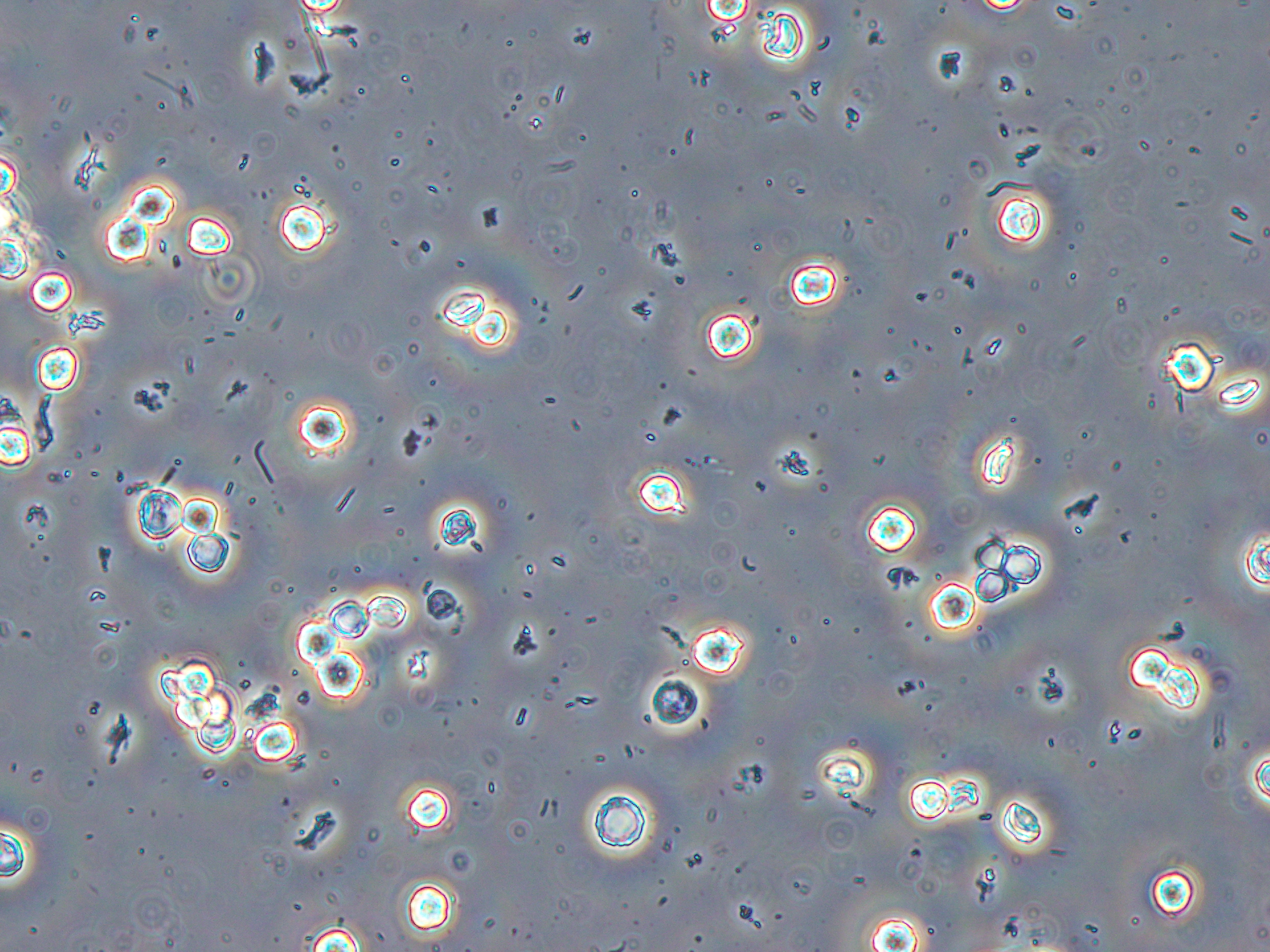

Supplement: Supplementary file 1 — Supplementary file1 (ZIP 208058 KB) [file 11686_2025_1053_MOESM1_ESM.zip › Supplementary_Figure3_4_5_MicroscopyImages/Cyst-36.JPG]

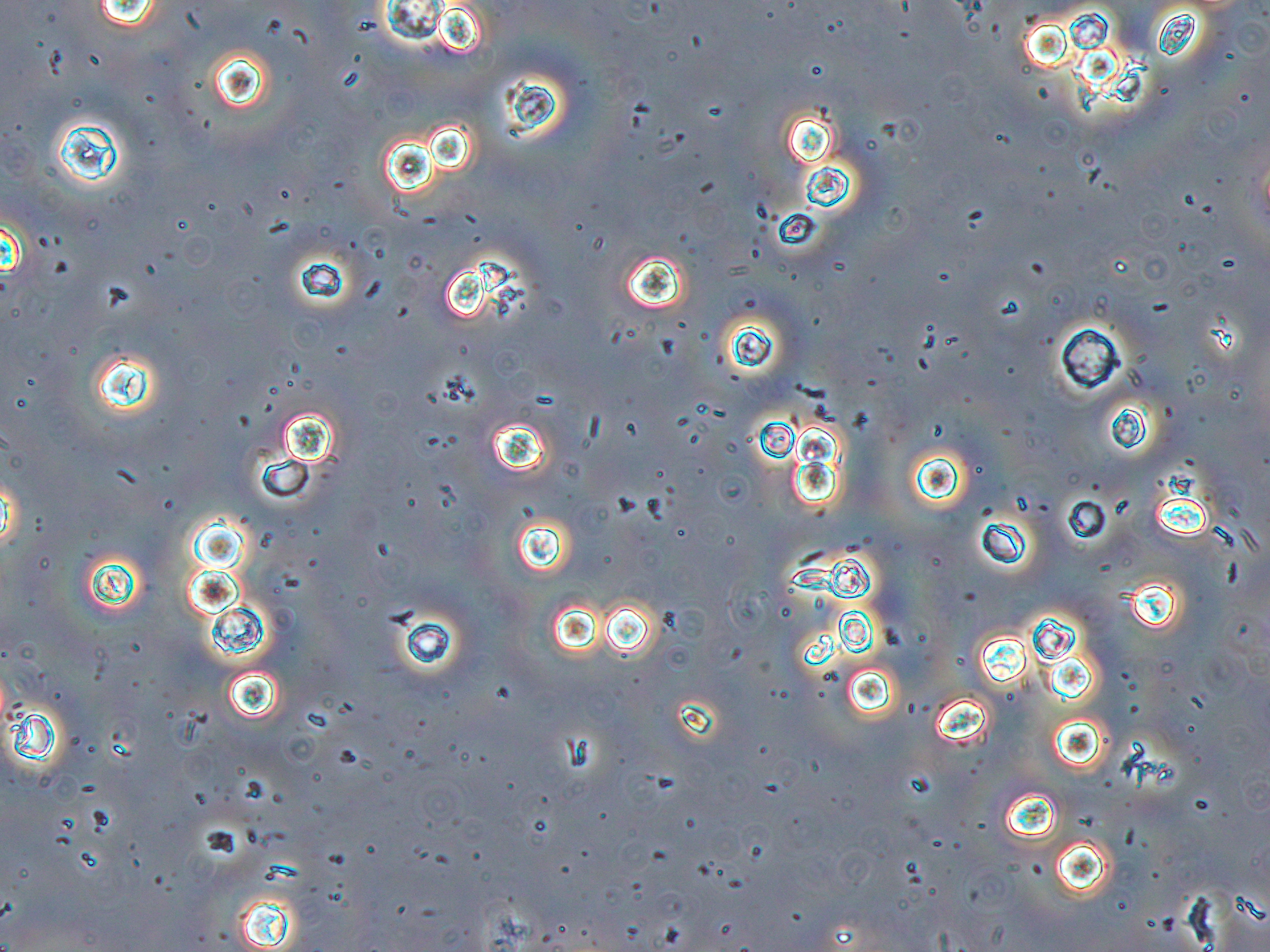

Supplement: Supplementary file 1 — Supplementary file1 (ZIP 208058 KB) [file 11686_2025_1053_MOESM1_ESM.zip › Supplementary_Figure3_4_5_MicroscopyImages/Cyst-37.JPG]

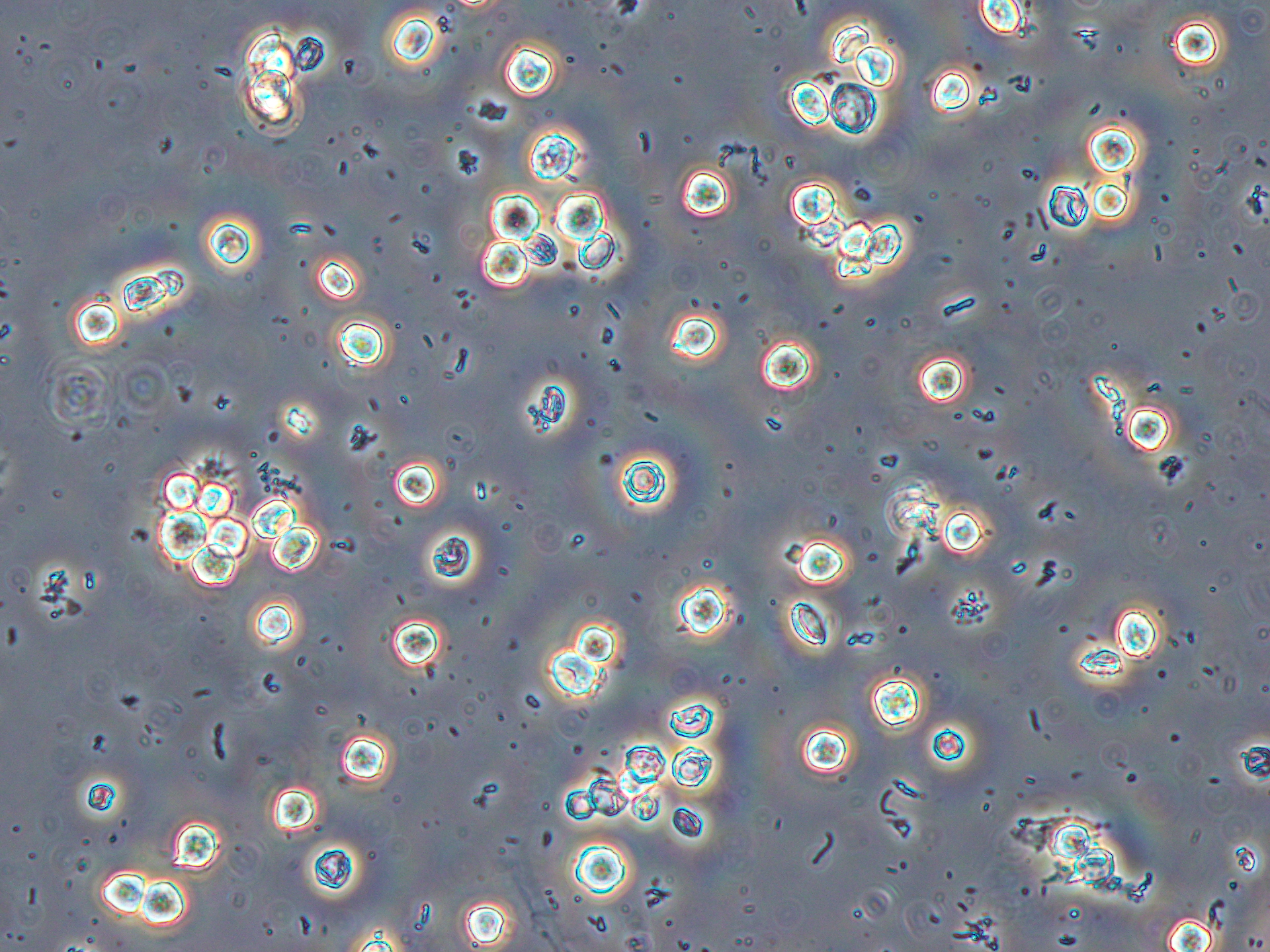

Supplement: Supplementary file 1 — Supplementary file1 (ZIP 208058 KB) [file 11686_2025_1053_MOESM1_ESM.zip › Supplementary_Figure3_4_5_MicroscopyImages/Cyst-38.JPG]

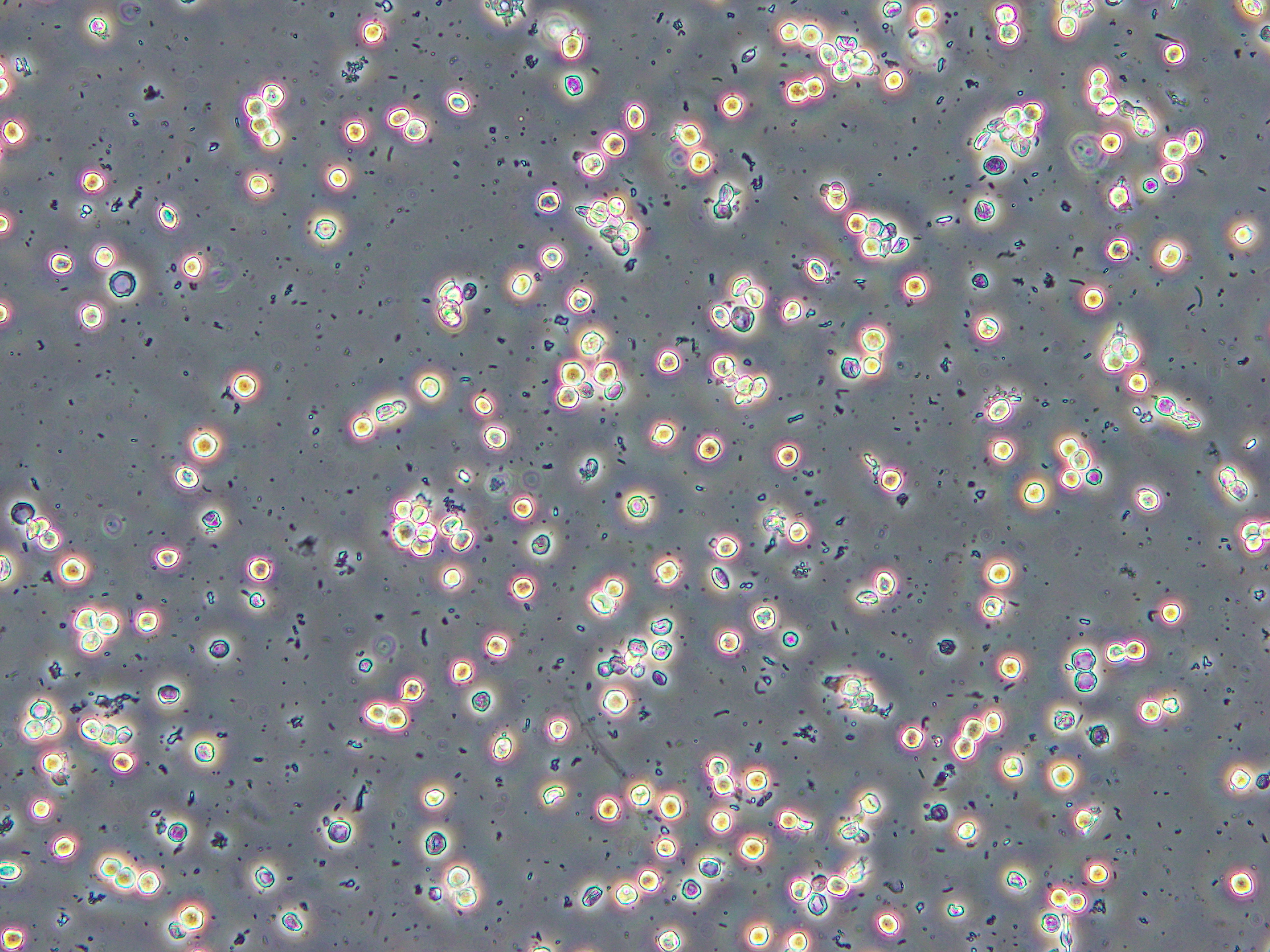

Supplement: Supplementary file 1 — Supplementary file1 (ZIP 208058 KB) [file 11686_2025_1053_MOESM1_ESM.zip › Supplementary_Figure3_4_5_MicroscopyImages/Cyst-39.JPG]

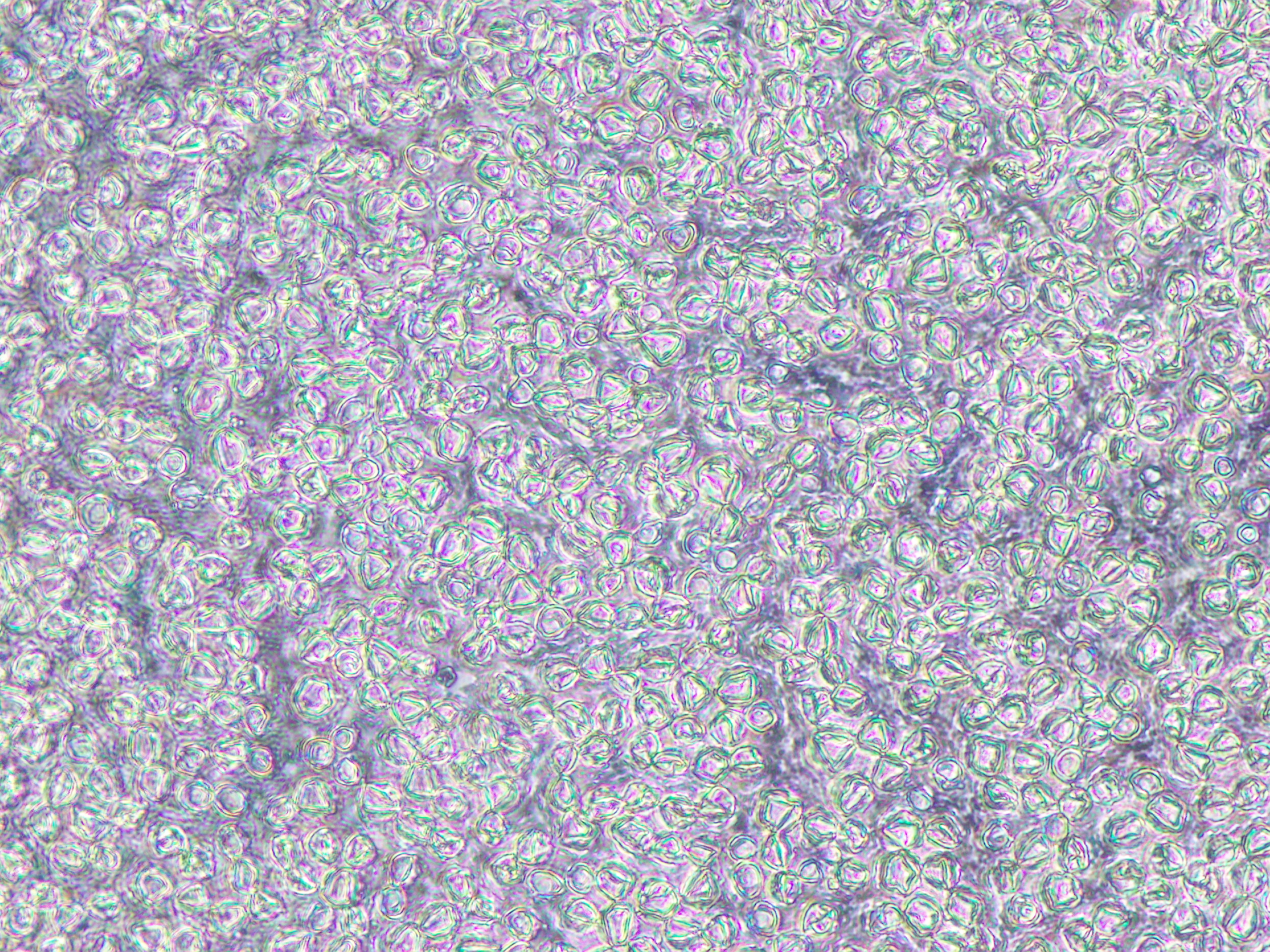

Supplement: Supplementary file 1 — Supplementary file1 (ZIP 208058 KB) [file 11686_2025_1053_MOESM1_ESM.zip › Supplementary_Figure3_4_5_MicroscopyImages/Cyst-4.JPG]

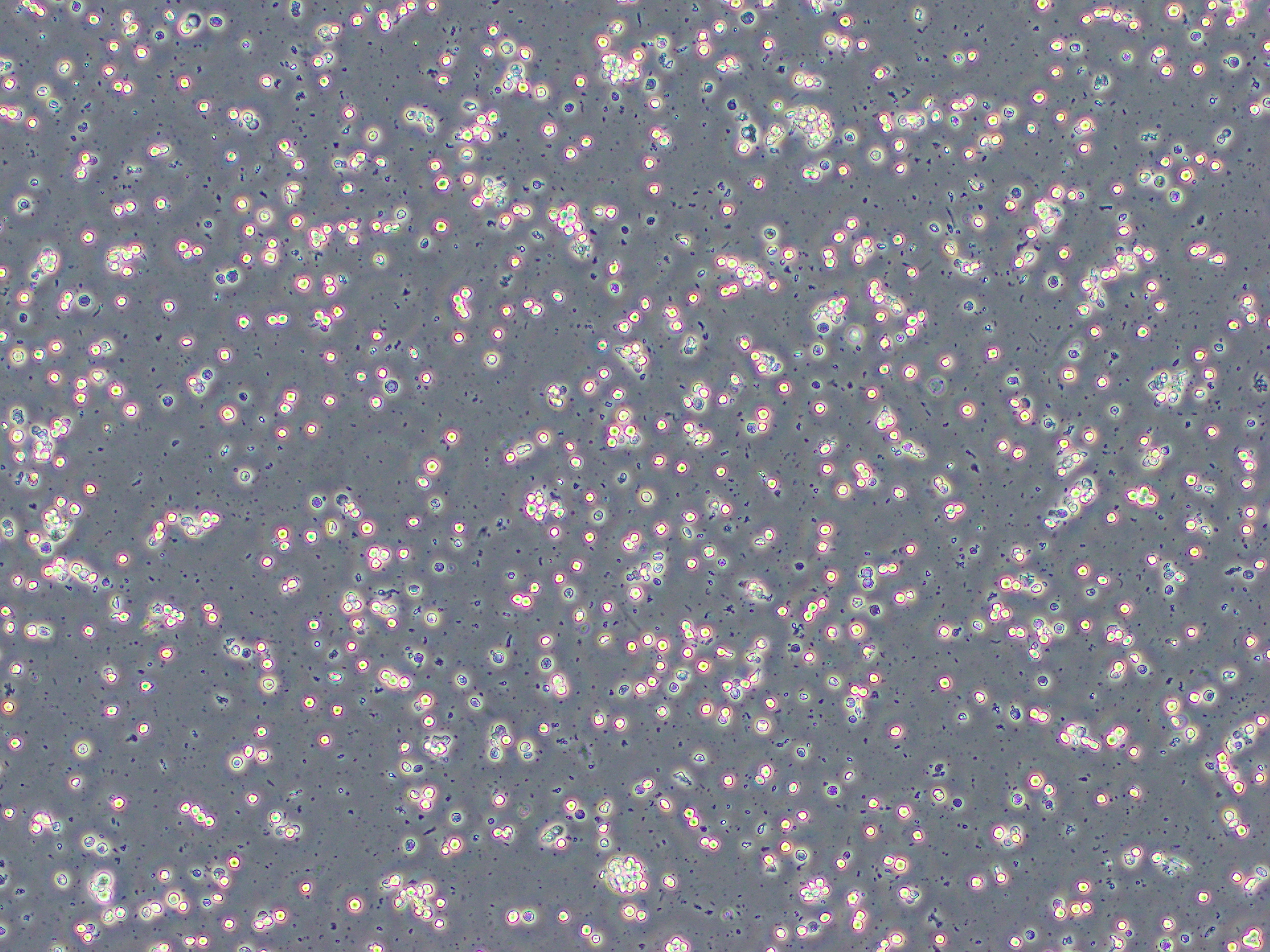

Supplement: Supplementary file 1 — Supplementary file1 (ZIP 208058 KB) [file 11686_2025_1053_MOESM1_ESM.zip › Supplementary_Figure3_4_5_MicroscopyImages/Cyst-40.JPG]

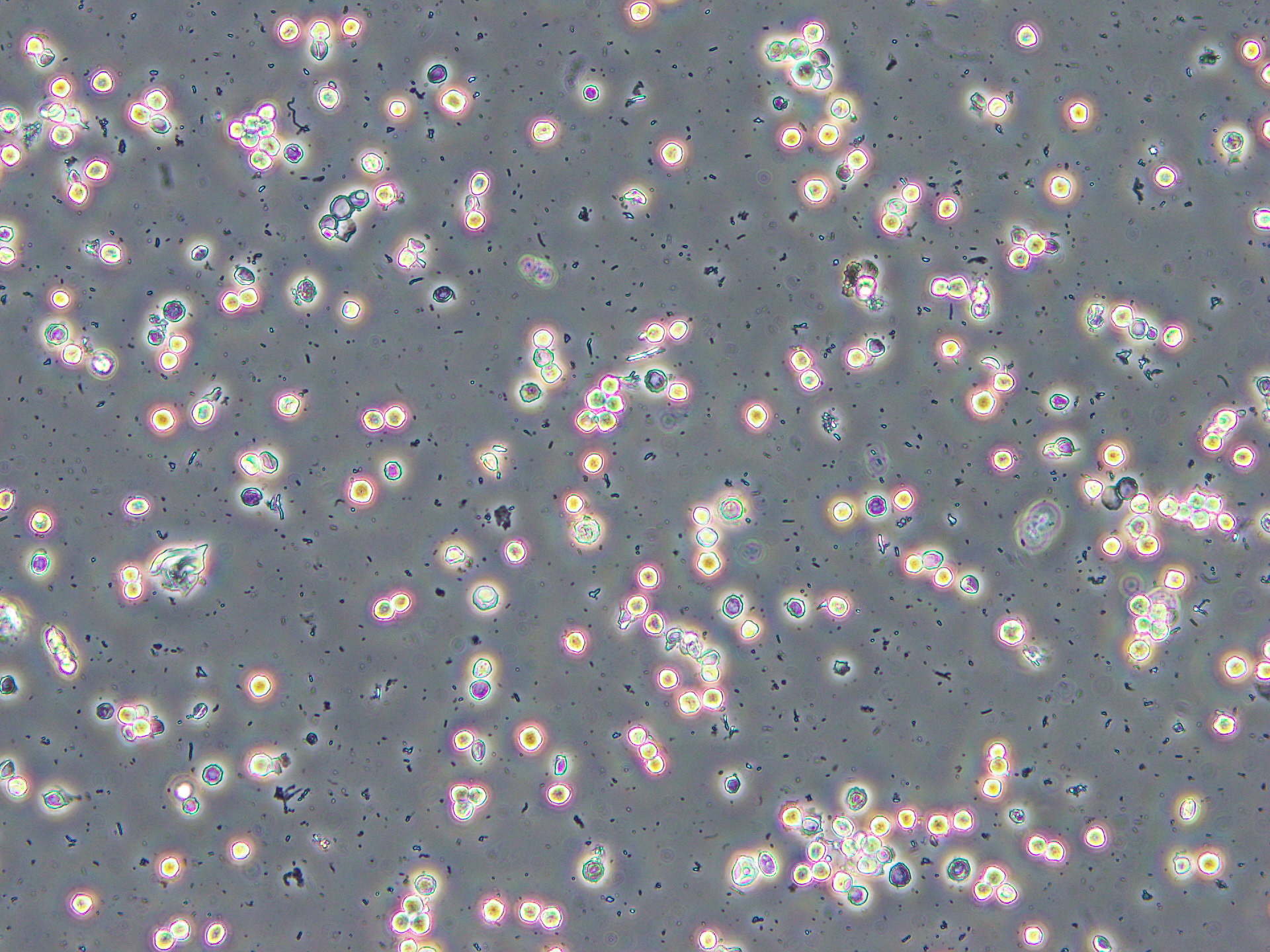

Supplement: Supplementary file 1 — Supplementary file1 (ZIP 208058 KB) [file 11686_2025_1053_MOESM1_ESM.zip › Supplementary_Figure3_4_5_MicroscopyImages/Cyst-41.JPG]

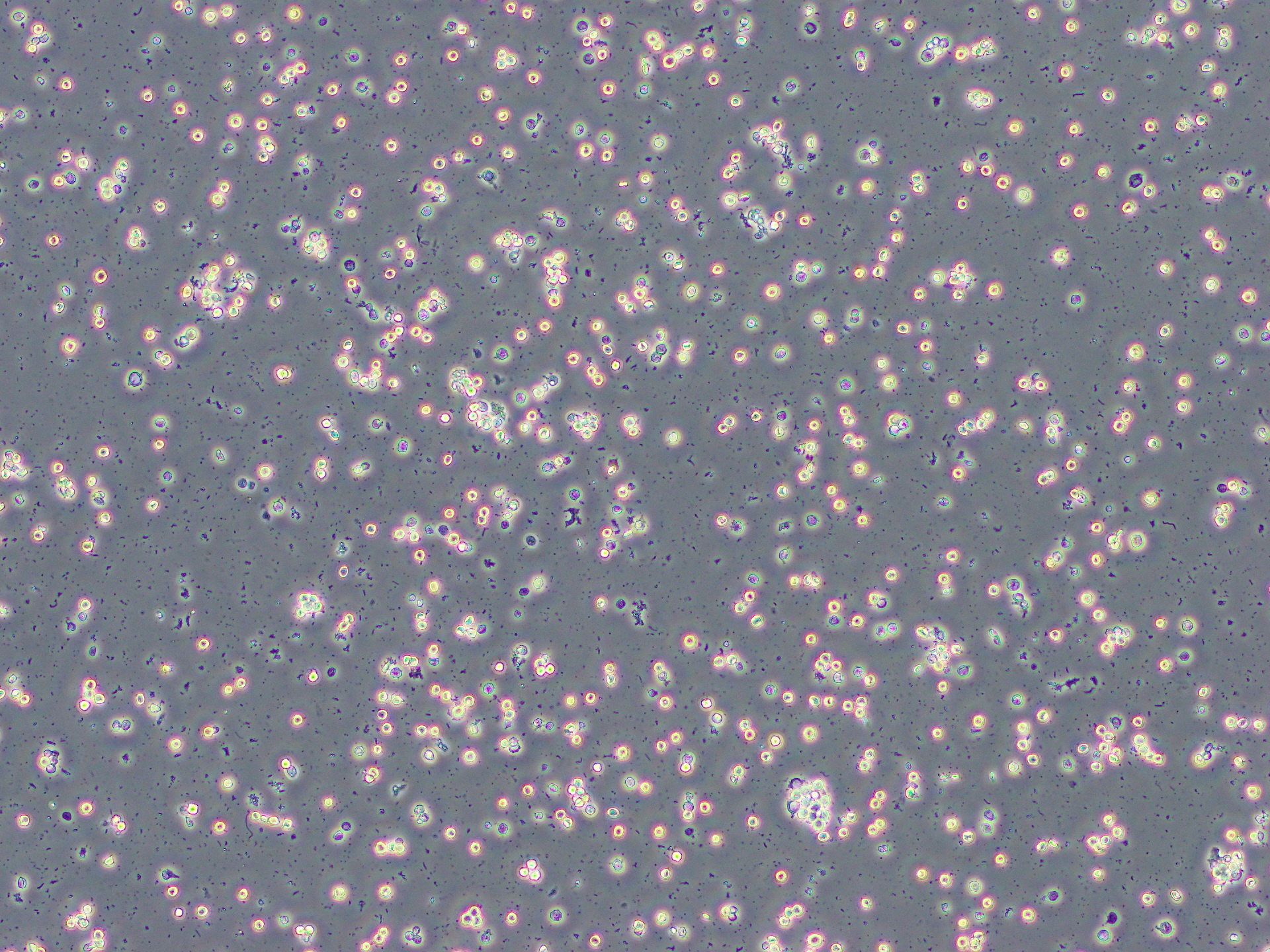

Supplement: Supplementary file 1 — Supplementary file1 (ZIP 208058 KB) [file 11686_2025_1053_MOESM1_ESM.zip › Supplementary_Figure3_4_5_MicroscopyImages/Cyst-42.JPG]

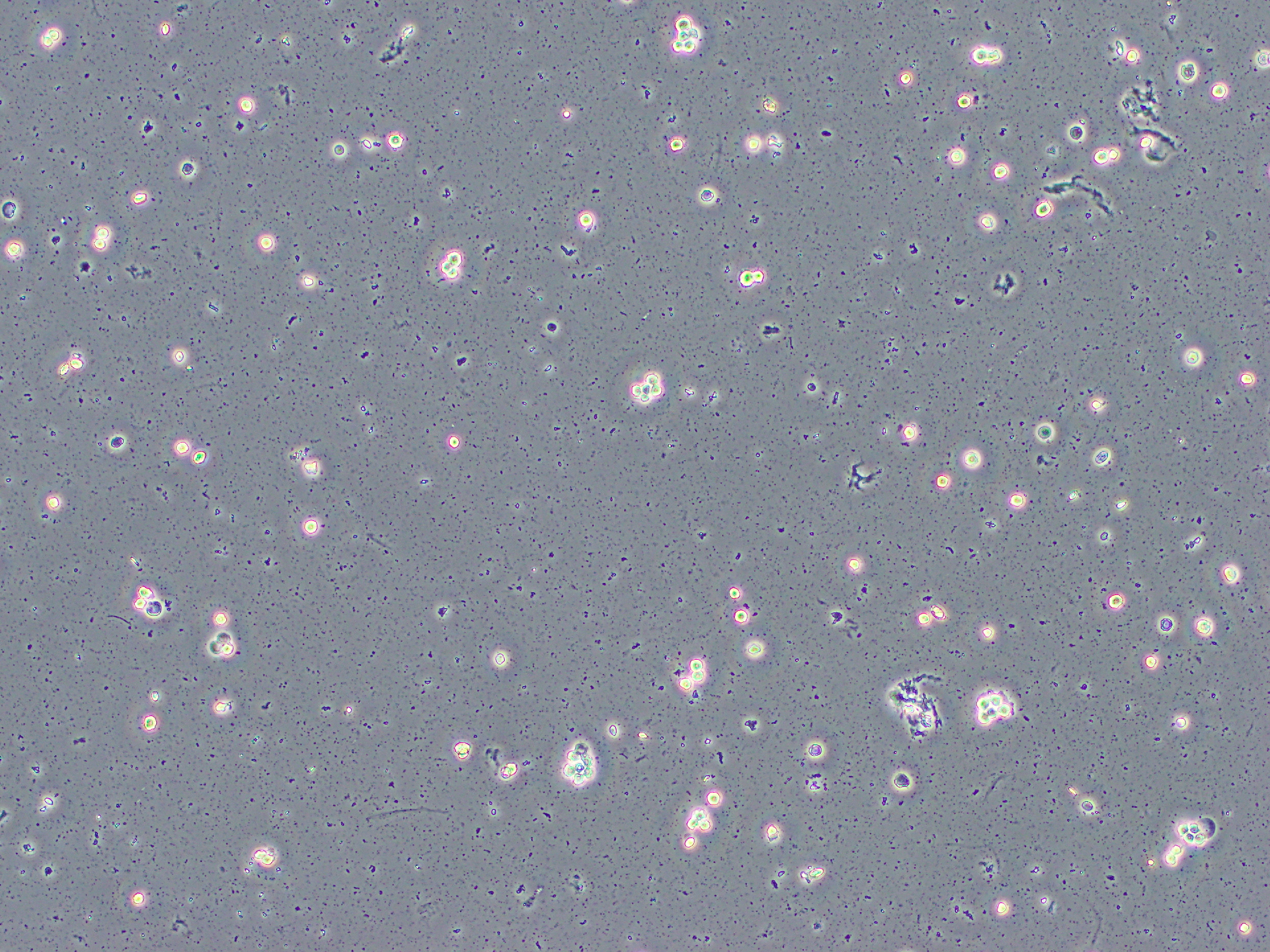

Supplement: Supplementary file 1 — Supplementary file1 (ZIP 208058 KB) [file 11686_2025_1053_MOESM1_ESM.zip › Supplementary_Figure3_4_5_MicroscopyImages/Cyst-5.JPG]

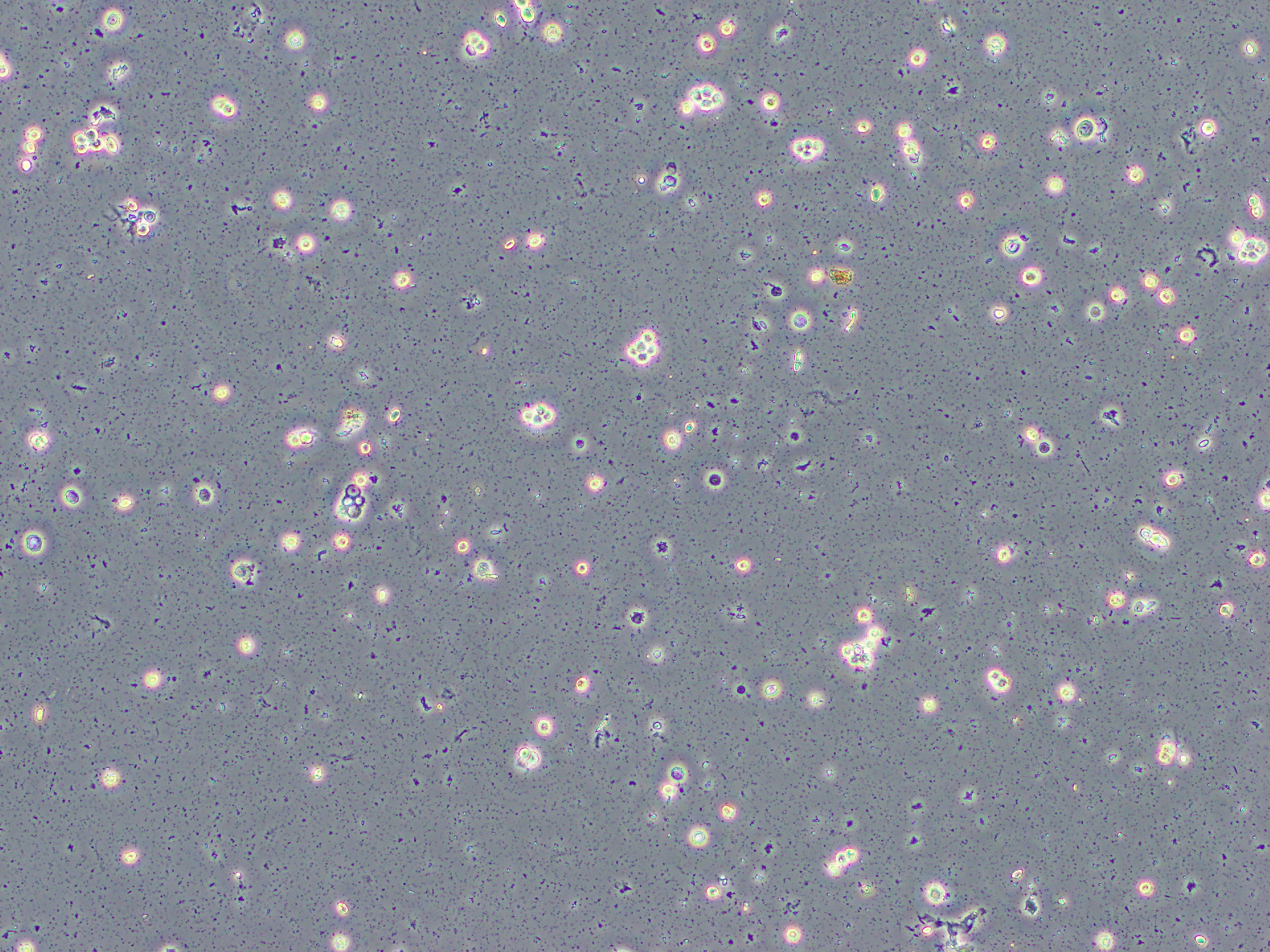

Supplement: Supplementary file 1 — Supplementary file1 (ZIP 208058 KB) [file 11686_2025_1053_MOESM1_ESM.zip › Supplementary_Figure3_4_5_MicroscopyImages/Cyst-6.JPG]

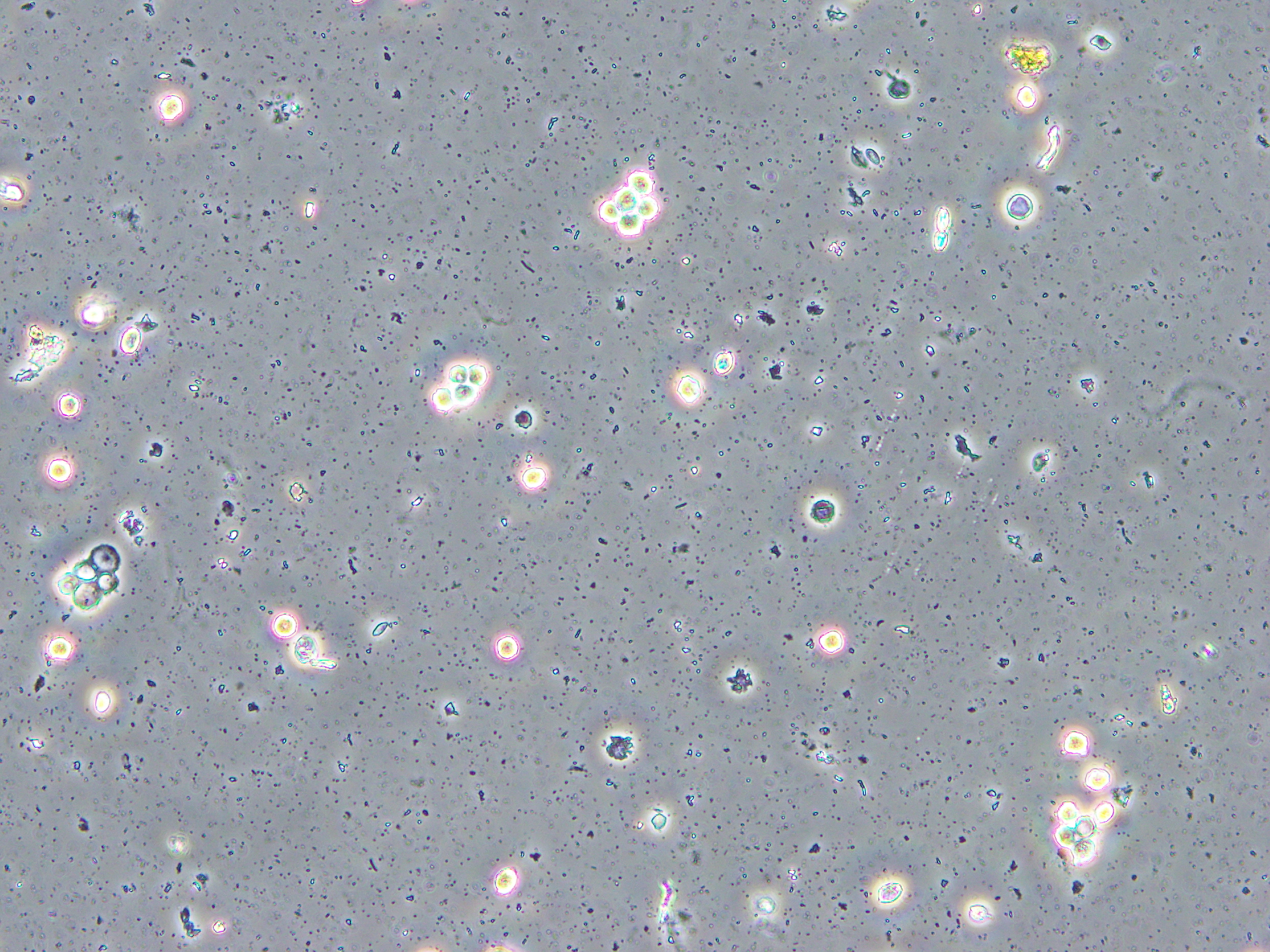

Supplement: Supplementary file 1 — Supplementary file1 (ZIP 208058 KB) [file 11686_2025_1053_MOESM1_ESM.zip › Supplementary_Figure3_4_5_MicroscopyImages/Cyst-7.JPG]

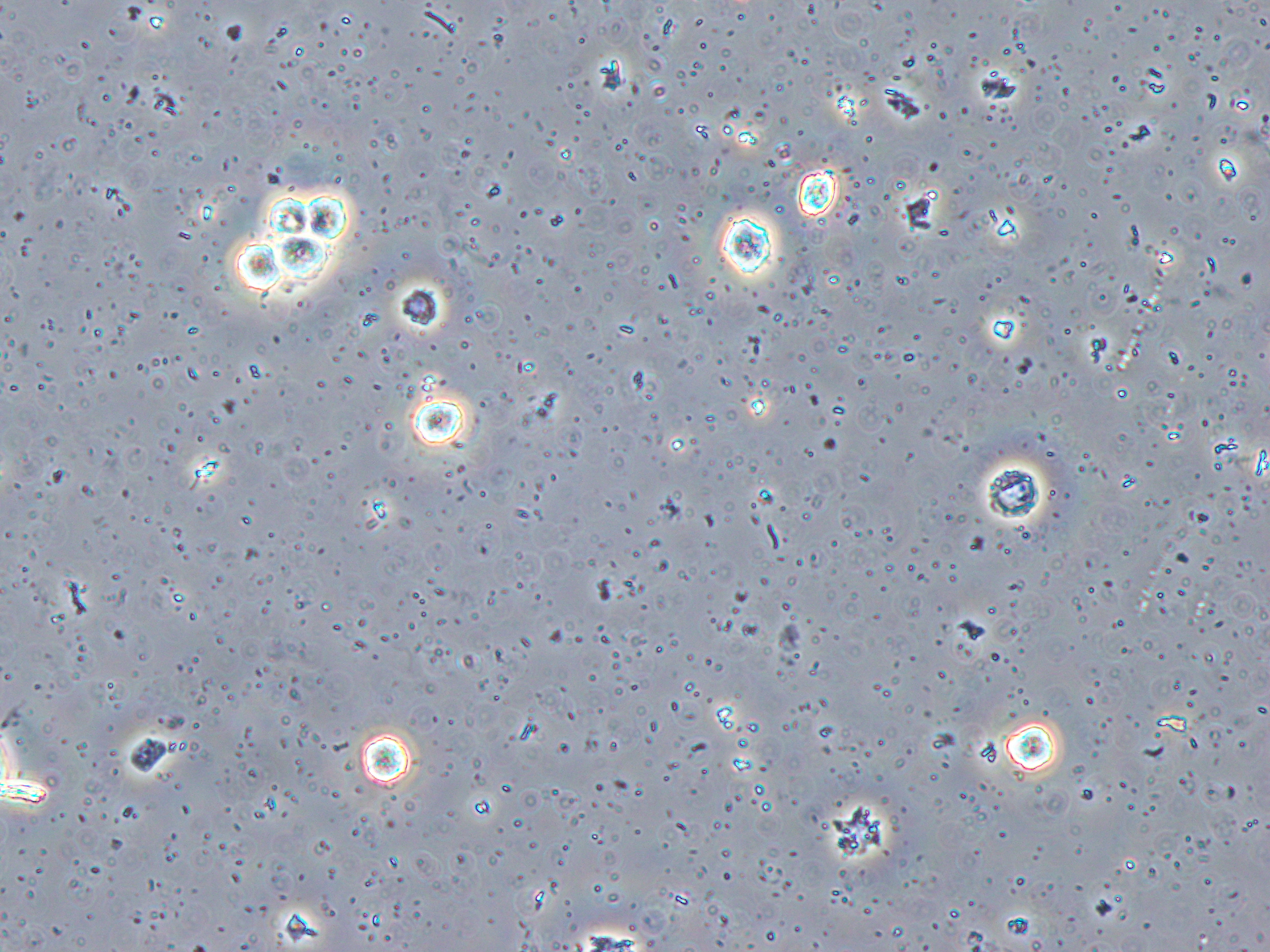

Supplement: Supplementary file 1 — Supplementary file1 (ZIP 208058 KB) [file 11686_2025_1053_MOESM1_ESM.zip › Supplementary_Figure3_4_5_MicroscopyImages/Cyst-8.JPG]

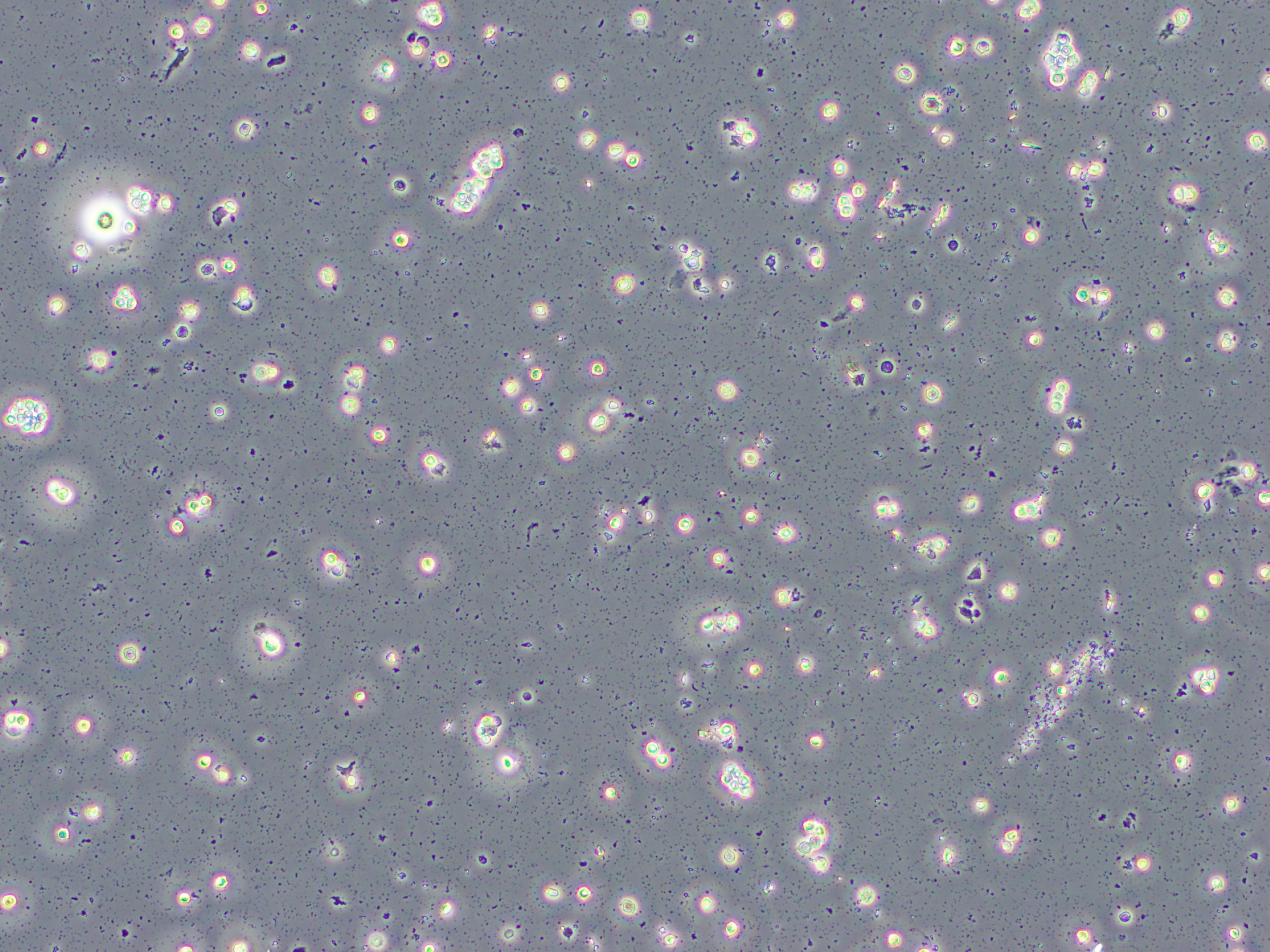

Supplement: Supplementary file 1 — Supplementary file1 (ZIP 208058 KB) [file 11686_2025_1053_MOESM1_ESM.zip › Supplementary_Figure3_4_5_MicroscopyImages/Cyst-9.JPG]

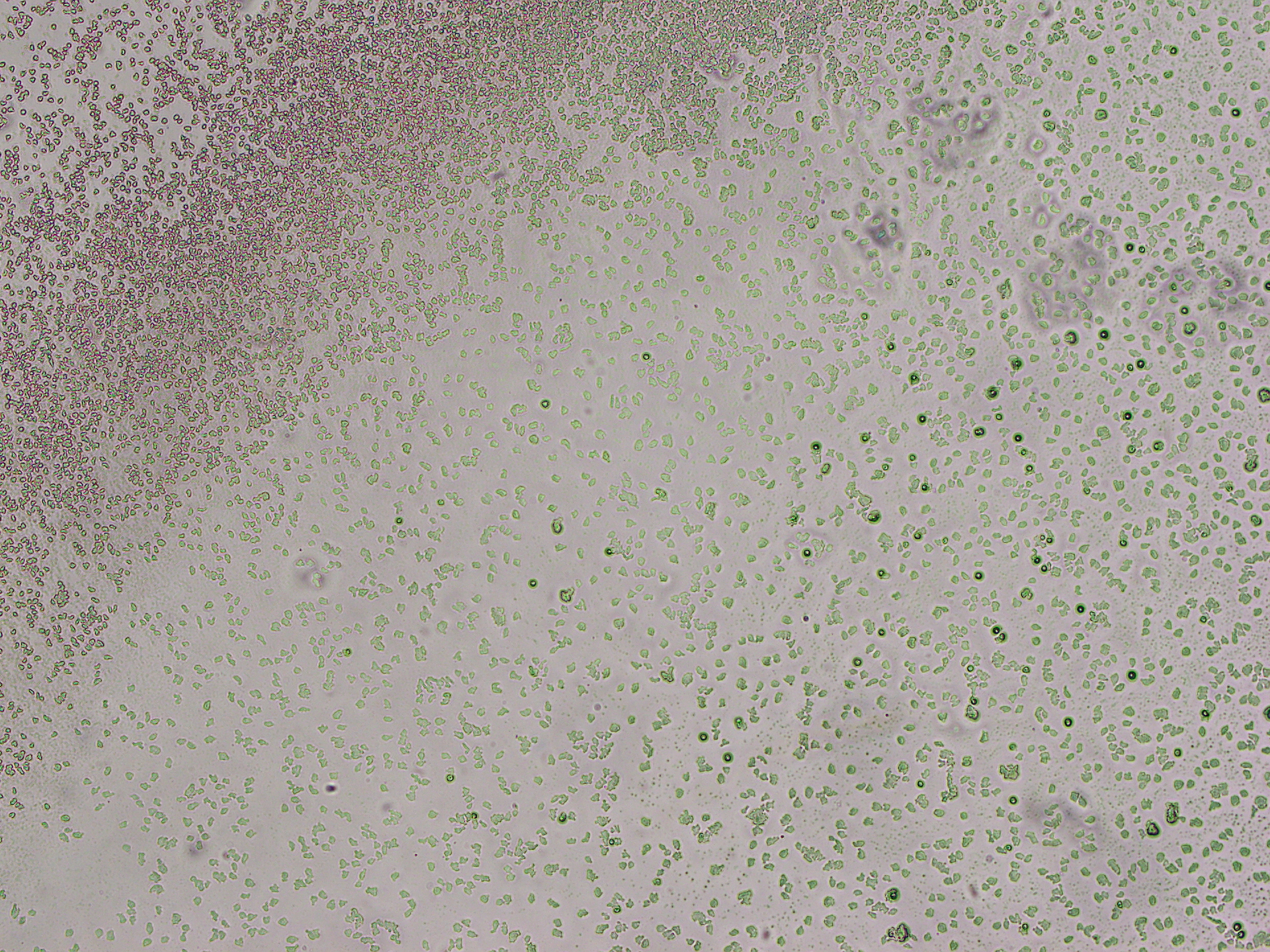

Supplement: Supplementary file 1 — Supplementary file1 (ZIP 208058 KB) [file 11686_2025_1053_MOESM1_ESM.zip › Supplementary_Figure3_4_5_MicroscopyImages/Trophozoite-1.JPG]

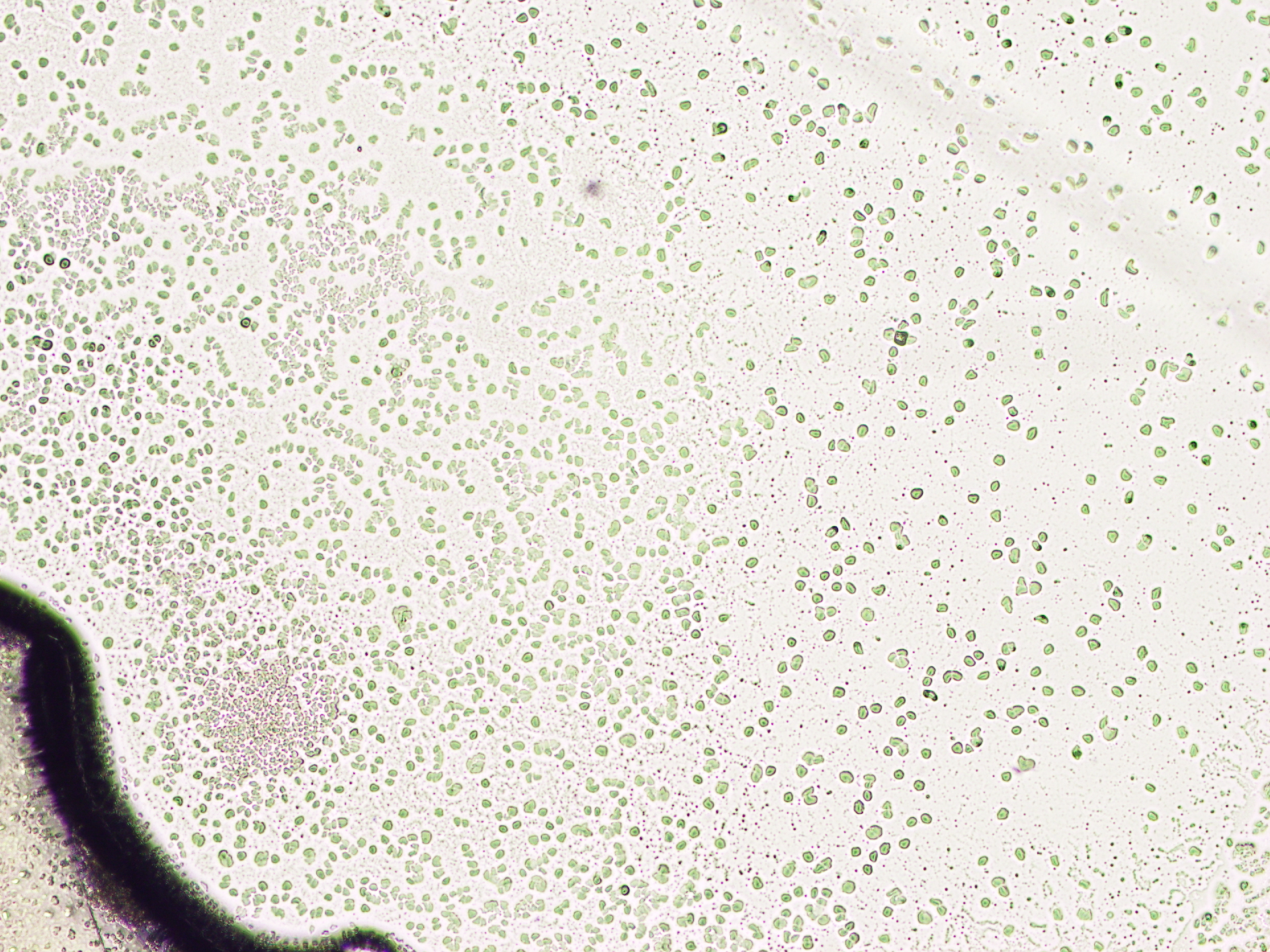

Supplement: Supplementary file 1 — Supplementary file1 (ZIP 208058 KB) [file 11686_2025_1053_MOESM1_ESM.zip › Supplementary_Figure3_4_5_MicroscopyImages/Trophozoite-10.JPG]

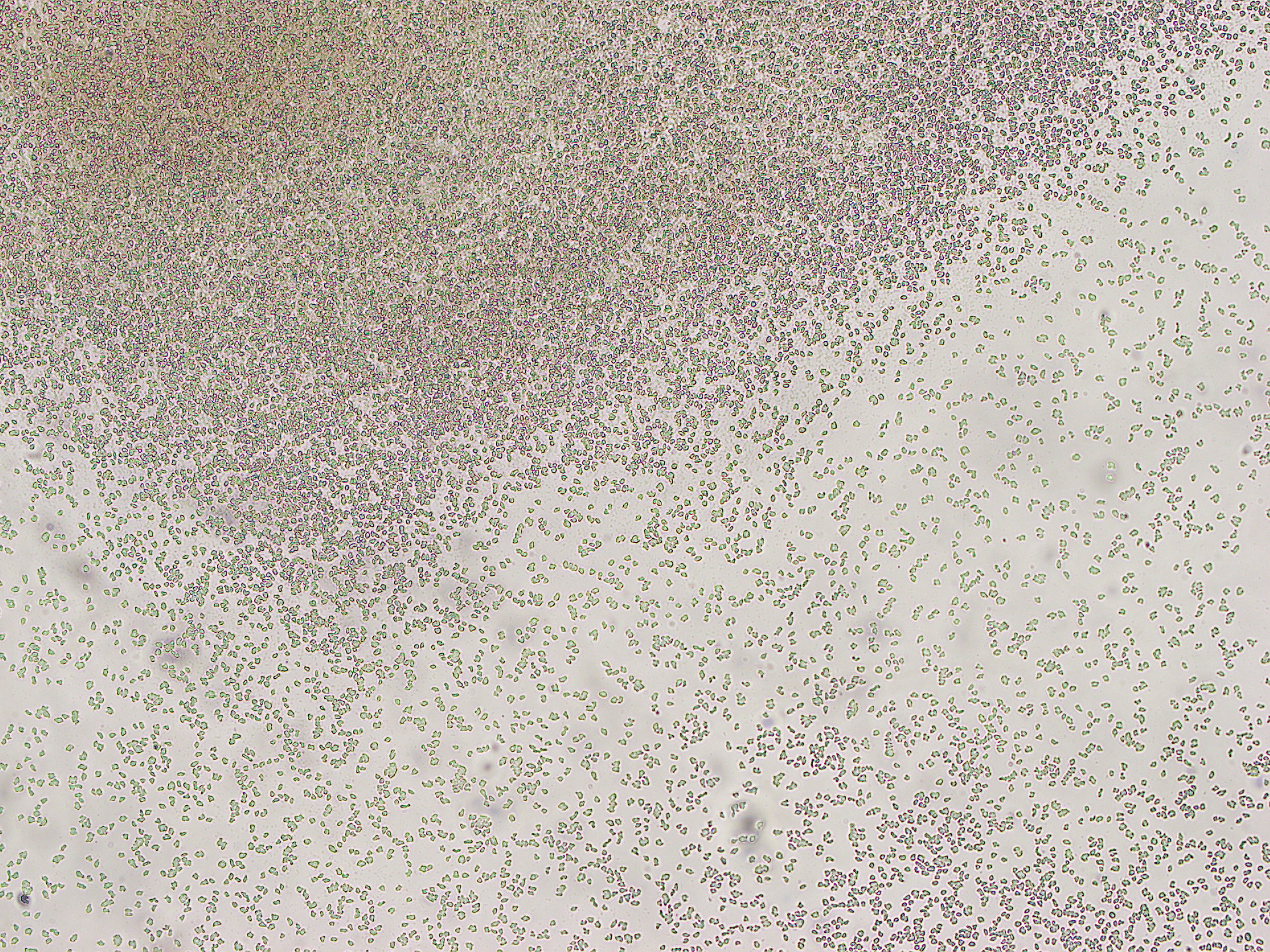

Supplement: Supplementary file 1 — Supplementary file1 (ZIP 208058 KB) [file 11686_2025_1053_MOESM1_ESM.zip › Supplementary_Figure3_4_5_MicroscopyImages/Trophozoite-11.JPG]

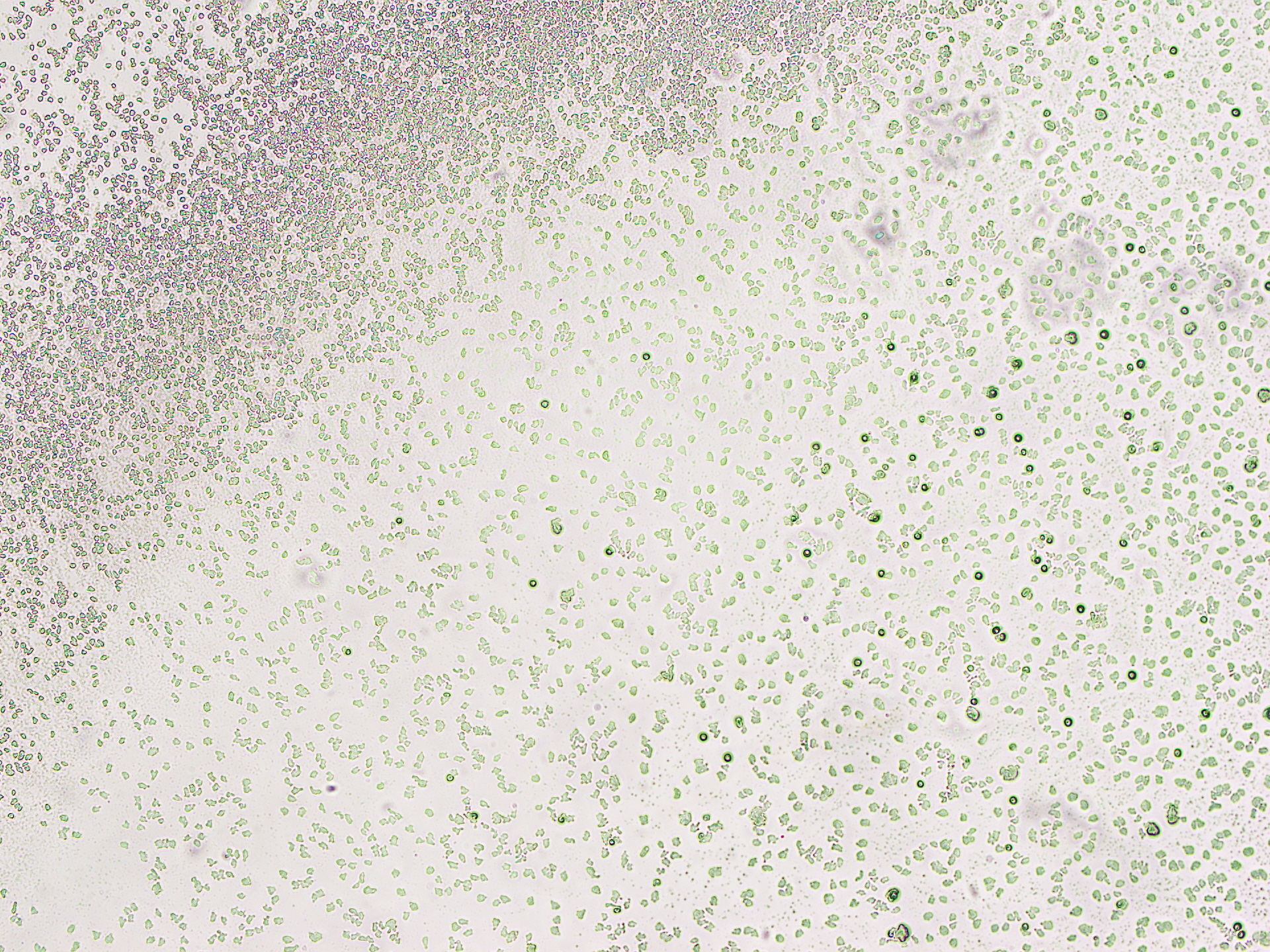

Supplement: Supplementary file 1 — Supplementary file1 (ZIP 208058 KB) [file 11686_2025_1053_MOESM1_ESM.zip › Supplementary_Figure3_4_5_MicroscopyImages/Trophozoite-12.JPG]

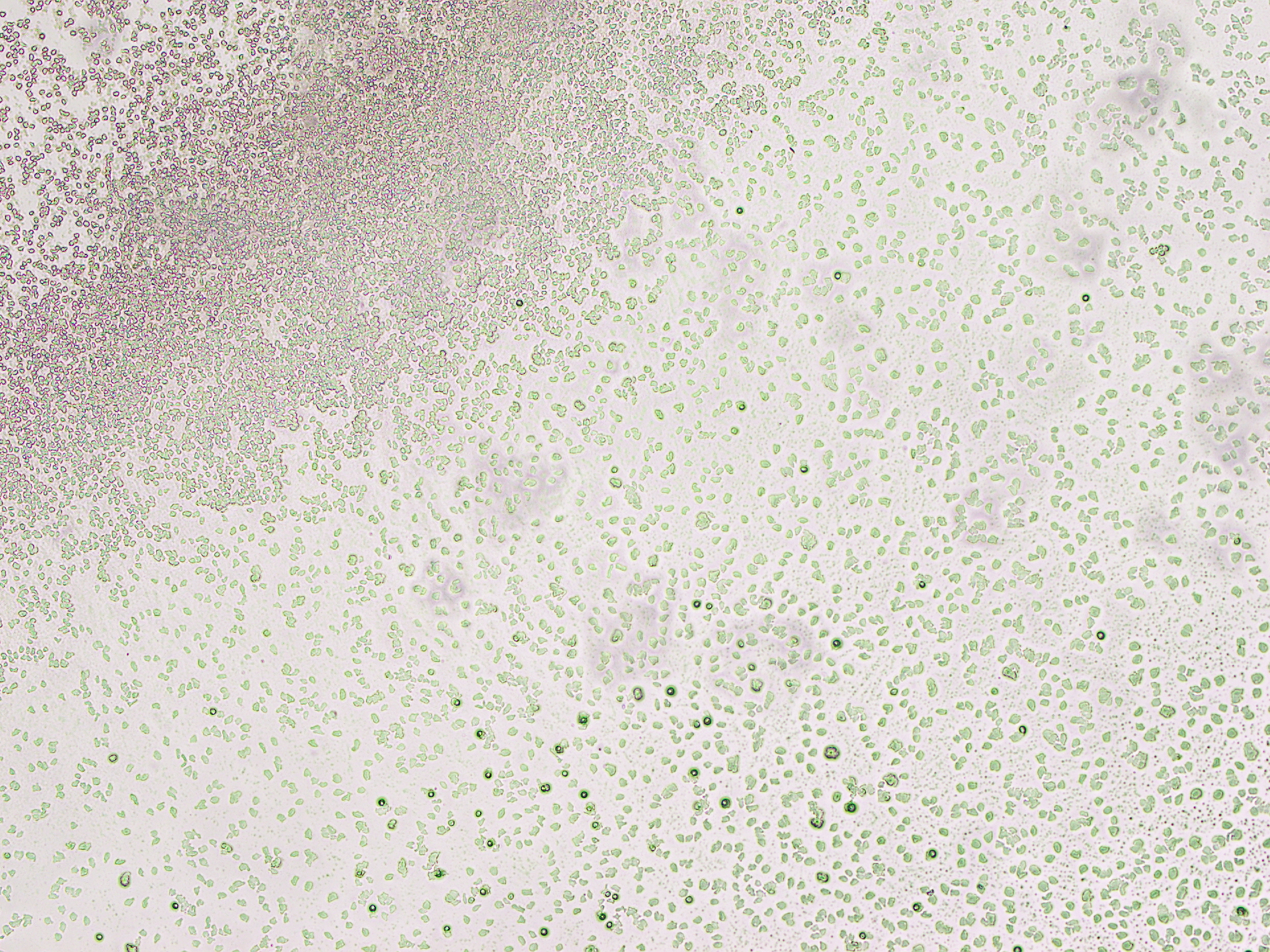

Supplement: Supplementary file 1 — Supplementary file1 (ZIP 208058 KB) [file 11686_2025_1053_MOESM1_ESM.zip › Supplementary_Figure3_4_5_MicroscopyImages/Trophozoite-13.JPG]

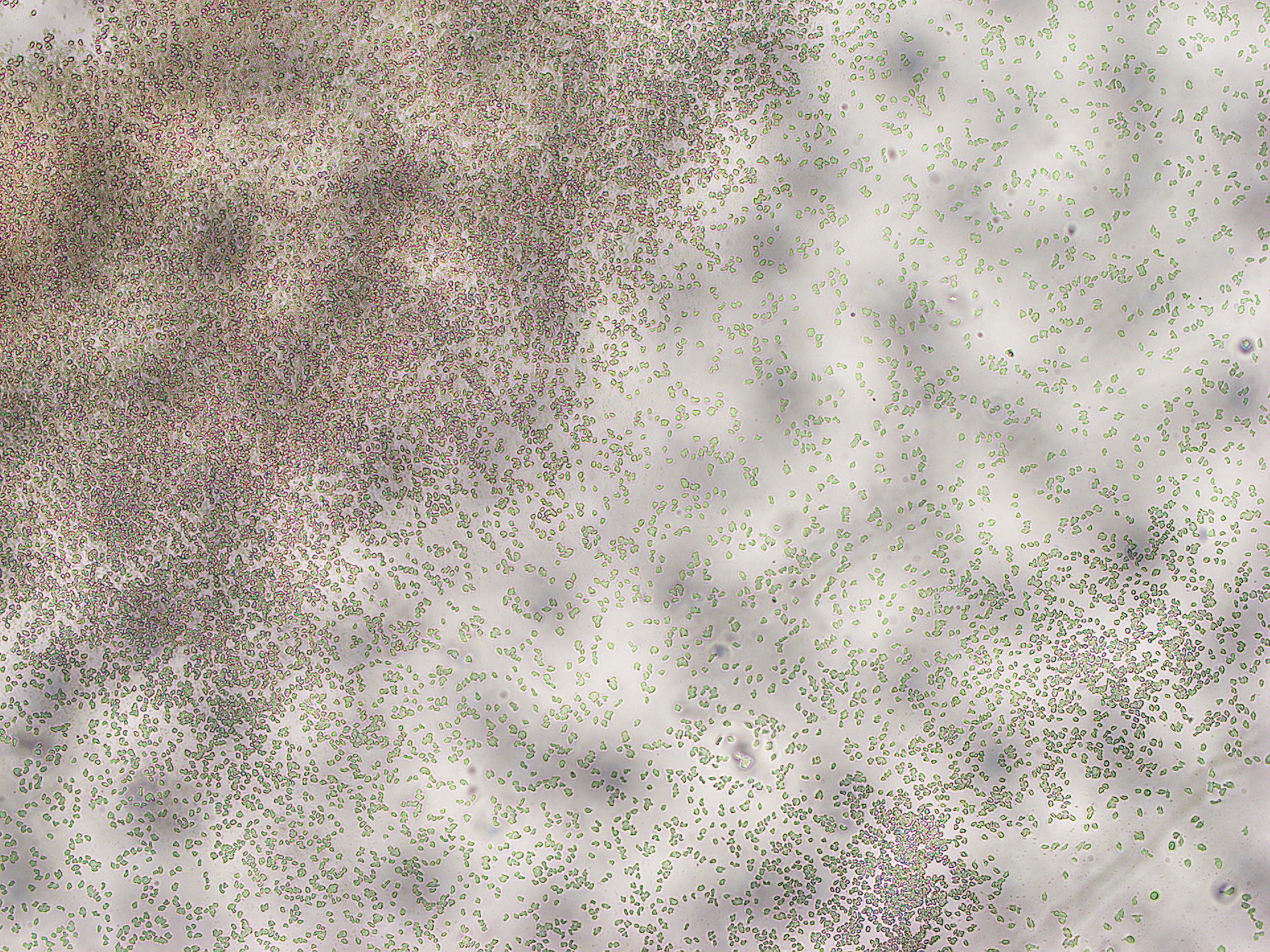

Supplement: Supplementary file 1 — Supplementary file1 (ZIP 208058 KB) [file 11686_2025_1053_MOESM1_ESM.zip › Supplementary_Figure3_4_5_MicroscopyImages/Trophozoite-14.JPG]

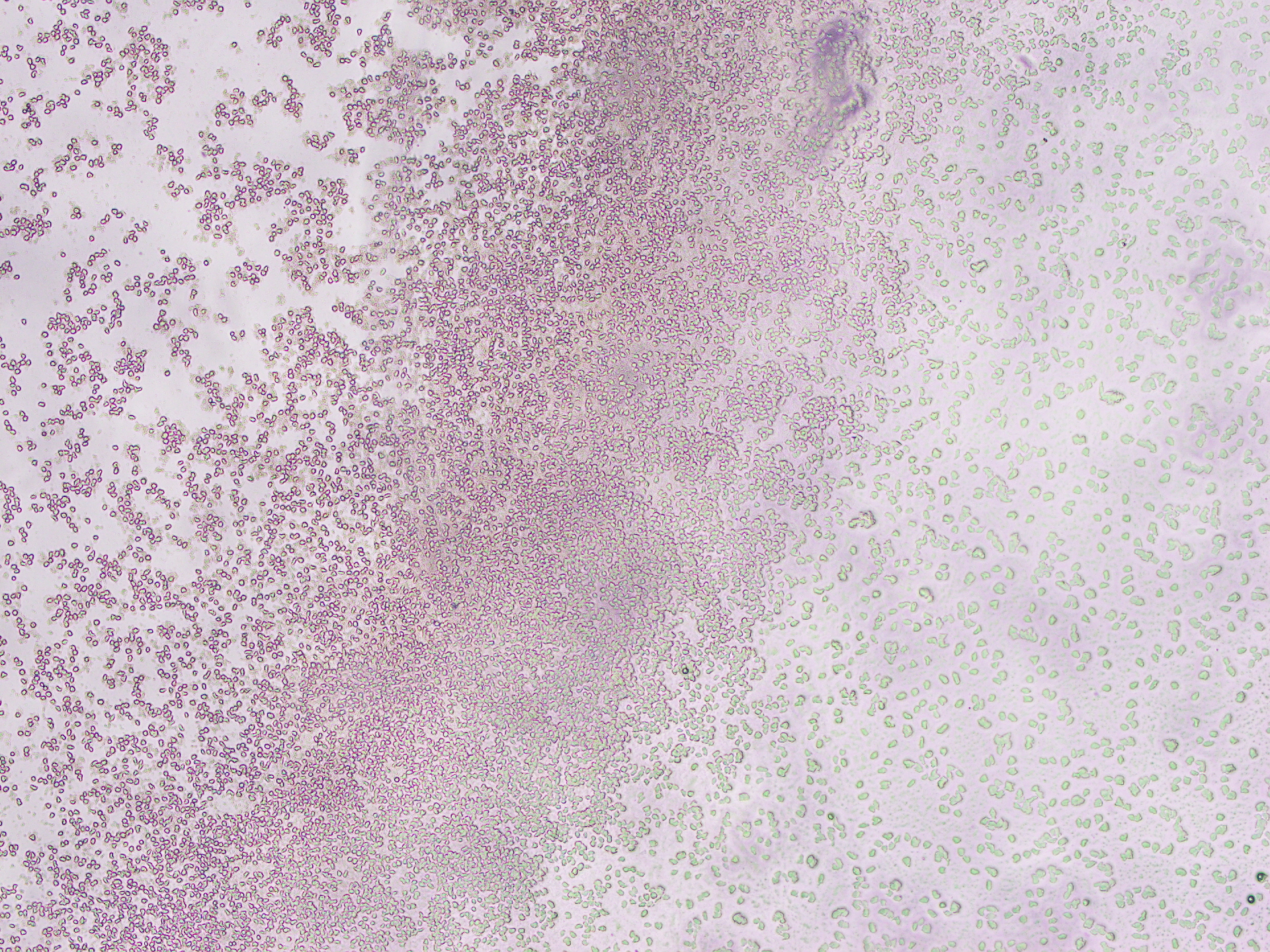

Supplement: Supplementary file 1 — Supplementary file1 (ZIP 208058 KB) [file 11686_2025_1053_MOESM1_ESM.zip › Supplementary_Figure3_4_5_MicroscopyImages/Trophozoite-15.JPG]

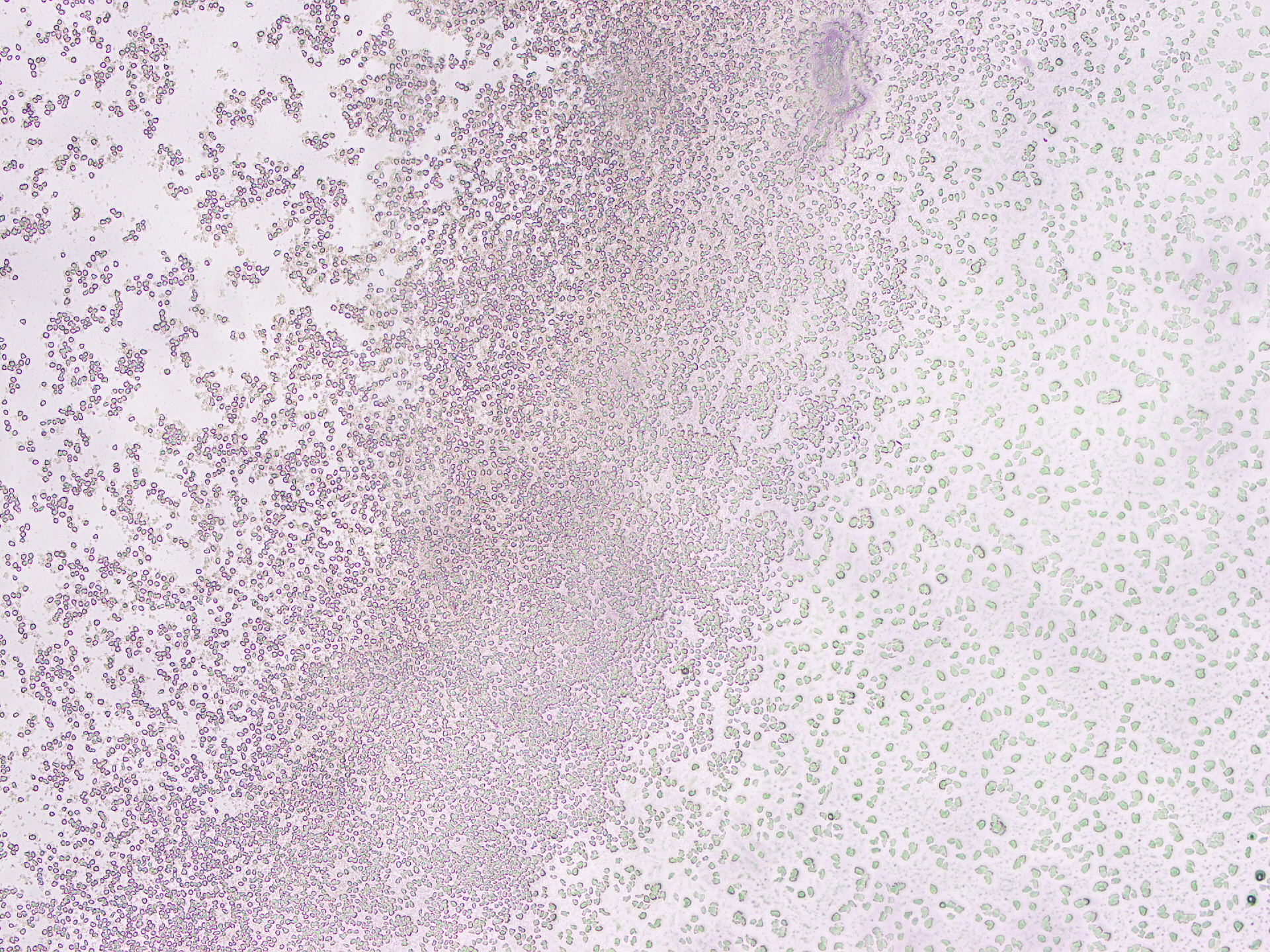

Supplement: Supplementary file 1 — Supplementary file1 (ZIP 208058 KB) [file 11686_2025_1053_MOESM1_ESM.zip › Supplementary_Figure3_4_5_MicroscopyImages/Trophozoite-16.JPG]

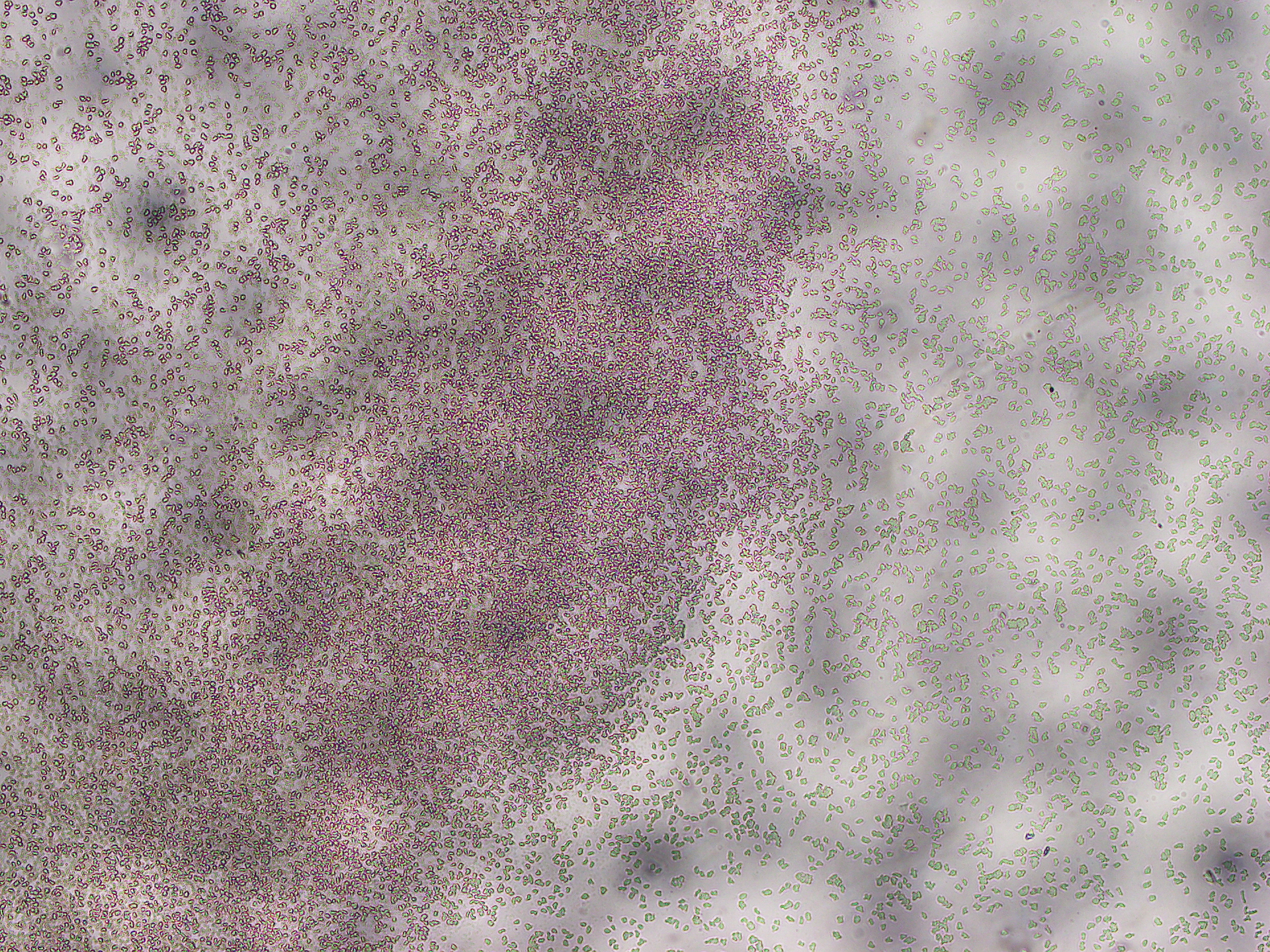

Supplement: Supplementary file 1 — Supplementary file1 (ZIP 208058 KB) [file 11686_2025_1053_MOESM1_ESM.zip › Supplementary_Figure3_4_5_MicroscopyImages/Trophozoite-17.JPG]

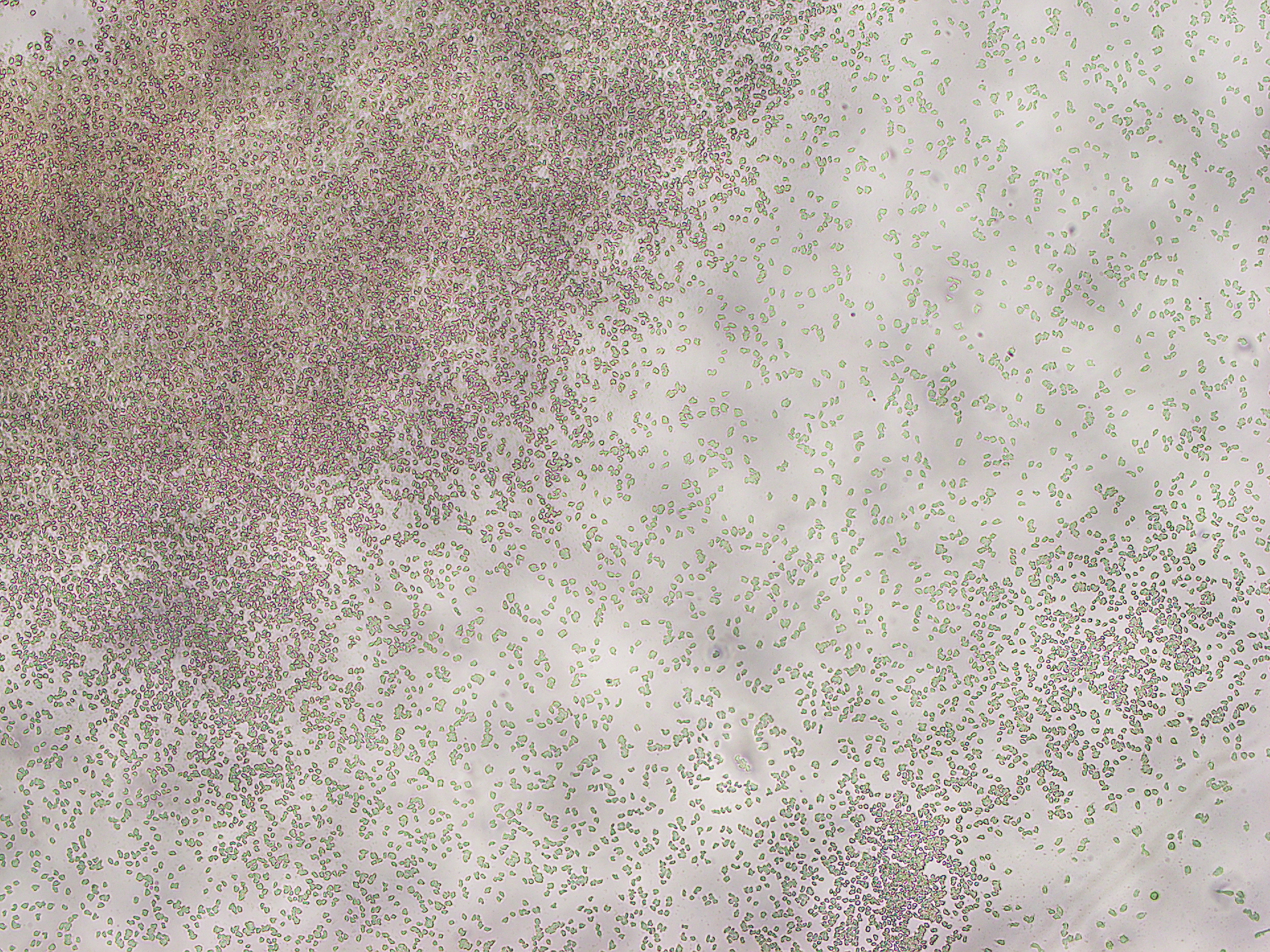

Supplement: Supplementary file 1 — Supplementary file1 (ZIP 208058 KB) [file 11686_2025_1053_MOESM1_ESM.zip › Supplementary_Figure3_4_5_MicroscopyImages/Trophozoite-18.JPG]

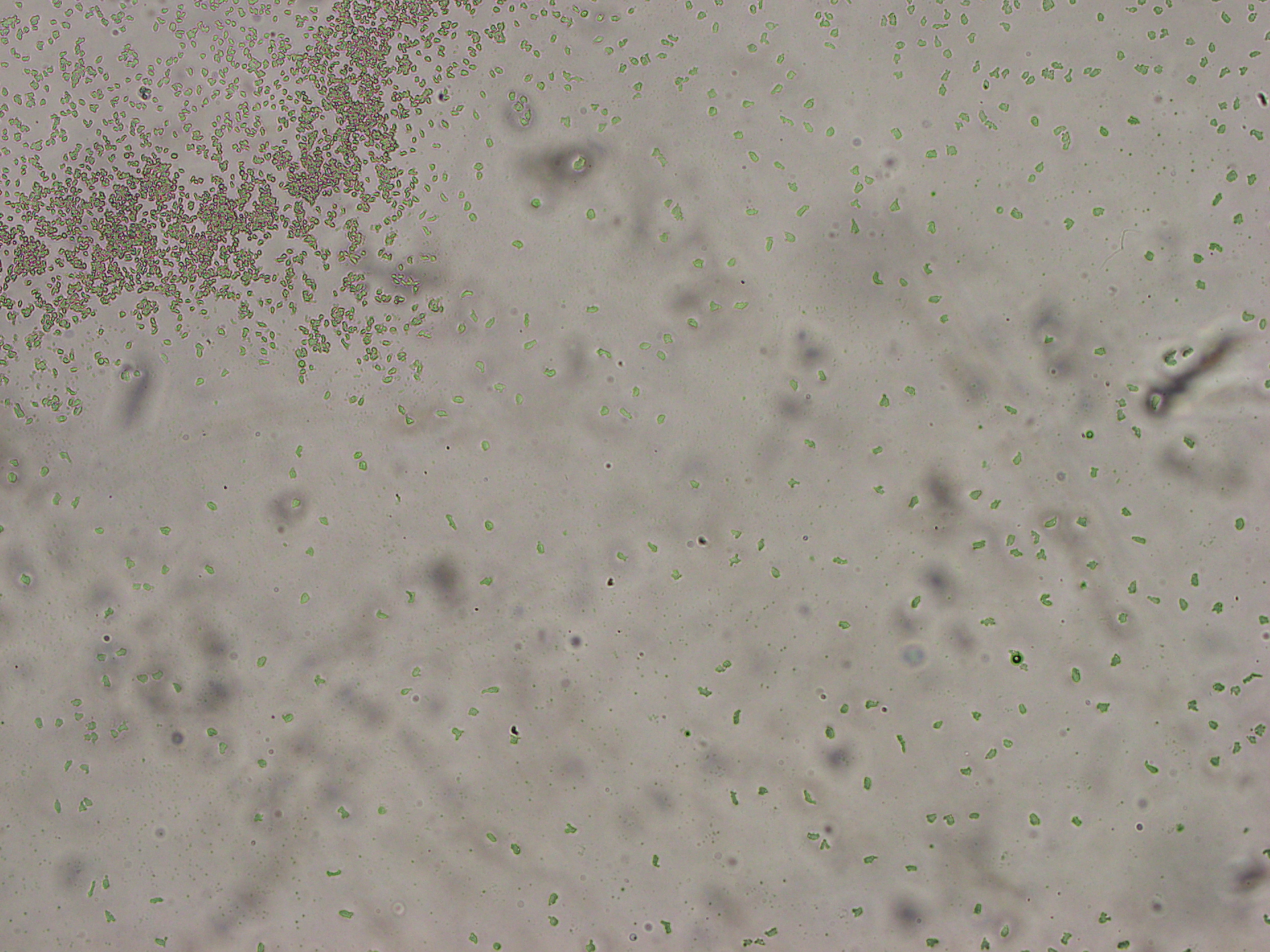

Supplement: Supplementary file 1 — Supplementary file1 (ZIP 208058 KB) [file 11686_2025_1053_MOESM1_ESM.zip › Supplementary_Figure3_4_5_MicroscopyImages/Trophozoite-19.JPG]

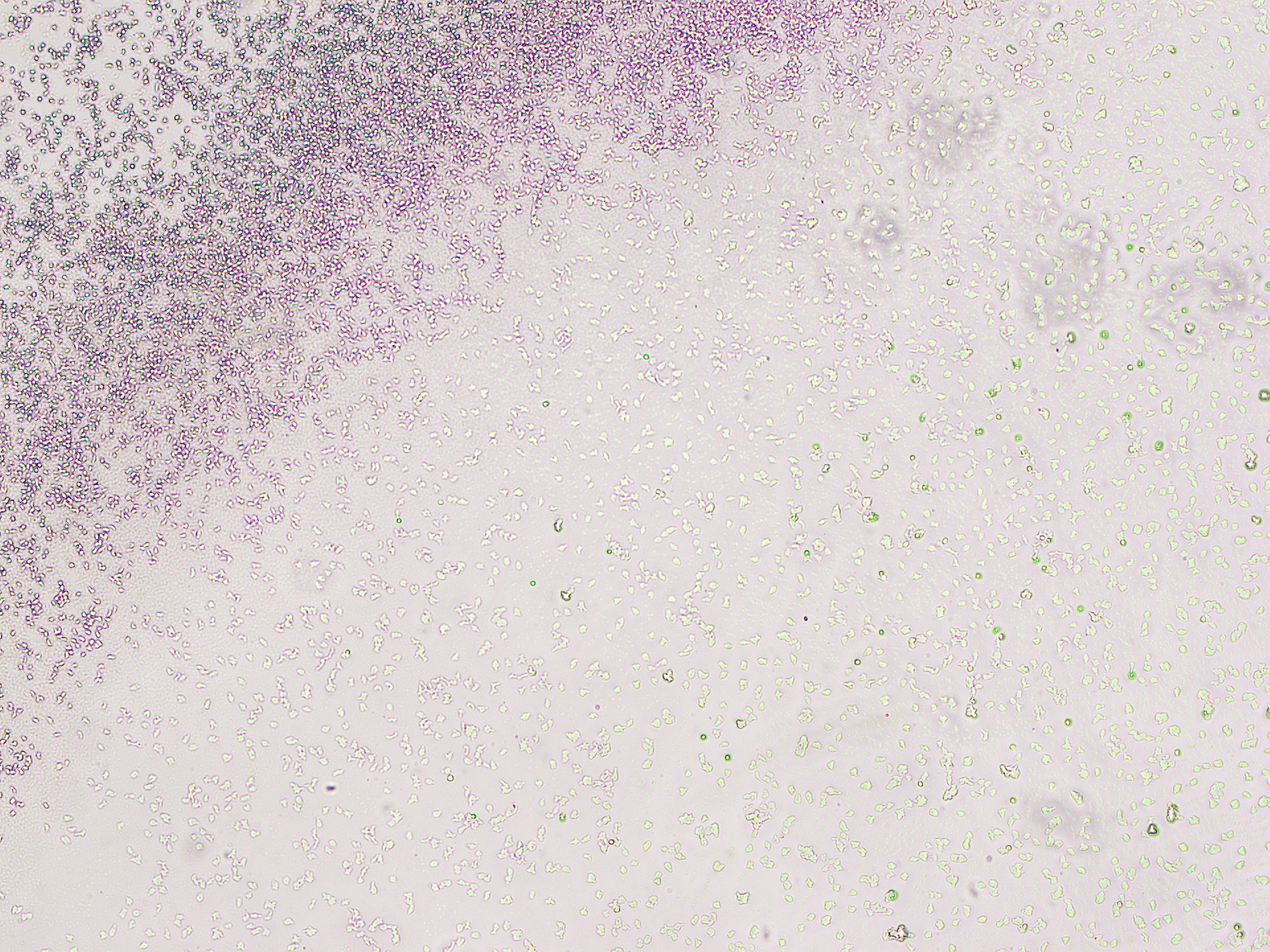

Supplement: Supplementary file 1 — Supplementary file1 (ZIP 208058 KB) [file 11686_2025_1053_MOESM1_ESM.zip › Supplementary_Figure3_4_5_MicroscopyImages/Trophozoite-2.JPG]

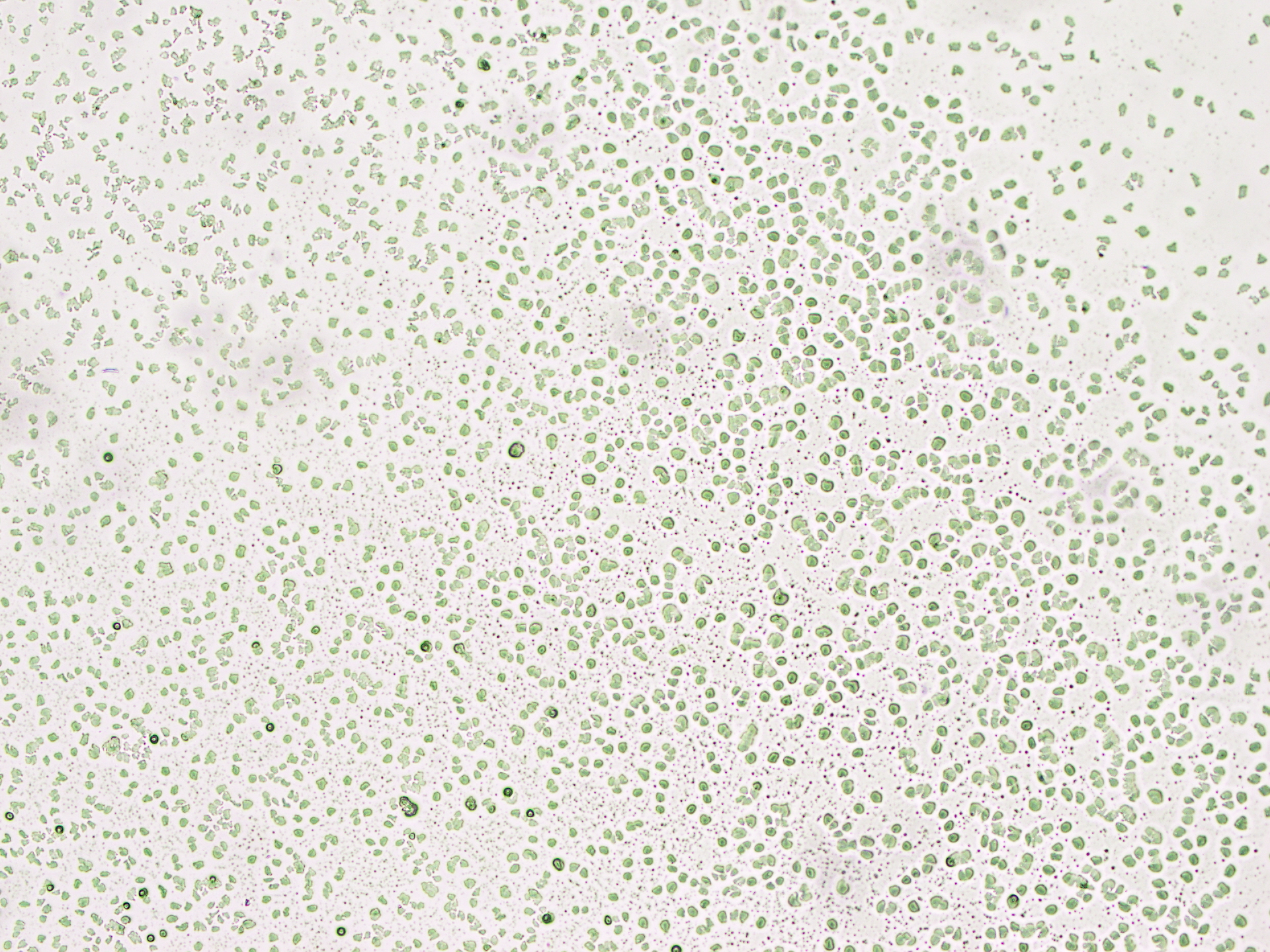

Supplement: Supplementary file 1 — Supplementary file1 (ZIP 208058 KB) [file 11686_2025_1053_MOESM1_ESM.zip › Supplementary_Figure3_4_5_MicroscopyImages/Trophozoite-20.JPG]

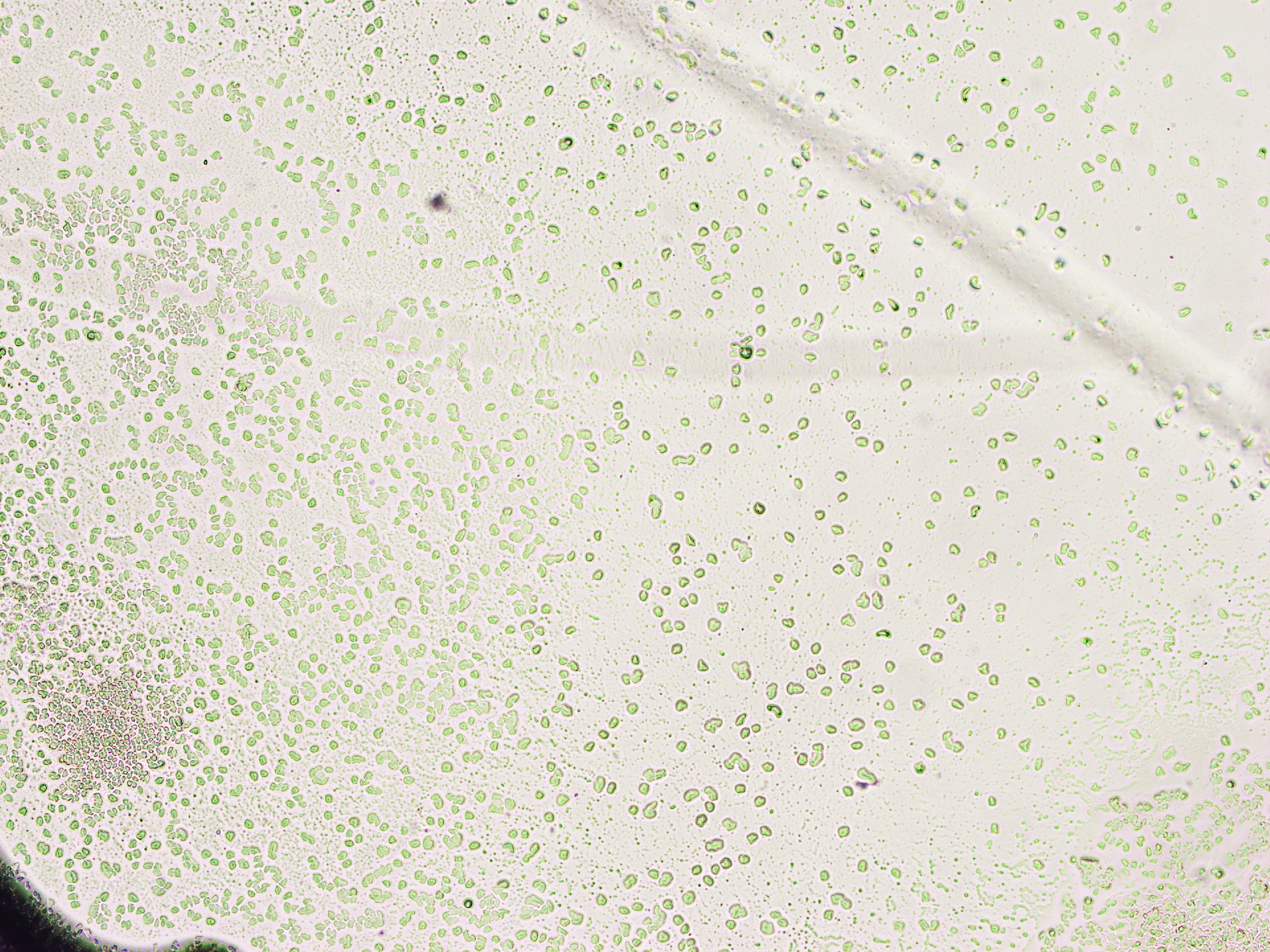

Supplement: Supplementary file 1 — Supplementary file1 (ZIP 208058 KB) [file 11686_2025_1053_MOESM1_ESM.zip › Supplementary_Figure3_4_5_MicroscopyImages/Trophozoite-21.JPG]

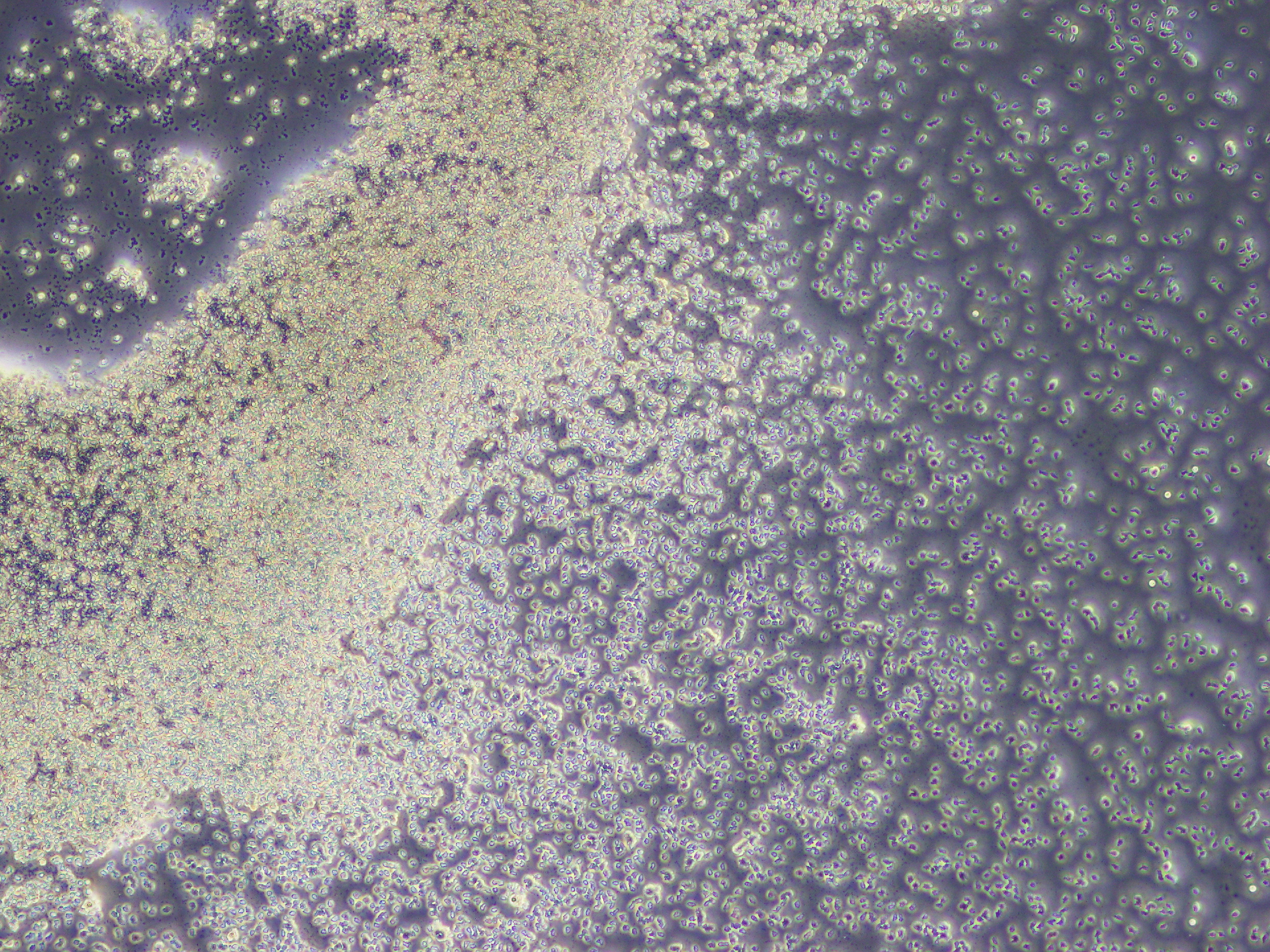

Supplement: Supplementary file 1 — Supplementary file1 (ZIP 208058 KB) [file 11686_2025_1053_MOESM1_ESM.zip › Supplementary_Figure3_4_5_MicroscopyImages/Trophozoite-22.JPG]

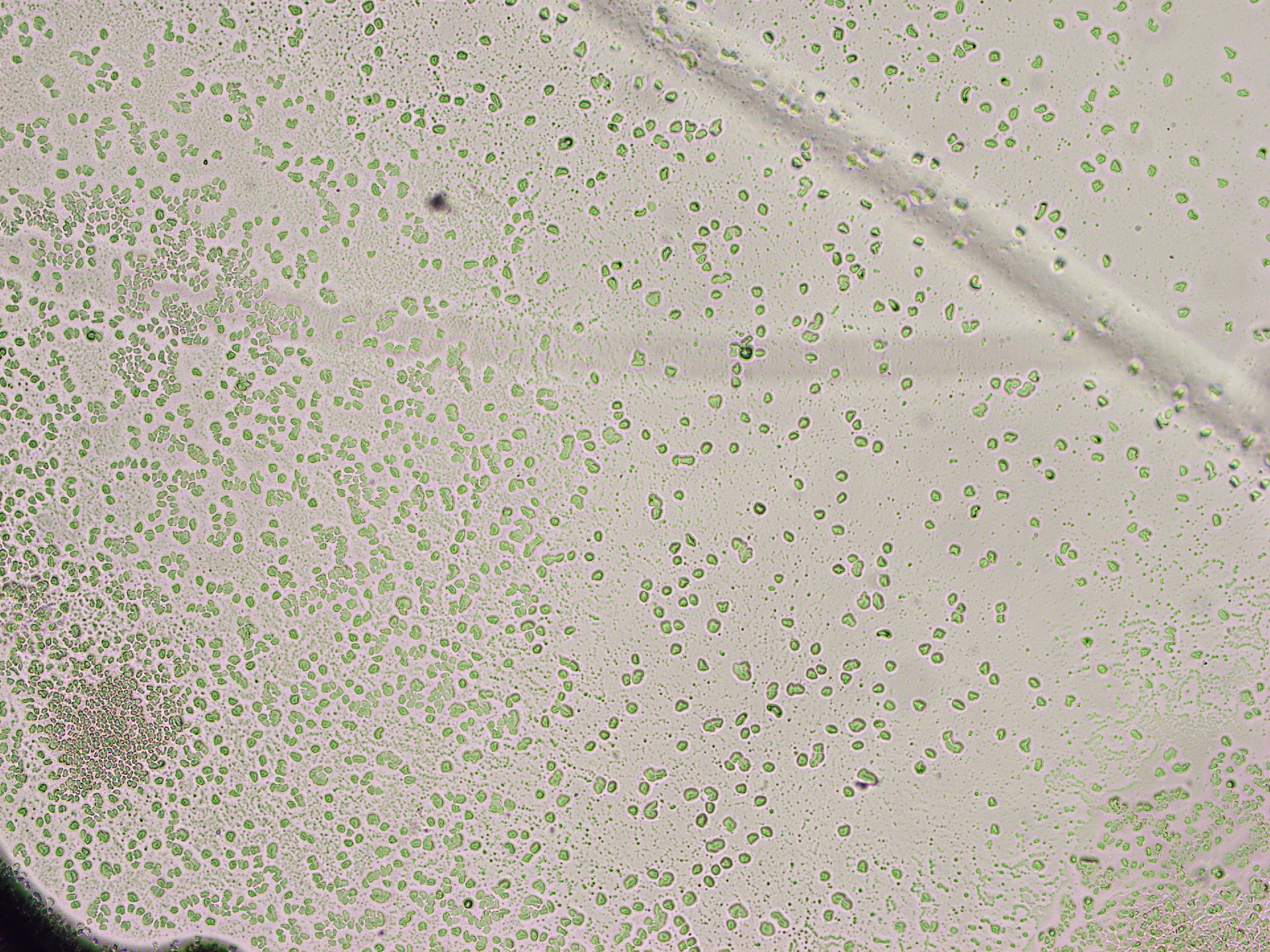

Supplement: Supplementary file 1 — Supplementary file1 (ZIP 208058 KB) [file 11686_2025_1053_MOESM1_ESM.zip › Supplementary_Figure3_4_5_MicroscopyImages/Trophozoite-23.JPG]

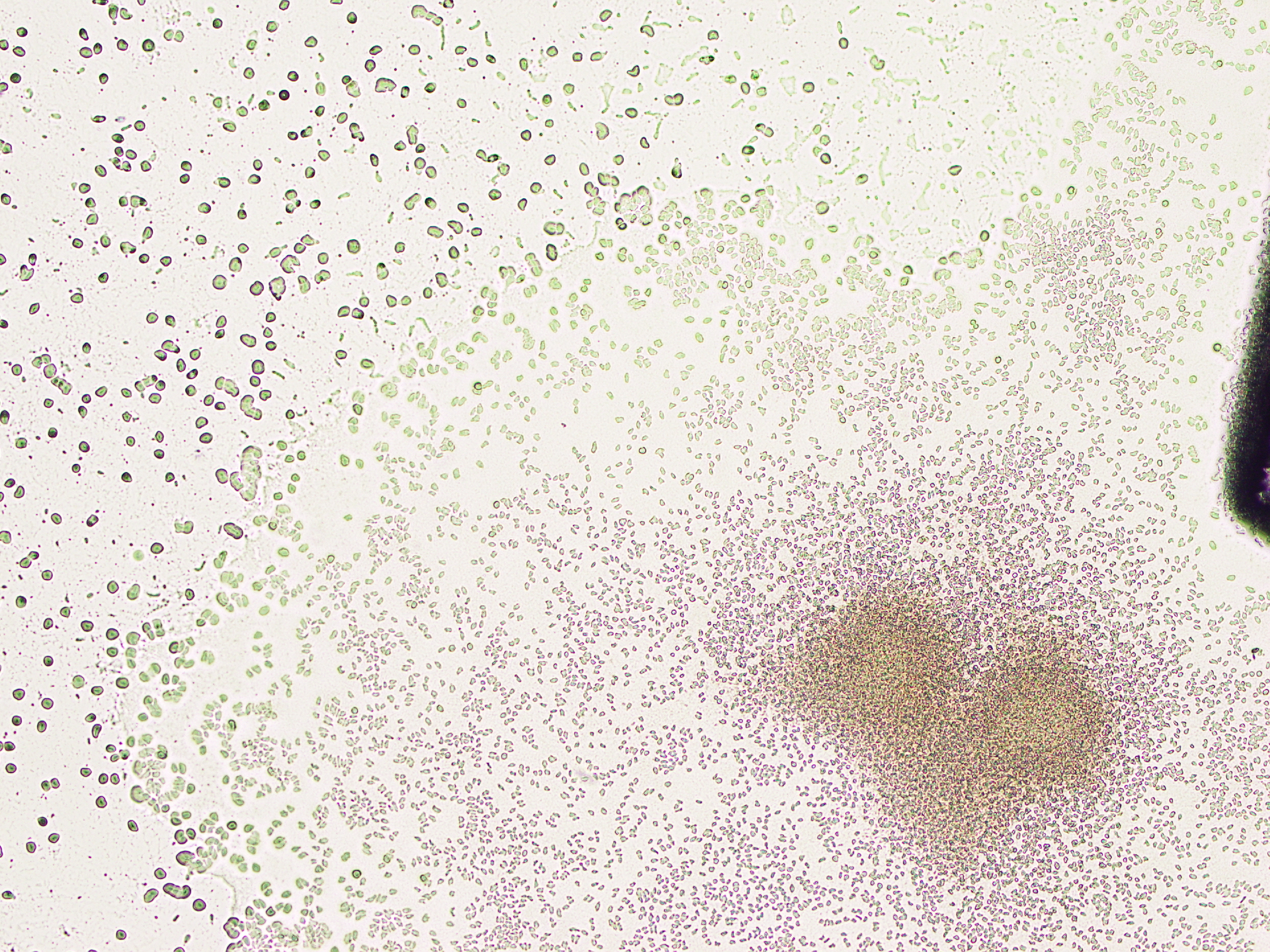

Supplement: Supplementary file 1 — Supplementary file1 (ZIP 208058 KB) [file 11686_2025_1053_MOESM1_ESM.zip › Supplementary_Figure3_4_5_MicroscopyImages/Trophozoite-24.JPG]

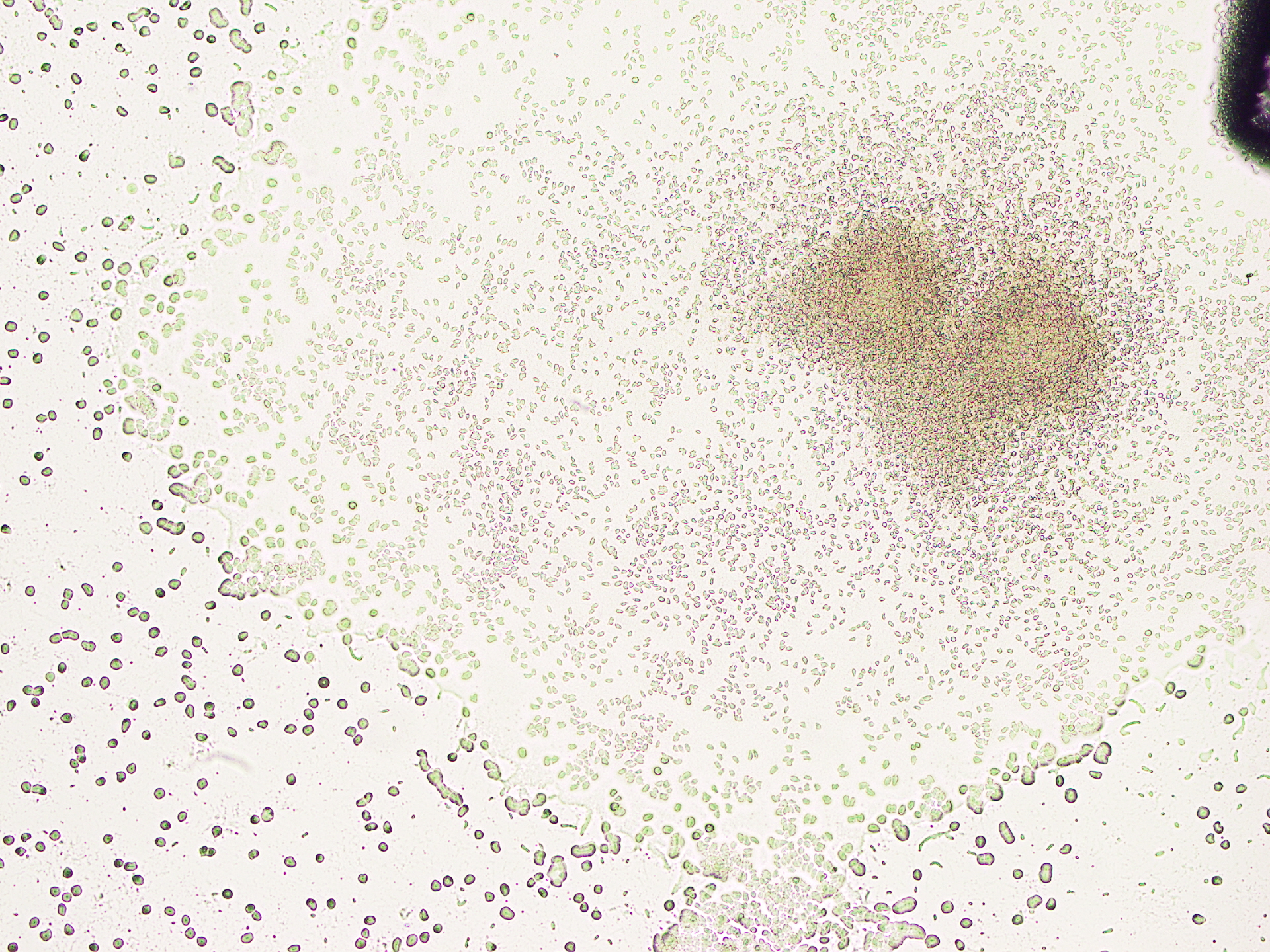

Supplement: Supplementary file 1 — Supplementary file1 (ZIP 208058 KB) [file 11686_2025_1053_MOESM1_ESM.zip › Supplementary_Figure3_4_5_MicroscopyImages/Trophozoite-25.JPG]

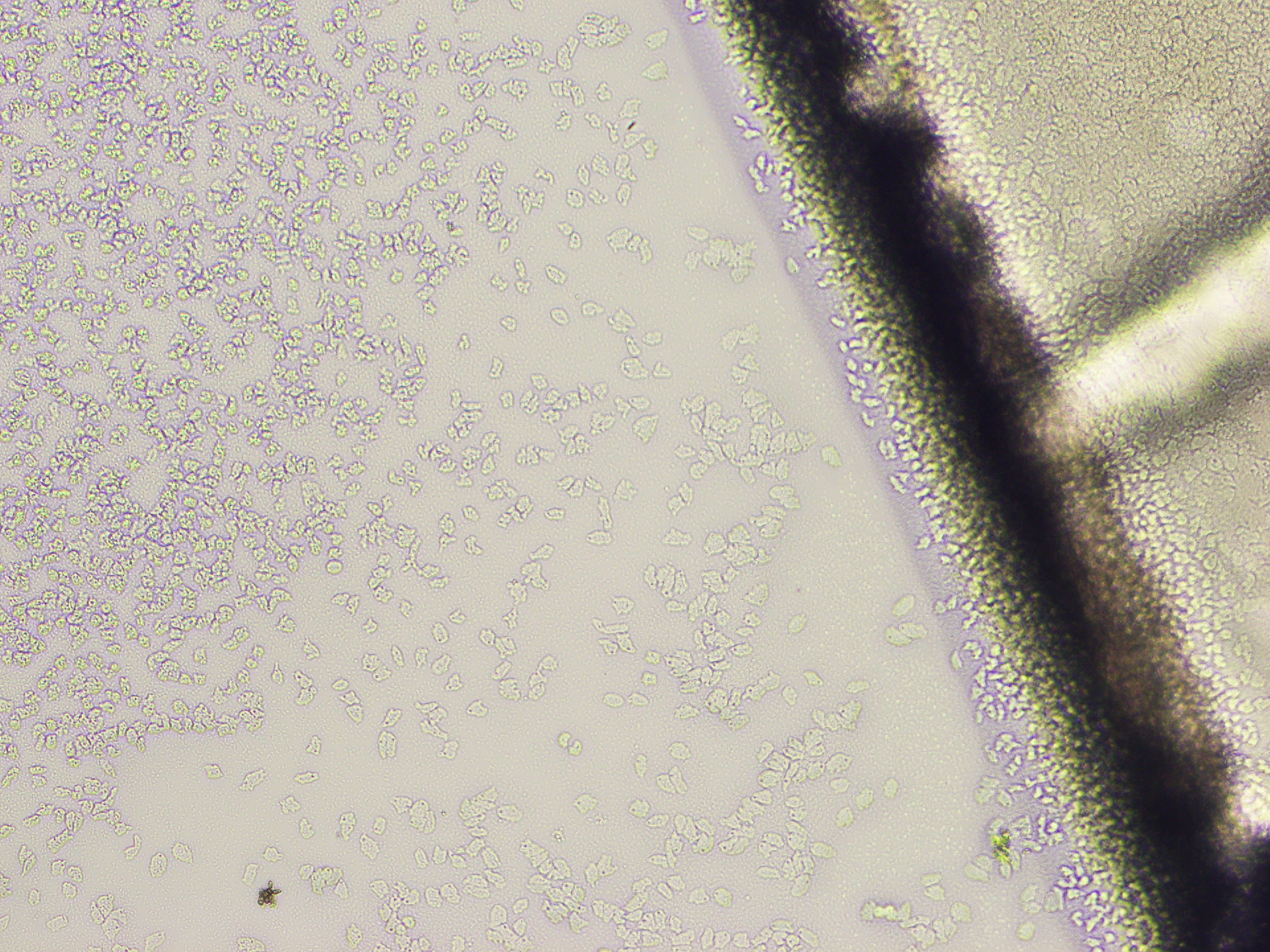

Supplement: Supplementary file 1 — Supplementary file1 (ZIP 208058 KB) [file 11686_2025_1053_MOESM1_ESM.zip › Supplementary_Figure3_4_5_MicroscopyImages/Trophozoite-26.JPG]

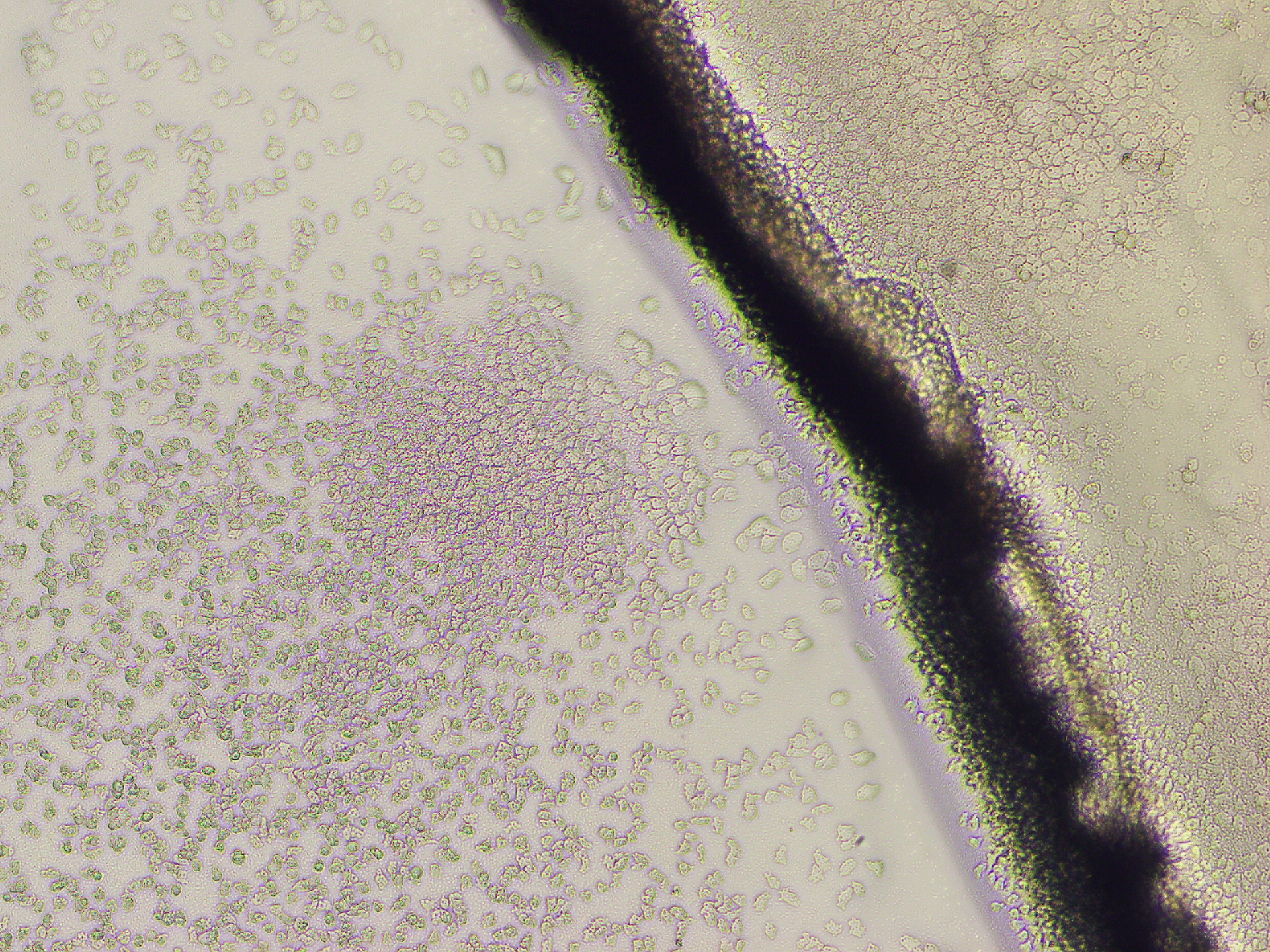

Supplement: Supplementary file 1 — Supplementary file1 (ZIP 208058 KB) [file 11686_2025_1053_MOESM1_ESM.zip › Supplementary_Figure3_4_5_MicroscopyImages/Trophozoite-27.JPG]

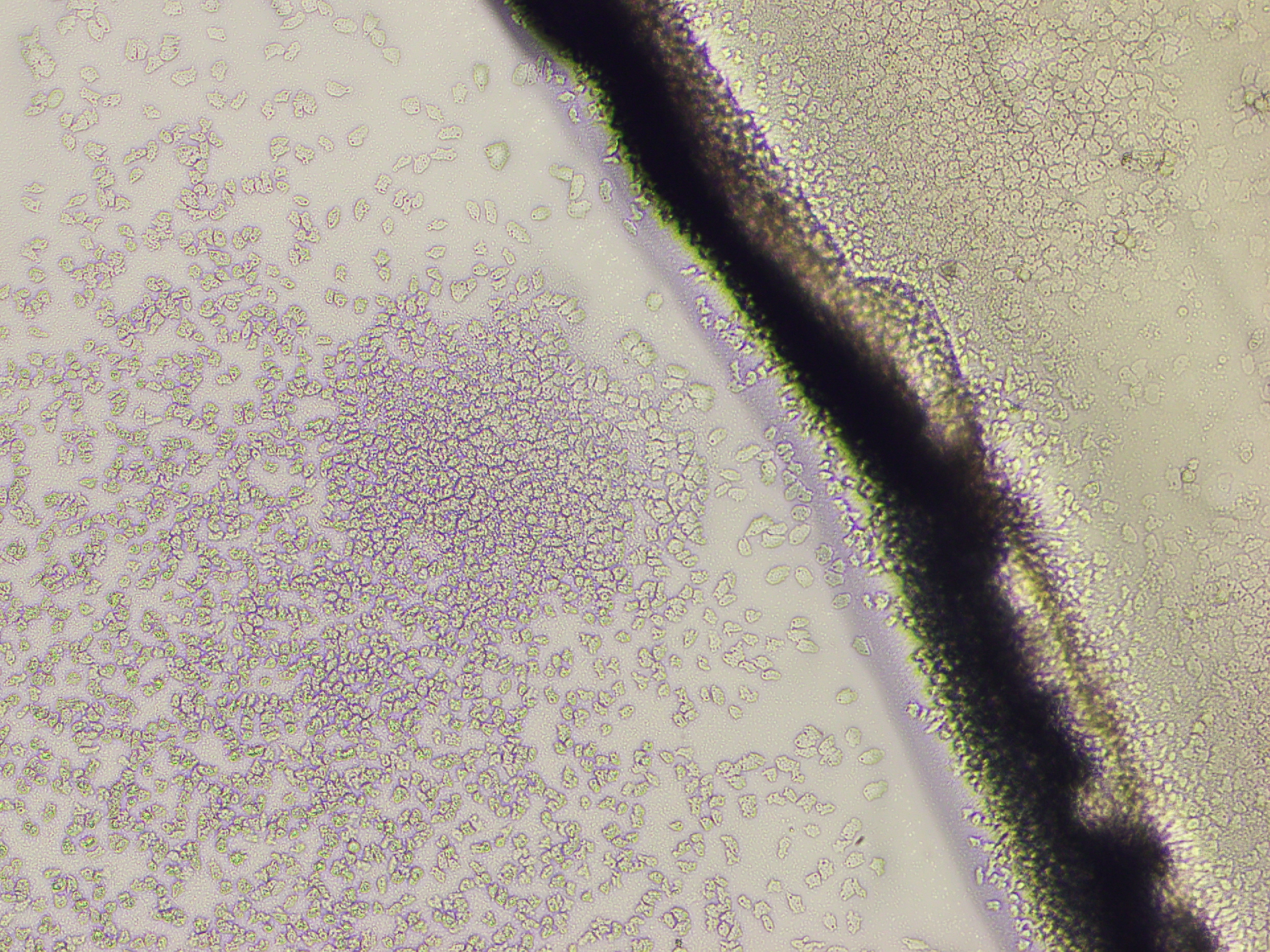

Supplement: Supplementary file 1 — Supplementary file1 (ZIP 208058 KB) [file 11686_2025_1053_MOESM1_ESM.zip › Supplementary_Figure3_4_5_MicroscopyImages/Trophozoite-28.JPG]

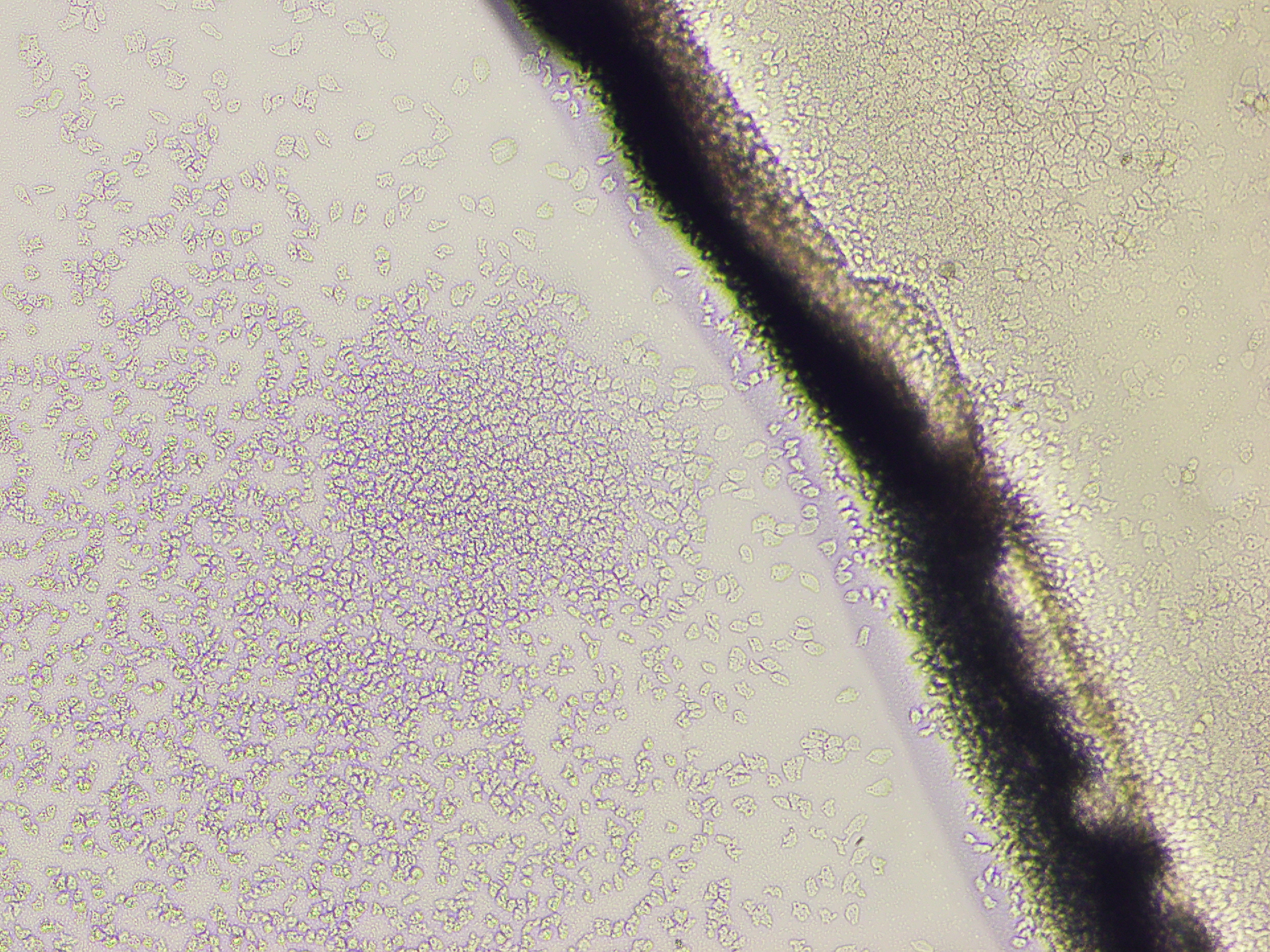

Supplement: Supplementary file 1 — Supplementary file1 (ZIP 208058 KB) [file 11686_2025_1053_MOESM1_ESM.zip › Supplementary_Figure3_4_5_MicroscopyImages/Trophozoite-29.JPG]

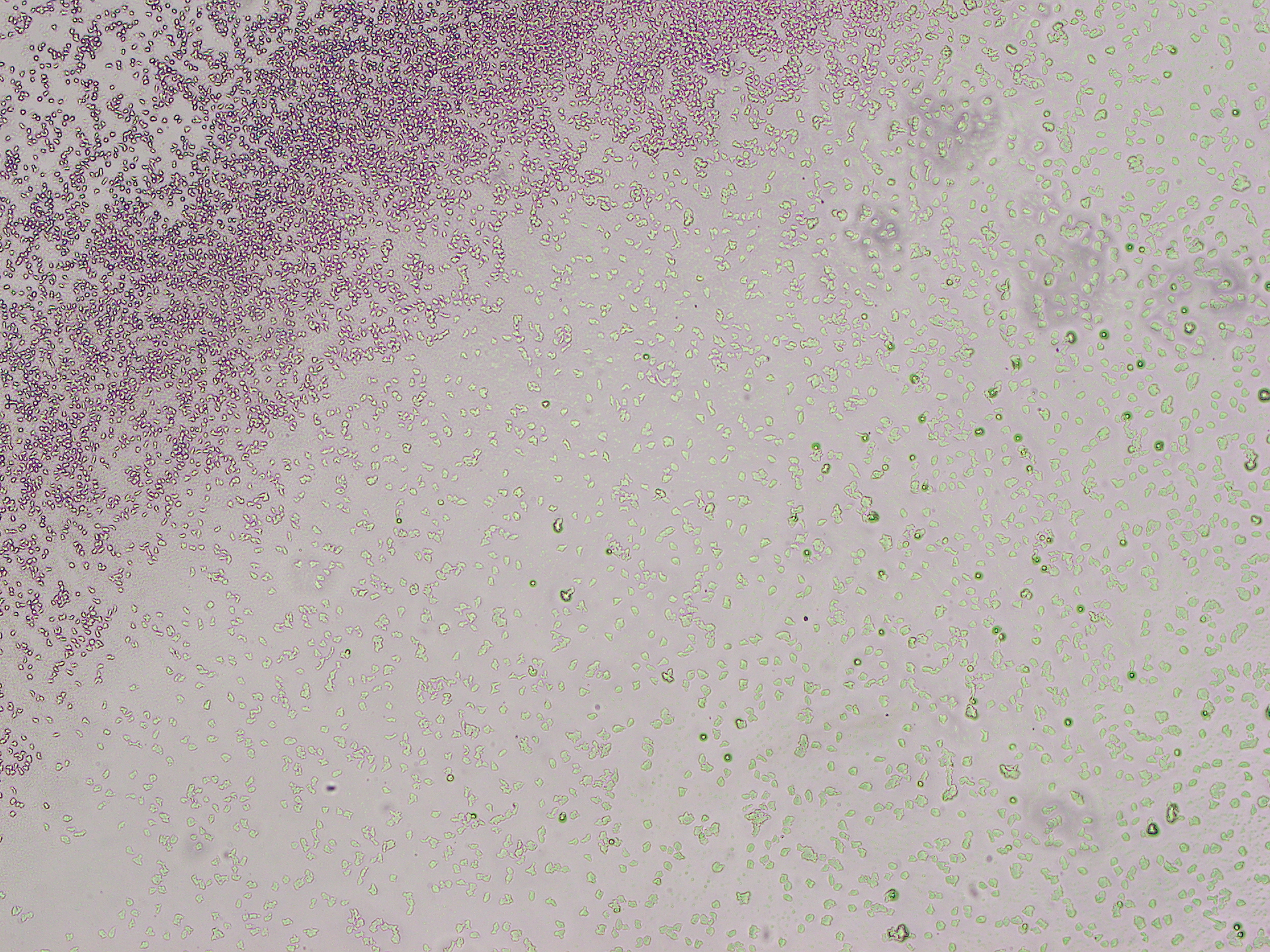

Supplement: Supplementary file 1 — Supplementary file1 (ZIP 208058 KB) [file 11686_2025_1053_MOESM1_ESM.zip › Supplementary_Figure3_4_5_MicroscopyImages/Trophozoite-3.JPG]

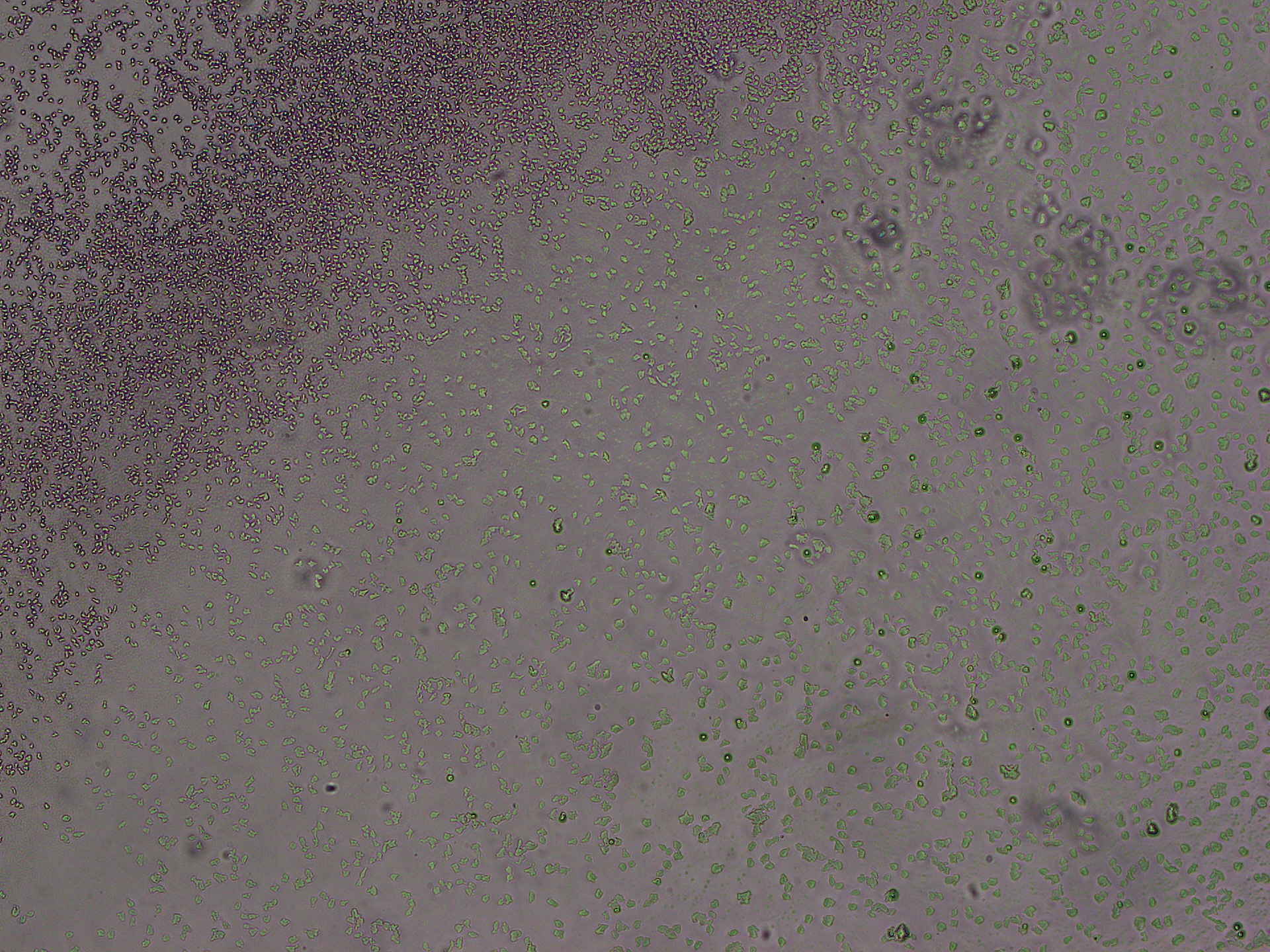

Supplement: Supplementary file 1 — Supplementary file1 (ZIP 208058 KB) [file 11686_2025_1053_MOESM1_ESM.zip › Supplementary_Figure3_4_5_MicroscopyImages/Trophozoite-4.JPG]

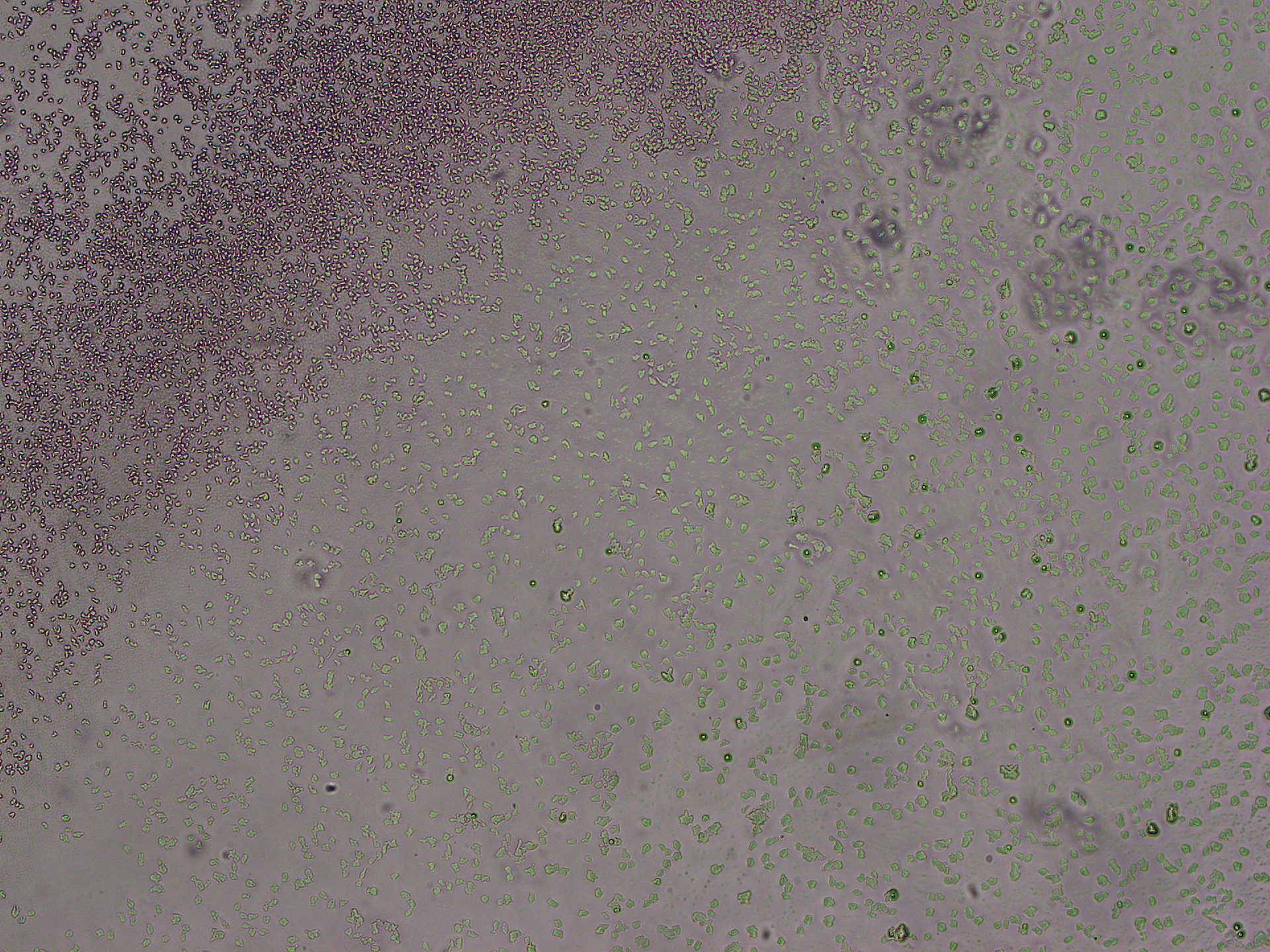

Supplement: Supplementary file 1 — Supplementary file1 (ZIP 208058 KB) [file 11686_2025_1053_MOESM1_ESM.zip › Supplementary_Figure3_4_5_MicroscopyImages/Trophozoite-5.JPG]

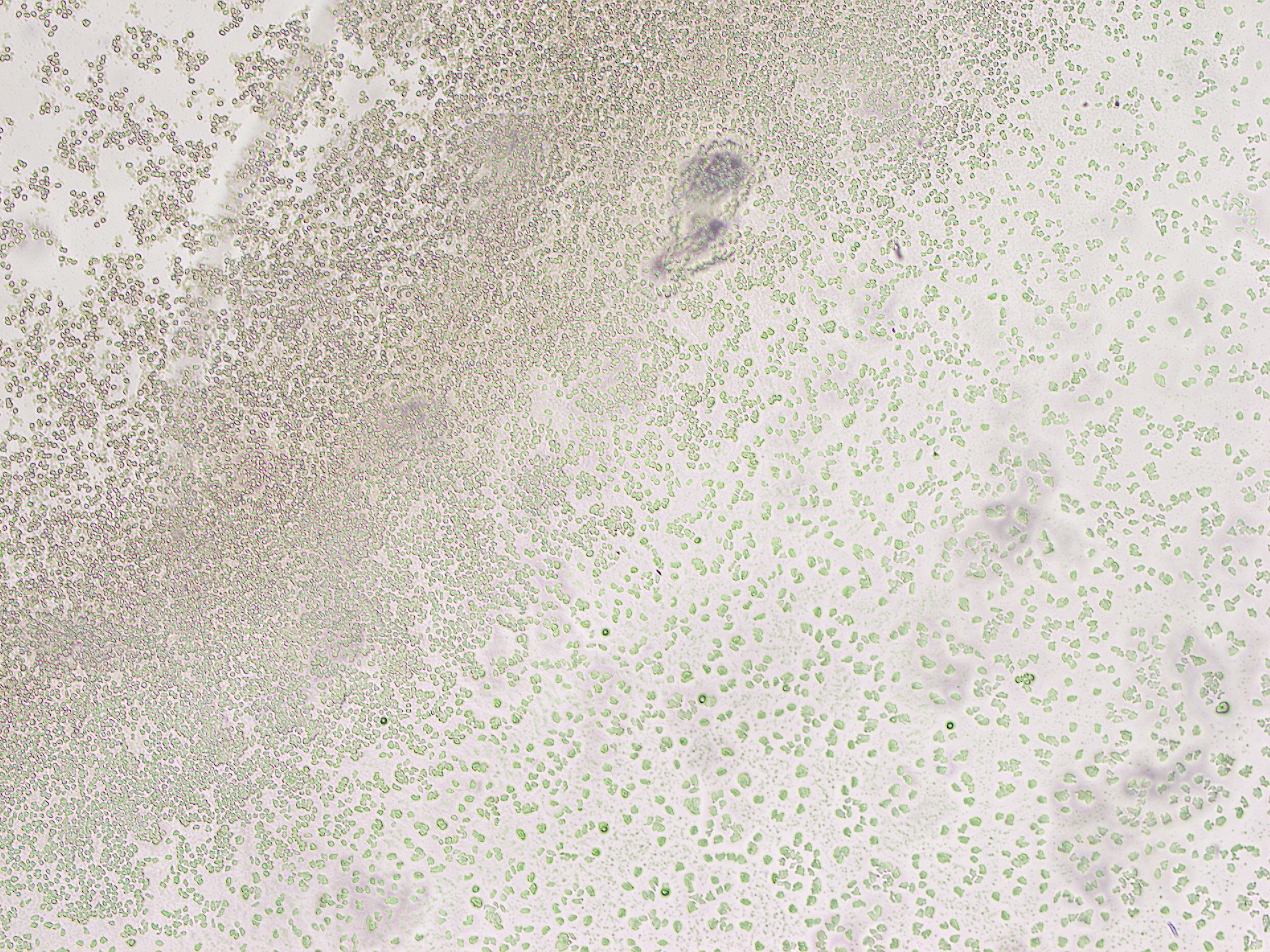

Supplement: Supplementary file 1 — Supplementary file1 (ZIP 208058 KB) [file 11686_2025_1053_MOESM1_ESM.zip › Supplementary_Figure3_4_5_MicroscopyImages/Trophozoite-6.JPG]

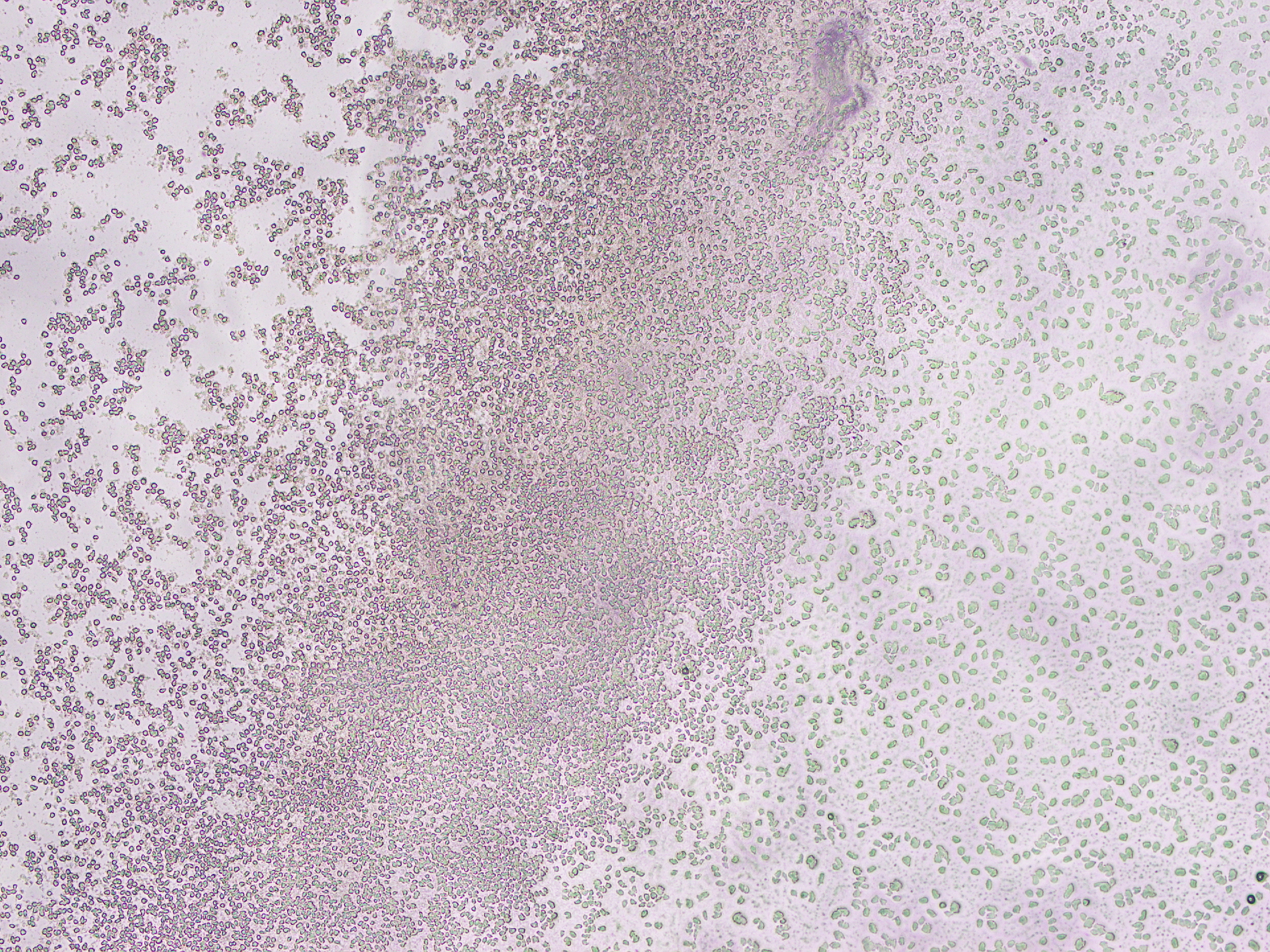

Supplement: Supplementary file 1 — Supplementary file1 (ZIP 208058 KB) [file 11686_2025_1053_MOESM1_ESM.zip › Supplementary_Figure3_4_5_MicroscopyImages/Trophozoite-7.JPG]

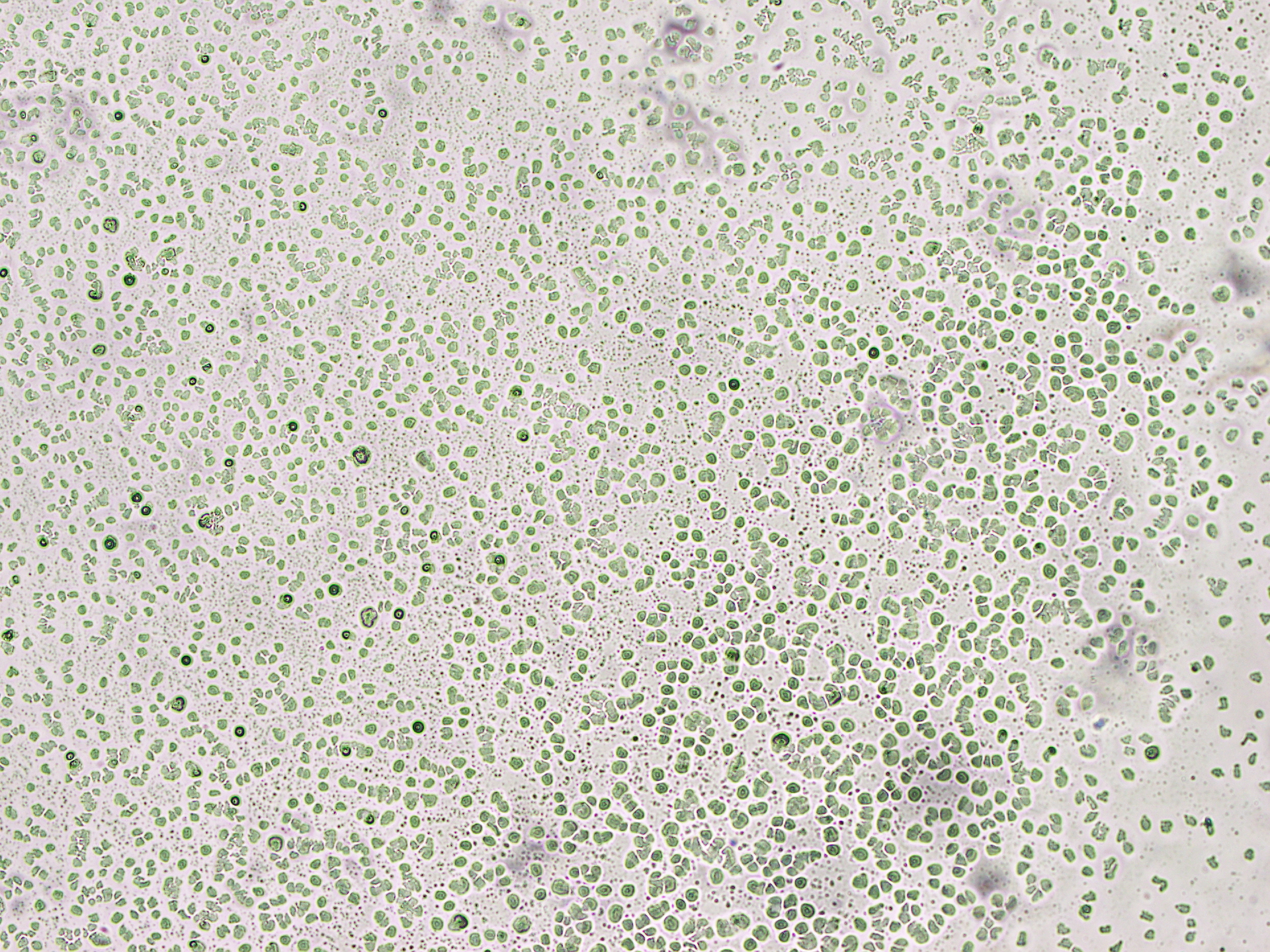

Supplement: Supplementary file 1 — Supplementary file1 (ZIP 208058 KB) [file 11686_2025_1053_MOESM1_ESM.zip › Supplementary_Figure3_4_5_MicroscopyImages/Trophozoite-8.JPG]

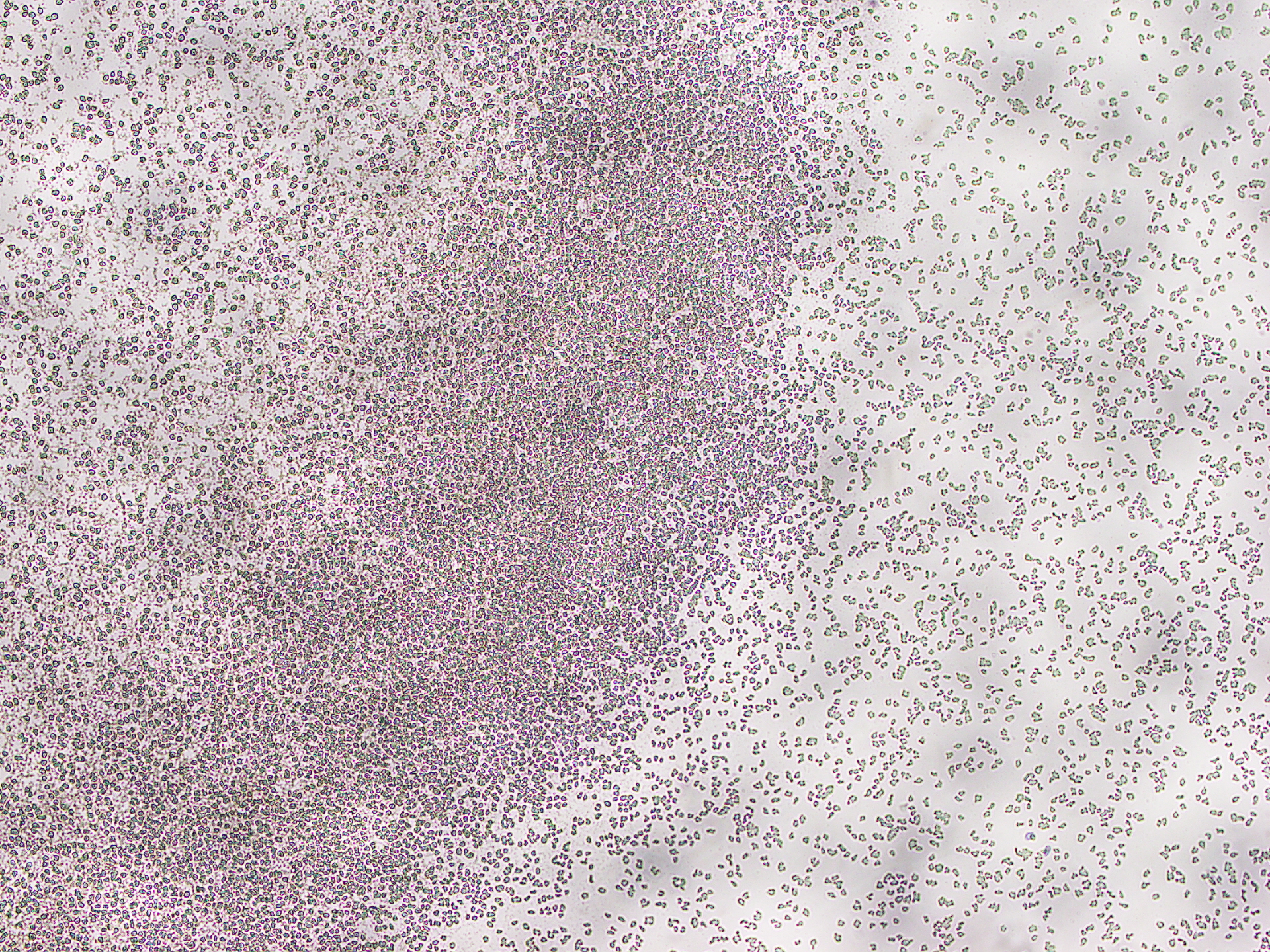

Supplement: Supplementary file 1 — Supplementary file1 (ZIP 208058 KB) [file 11686_2025_1053_MOESM1_ESM.zip › Supplementary_Figure3_4_5_MicroscopyImages/Trophozoite-9.JPG]
